# Early-life bovine respiratory virome analysis reveals phage-driven adaptation and cross-host evolutionary signatures

**DOI:** 10.1038/s41467-026-77597-8

**Published:** 2026-10-28

**Authors:** Yue Wang, Hanbeen Kim, Nilusha Malmuthuge, Mi Zhou, Yanhong Chen, Trevor W. Alexander, Edouard Timsit, Junhu Yao, Guanxiang Liang, Le Luo Guan

**Affiliations:** 1 Faculty of Land and Food Systems, The University of British Columbia, Vancouver, BC Canada; 2 College of Animal Science and Technology, Northwest A&F University, Yangling, Shaanxi China; 3 Faculty of Veterinary Medicine, University of Calgary, Calgary, AB Canada; 4 Department of Agricultural, Food & Nutritional Science, University of Alberta, Edmonton, AB Canada; 5 Lacombe Research and Development Centre, Agriculture and Agri-Food Canada, Lacombe, AB Canada; 6 Lethbridge Research and Development Centre, Agriculture and Agri-Food Canada, Lethbridge, AB Canada; 7 R&D Department, Ceva Santé Animale, Libourne, France; 8 Center for Infection Biology, School of Basic Medical Sciences, Tsinghua University, Beijing, China; 9 Tsinghua-Peking Joint Center for Life Sciences, Beijing, China

**Keywords:** Viral evolution, Microbial ecology, Virus-host interactions, Metagenomics

## Abstract

Past outbreaks of zoonotic viral infections highlight the importance of understanding respiratory virome ecology. As major food-producing animals with frequent human contact, cattle represent a critical host at the human-animal interface; however, the early-life establishment of their respiratory virome and its evolutionary connections to viruses from other hosts remain underexplored. Here, we longitudinally profile the upper respiratory tract (URT) virome of beef calves from birth to weaning using an optimized viral enrichment procedure and metagenomic workflow. Viral communities exhibited increased richness and turnover, reflecting ecological structuring during early life. Temperate phages dominate the URT phage community and share virulence- and mobility-associated genes with bacterial hosts, likely contributing to host adaptation and colonization persistence. Vertebrate-associated viruses emerge after the first week of life. Targeted reassembly from bovine URT viromes recovers near-complete betacoronavirus-like (CoV-D180) and adenovirus-like (AdV-D56) genomes positioned phylogenetically between bovine and human viral lineages. Competitive mapping of bovine-derived CoV-D180 and AdV-D56 to human respiratory metatranscriptomic datasets reveals genome-specific, regionally structured coverage patterns distinct from reference viruses, supporting shared sequence features in conserved genomic regions across host-associated viral lineages. Collectively, these findings reveal early-life assembly dynamics of the bovine respiratory virome and highlight cross-host signatures in a One Health framework.

## Abstract (web summary)

In this study, the authors use longitudinal metagenomics profiling to show that cattle upper respiratory viromes display age-dependent succession and phage-linked bacterial adaptation during early life, and harbor bovine-derived coronavirus and adenovirus genomes reflecting cross-host evolutionary signatures.

## Introduction

Respiratory tract (RT) health is critically important to mammalian well-being and public health. Recent repeated global outbreaks of viral infections, including influenza, SARS, and COVID-19, highlighted the profound impact of emerging RT viral pathogens on human health and global stability^1,2^. These events emphasize the need to better understand the ecological origins of RT viruses. Cattle are among the most economically important livestock species worldwide and provide a substantial proportion of animal-derived protein for humans^3,4^. Frequent human-cattle contact occurs across intensive production systems, smallholder farms, and the food processing chain, positioning cattle as potential hosts for zoonotic pathogens of relevance to human health^5,6^. Recent detection of highly pathogenic avian influenza A (H5N1) viruses in dairy cattle herds and documented infections in farm workers^7–9^, together with molecular evidence that human coronavirus OC43 likely originated from bovine coronavirus around 1890^10,11^, suggests the importance of systematically investigating viruses circulating in cattle at the human-animal interface.

Early life represents a critical window during which the RT microbiota and virome are established, and exposures during this period may shape long-term susceptibility to RT infection throughout the cattle production cycle^12,13^. However, compared to growing insights into RT bacterial ecology^14,15^, the early-life RT virome remains poorly understood. During early life, cattle face substantial RT disease burdens, including calf pneumonia and bovine respiratory disease, which are major drivers of antimicrobial use, compromised animal welfare, and economic loss in livestock industry^16–18^. Therefore, improvement of the understanding of cattle virome during early life, including its initial establishment, ecological succession, virus-bacteria interactions shaping the RT microecosystem, and potential cross-species evolutionary relevance, is essential to provide an ecological and evolutionary context relevant to One Health considerations.

Notably, one of the major challenges in studying RT virome lies in the low-biomass nature of respiratory samples, in which viral nucleic acids are present at very low abundance and are often obscured by overwhelming amounts of host and bacterial DNA^19,20^. In addition, conventional viromic metagenomic pipelines may fail to recover segmented RNA viruses when they are assembled as fragmented or low-coverage contigs, leading to their underrepresentation or loss in downstream analyses^21,22^.

In this work, we assessed the upper respiratory tract (URT) virome of beef calves from birth to weaning over the first six months of life using a viral particle enrichment protocol to characterize both DNA and RNA viruses and integrated URT bacteriome profiling to identify potential interactions between bacteriophages (phages) and hosts. In addition, we developed a dual-branch bioinformatic pipeline to separately resolve the taxonomic composition, temporal dynamics, and functional profiles of phages and vertebrate-associated viruses in the bovine URT. Together, this study provides new insights into the ecological assembly and cross-host evolutionary signatures of the ruminant URT virome during early life.

## Results

### Combined metagenomic and virome sequencing reveals the early-life bovine URT viral and bacterial community

**Fig. 1:**
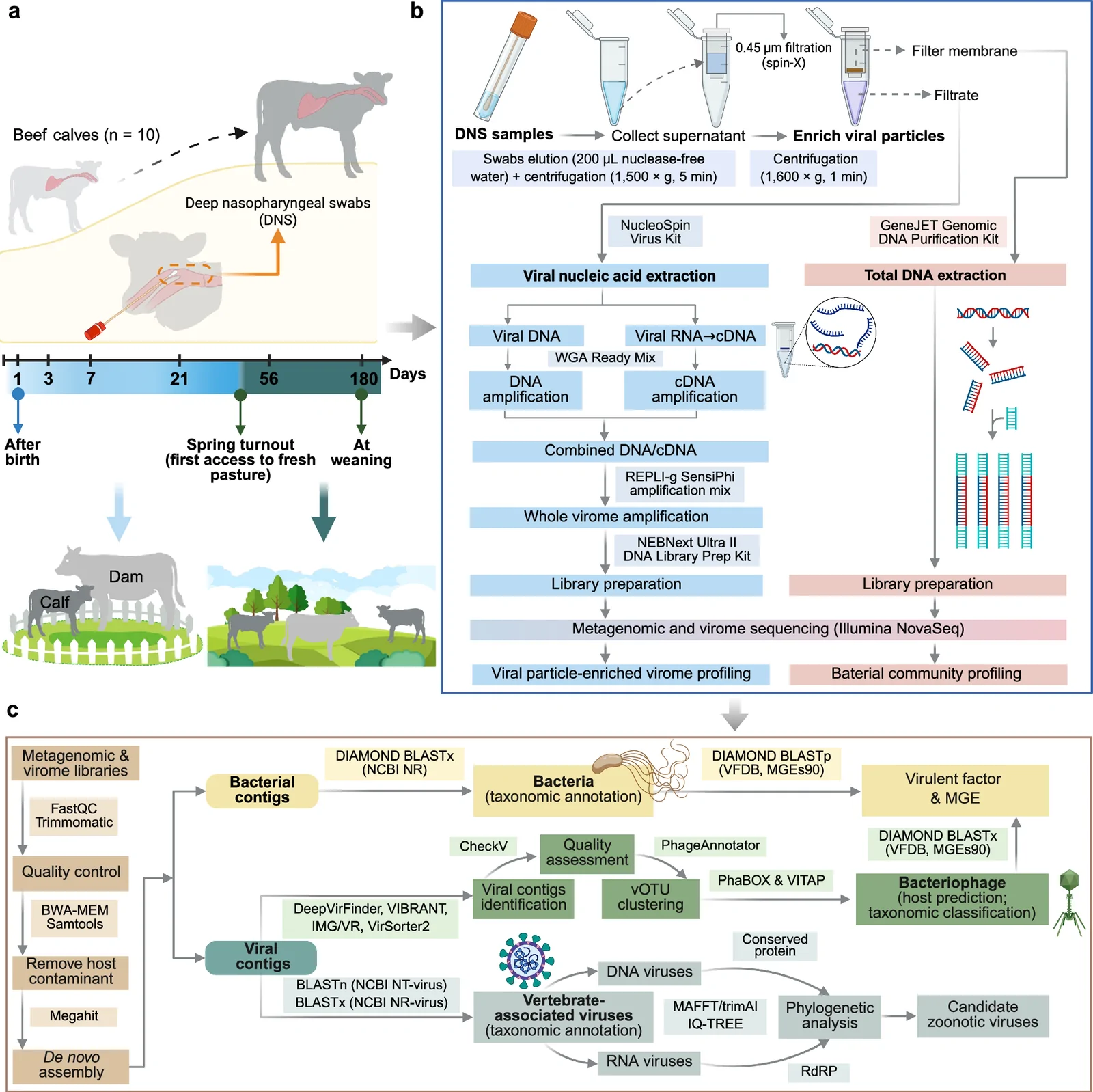
Experimental design and metagenomic analysis workflow for the calf upper respiratory tract (URT) virome and bacteriome. **a** Longitudinal sampling design. Deep nasopharyngeal swabs (DNS) samples were collected from ten beef calves at six developmental stages (Days 1, 3, 7, 21, 56, and 180 after birth), spanning key early-life events from birth to weaning. **b** Sample processing and sequencing workflow. DNS samples were processed in parallel for viral and bacterial community profiling, including viral particle enrichment followed by viral nucleic acid extraction for virome analysis and bacterial DNA extraction for metagenomic profiling. Separate library preparation workflows were applied for virome and bacteriome sequencing. **c** Bioinformatic analysis pipeline. Quality-controlled metagenomic and virome reads were assembled and analyzed through parallel bacterial and viral workflows. Bacterial contigs were used for taxonomic annotation and screening of virulence factors (VFs) and mobile genetic elements (MGEs). Viral contigs were processed through separate bacteriophage and vertebrate-associated virus branches for viral identification, quality assessment, vOTU clustering, taxonomic annotation, host prediction, phylogenetic analysis, and candidate zoonotic virus screening. (Created in BioRender. Leluo, G. (2026) https://BioRender.com/pqkehcw).

We longitudinally monitored ten newborn beef calves from birth to weaning over the first six months of life. Deep nasopharyngeal swabs (DNS) were collected across six early-life stages, yielding 60 samples in total (Fig. 1a). Prior to metagenomic sequencing, each DNS sample was performed viral particle enrichment (Fig. 1b), followed by a dual-branch bioinformatic pipeline to characterize virome and bacteriome (Fig. 1c). Shotgun metagenomic sequencing of 60 DNS samples yielded 5.32 billion raw reads, with an average of 88.67 ± 3.12 million reads (mean ± SEM) per sample. After quality filtering, 4.90 billion high‑quality reads (81.67 ± 2.87 million per sample) were retained and subjected to de novo metagenomic assembly, generating 87,836 contigs (1464 ± 175.9 per sample) (Supplementary Data 1–3).

Virome sequencing produced 5.30 billion raw reads (88.26 ± 3.17 million reads per sample), of which 4.27 billion reads (71.76 ± 2.73 million/sample) were retained as high-quality reads after filtering. De novo assembly produced 24,771 viral contigs in total (412.9 ± 68.5 contigs/sample; Supplementary Datas 4–6). Negative controls (NCs) samples showed lower sequencing output than DNS samples (Supplementary Datas 1–6). Using the same filtering and annotation criteria, 10,709 and 124 bacterial contigs were retained from the DNS and NCs samples, respectively (Supplementary Data 7). Most bacterial taxa were DNS-specific (*n* = 2120), whereas DNS/NCs-overlapped taxa were classified as DNS-dominant, Undetermined, or NC-dominant taxa based on contig representation and alignment quality (Supplementary Data 8 and Supplementary Fig. 1). Phage profiling using DeepVirFinder, VirSorter2, VIBRANT, and IMG/VR identified 1079 and 33 non-redundant candidate viral contigs in DNS and NC samples, respectively, with minimal cross-tool overlap among NC contigs (Supplementary Data 9–12 and Supplementary Fig. 2a, b). CheckV classified DNS contigs as low-quality (13.48%) or not-determined (19.01%), while all NC contigs were low-quality (33.33%) or not-determined (66.67%) and were excluded from downstream analyses (Supplementary Data 13 and Supplementary Fig. 2c, d). Among the 728 viral contigs retained after quality filtering, 427 were identified as phage contigs. These phage contigs were further clustered into 394 non-redundant viral operational taxonomic units (vOTUs) based on 95% average nucleotide identity (ANI) and 85% alignment coverage (Supplementary Data 14). Parallel screening of assembled contigs using the NCBI NT and NR virus databases identified 671 additional viral contigs as vertebrate-associated viruses (Supplementary Data 15). Species-level comparison showed that most DNS-derived candidate vertebrate-associated viruses were absent from the NC samples. Only bovine rhinitis B virus (BRBV) and Mamastrovirus 3 (MAstV-3) overlapped between DNS and NCs samples (Supplementary Fig. 2e). Based on contig representation and alignment metrics, BRBV was classified as a DNS-dominant DNS/NCs-overlapped virus and retained for downstream analysis, whereas MAstV-3 was considered an NCs-associated background viral signature and excluded from any analyses and main biological interpretations (Supplementary Fig. 2f–m and Supplementary Data 15). After removal of redundant viral contigs detected in both the phage-associated and vertebrate-virus branches, a total of 1098 viral contigs were retained.

The application of the above dual-branch analytical pipeline generated a comprehensive catalog of the early-life URT microbiome. This framework enabled the integrated characterization of URT bacterial communities, phages, and vertebrate-associated DNA and RNA viruses captured from the same set of DNS samples (Fig. 1).

### Rapid early-life remodeling of the URT virome highlights broad viral diversity and extensively uncharacterized bovine-associated viruses

**Fig. 2:**
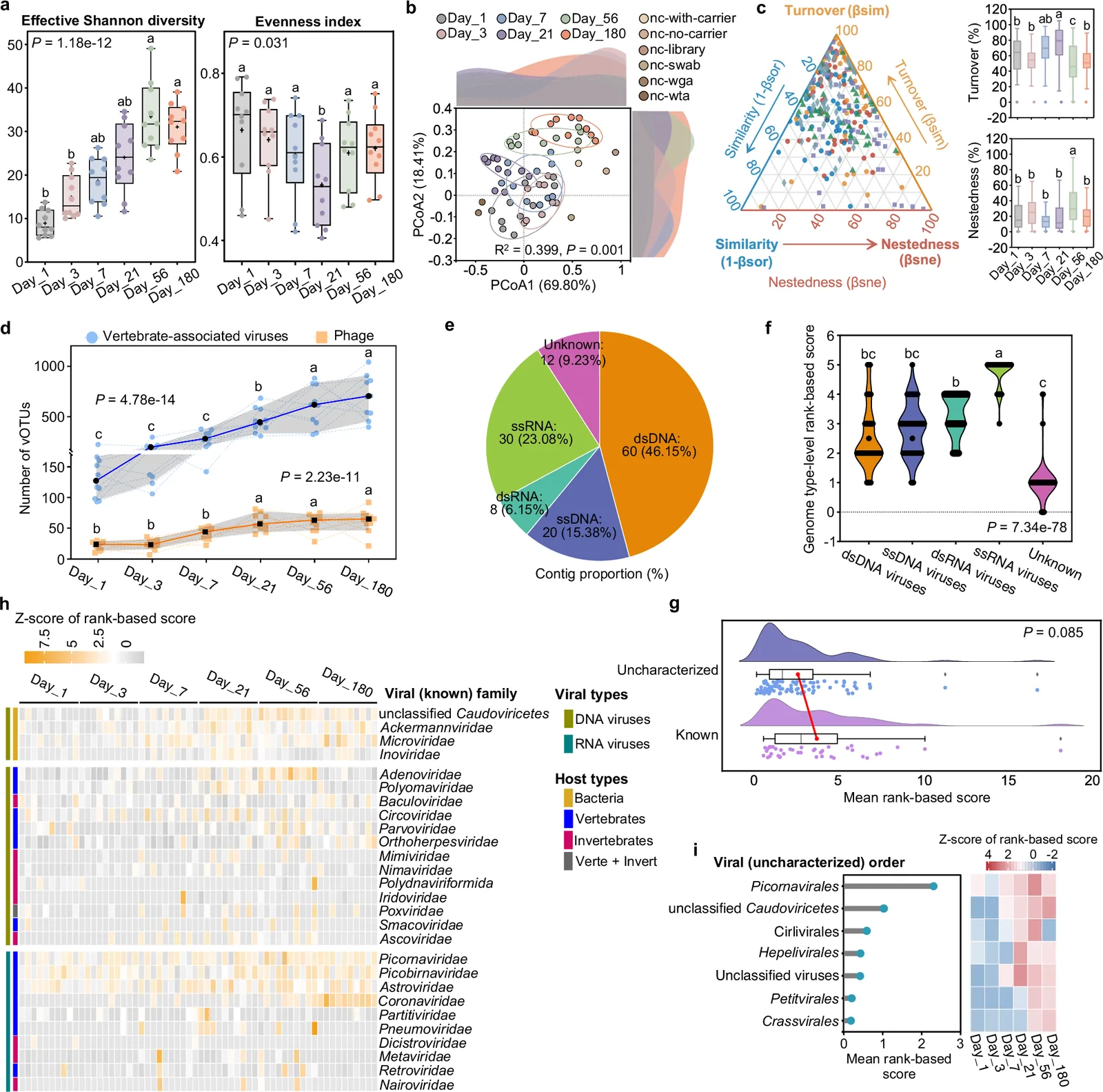
Overview of the upper respiratory tract (URT) virome in calves across early life. **a** Effective Shannon diversity and evenness index across six sampling time points. Each point represents one biologically independent deep nasopharyngeal swab (DNS) sample; *n* = 10 calves per time point. Box plots show the median, 25–75th percentiles, and whiskers extending to 1.5× interquartile range (IQR). **b** Principal coordinate analysis (PCoA) based on Spearman rank-correlation distances calculated from rank-order-transformed raw read-count profiles. Each point represents one sample; *n* = 60 DNS samples and *n* = 6 negative control (NC) samples. The effect of sampling time on DNS viral profiles was tested using PERMANOVA with 999 permutations constrained by calf identity. **c** Partitioning of beta diversity into similarity, turnover, and nestedness components using within-time-point pairwise comparisons. The ternary plot includes *n* = 270 pairwise comparisons in total; box plots summarize *n* = 45 comparisons per time point and are defined as in a. Beta diversity was partitioned into Sørensen dissimilarity (β_sor), turnover (β_sim), and nestedness-resultant components (β_sne) using the betapart package. **d** Number of detected phage and vertebrate-associated viral operational taxonomic units (vOTUs) across sampling time points. Points represent biologically independent DNS samples; *n* = 10 calves per time point. Lines show median values, and shaded areas indicate 95% confidence intervals (CI). **e** Genome-type composition of viral contigs. **f** Genome type-level rank-based scores across DNS sample profiles. Violin plots show distributions, with internal box plots defined as in (**a**). Each point represents one DNS sample-level score for the indicated genome type; *n* = 60 DNS samples per genome type. Differences among genome types were tested using Friedman tests followed by Dunn’s multiple-comparison tests. Different letters indicate significant differences between genome types after multiple-comparison correction. **g** Mean rank-based scores of known (*n* = 40) and uncharacterized (*n* = 89) contigs. Each point represents one contig. Differences were tested using a two-tailed Mann-Whitney test. **h** Z-scores of family-level rank-based scores for known viral families, annotated by viral genome type and host range. **i** Mean rank-based scores and temporal patterns of rank-based scores for uncharacterized viral order-level groups. Rank-based scores were calculated from within-sample rankings of raw read counts, with undetected features retained as zero. Time-point differences in (**a**, **c** and **d**) were tested using Friedman tests followed by Dunn’s post hoc multiple comparisons, with Benjamini-Hochberg FDR correction. Different letters indicate significant differences between time points; exact *P* values of (**a**, **c**, **d**, **f** and **g**) are provided in the Source Data file. Source data are provided as a Source Data file.

The temporal dynamics of the URT viral community showed an increase in effective Shannon diversity from Day 1–56 (*P* < 0.0001), while evenness decreased from Day 1–21, followed by an increase at later time points (*P* = 0.031) (Fig. 2a). principal coordinate analysis (PCoA) based on rank-order dissimilarities revealed significant age-dependent structuring of URT viral profiles (PERMANOVA, *R*^2^ = 0.399, *P* = 0.001) (Fig. 2b). Partitioning of between-sample β-diversity showed that viral community differences were primarily driven by turnover (β_sim), with most pairwise comparisons clustering in the turnover-dominated region of the ternary space (Fig. 2c). Comparisons involving early time points (Day 1–21) exhibited significantly higher turnover (*P* < 0.05), whereas Day 56 exhibited increased nestedness (β_sne) accompanied by reduced turnover compared with earlier stages (*P* < 0.05) (Fig. 2c). The number of detected viral vOTUs increased with calf age, with both vertebrate-associated viruses and phages showing gradual increases from Day 1–180; vertebrate-associated viruses consistently represented the larger component of the URT virome (Fig. 2d).

In the calf URT virome, double-stranded DNA (dsDNA) viruses represented the largest proportion of viral contigs (46.15%), while single-stranded RNA (ssRNA) viruses ranked highest based on rank-based scores calculated from within-sample rankings of raw read counts (Fig. 2e, f). Comparison of 1098 viral contigs against the IMG/VR database showed that known viral contigs tended to have higher mean rank-based scores than uncharacterized viral contigs (FDR-adjusted *P* = 0.085) (Fig. 2g). Among known phage-related families, unclassified *Caudoviricetes*, *Ackermannviridae*, *Microviridae*, and *Inoviridae* showed increased rank-based scores from early life to later developmental stages (Fig. 2h and Supplementary Data 16). Within the vertebrate-associated virome, several DNA viral families showed age-associated shifts in family-level rank-based scores, such as *Adenoviridae*, *Circoviridae*, and *Orthoherpesviridae*, with *Adenoviridae* reaching its highest score at Day 56 (FDR-adjusted *P* < 0.05; Fig. 2h and Supplementary Data 17). Among RNA viral families, *Coronaviridae* showed the highest rank-based score at Day 180 (FDR-adjusted *P* < 0.05), while *Picornaviridae* and *Picobirnaviridae* maintained high rank-based scores across multiple time points (Fig. 2h and Supplementary Data 17). Approximately 28% of vOTUs showed <90% nucleotide identity and/or <75% alignment coverage to IMG/VR reference sequences, indicating limited representation of these viruses in current reference databases. These vOTUs were taxonomically assigned mainly to higher ranks, including *Picornavirales*, unclassified *Caudoviricetes*, *Cirlivirales,* etc., and showed generally low rank-based scores across developmental stages (Fig. 2i). These observations emphasizes that the early-life calf URT harbors a broad and largely unexplored viral reservoir, suggesting the need for further characterization of these pioneer viruses that may play a role in the URT microbiome establishment.

### Early-life URT phages exhibit host-dependent assembly and lifestyle-driven temporal dynamics

**Fig. 3:**
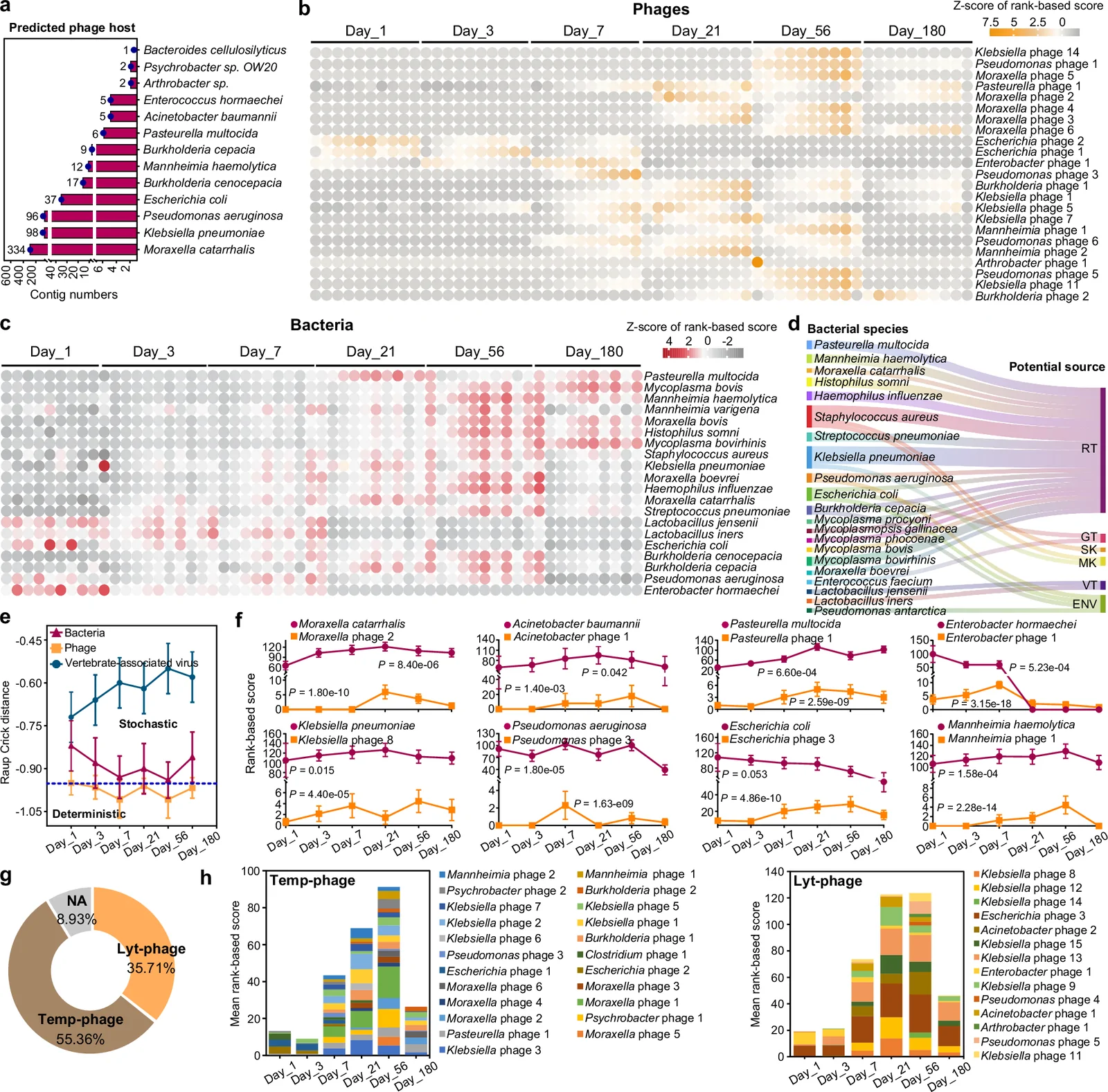
Host-dependent assembly and lifestyle-associated temporal dynamics of early-life URT phages in calves. **a** Predicted bacterial hosts of URT phages identified across early life. Bars indicate the number of phage contigs associated with each predicted bacterial host. **b** Temporal dynamics of representative URT phages from birth to Day 180, shown as Z-scores of rank-based scores across samples. **c** Temporal dynamics of representative bacterial species in the URT across early life, shown as Z-scores of rank-based scores. **d** Potential source environments of early-life URT bacterial species inferred from curated database information, including respiratory tract (RT), gastrointestinal tract (GT), skin (SK), milk (MK), vaginal tract (VT), and environmental (ENV) sources. **e** The Raup-Crick dissimilarity analysis showing community assembly patterns for bacteria, phages, and vertebrate-associated viruses across early life. Data are mean ± SEM; *n* = 10 biologically independent DNS samples at each time point for bacteria, phages, and vertebrate-associated viruses. The dashed line indicates the threshold separating deterministic and stochastic assembly. **f** Representative temporal trajectories of rank-based scores for dominant bacterial species and their predicted corresponding phages. Data are mean ± SEM; *n* = 10 biologically independent DNS samples per time point. Time-point differences were tested using Friedman tests followed by Dunn’s post hoc multiple comparisons, with Benjamini-Hochberg FDR correction. Exact adjusted *P* values are provided in the Supplementary Data and Source Data file. **g** Proportional composition of temperate (Temp) and lytic (Lyt) phages identified in the URT virome. **h** Temporal patterns of Temp- and Lyt-phages across early life, shown as mean rank-based scores at each sampling time point. Rank-based scores were calculated from within-sample rankings of raw read counts, with undetected features retained as zero. Source data are provided as a Source Data file.

Phage host prediction indicated that early-life URT phages were predominantly associated with *Moraxella catarrhalis*, accounting for 334 predicted host-linked contigs, followed by *Klebsiella pneumoniae*, *Pseudomonas aeruginosa*, and *Escherichia coli* (Fig. 3a, Supplementary Fig. 3a and Supplementary Data 18). Despite their generally low detection levels after birth, phages were consistently detectable in the calf URT and exhibited clear age-specific colonization patterns. Most phages emerged after Day 3 and showed increased rank-based scores during Day 7–56 (FDR-adjusted *P* < 0.001; Supplementary Data 19), particularly those predicted to infect *Klebsiella*, *Moraxella*, *Pseudomonas*, and *Mannheimia* (Fig. 3b). In contrast, *Escherichia* phages showed higher rank-based scores during the first three days of life, followed by a marked decline thereafter. (Fig. 3b and Supplementary Fig. 3b).

Phage replication is fundamentally constrained by the availability and dynamics of their host bacteria, which displayed pronounced early-life restructuring in the URT. Similar to the virome, the bacteriome also underwent pronounced early-life restructuring. Bacterial richness increased steadily throughout the first 56 days, accompanied by an early decline and later recovery of evenness (Supplementary Fig. 4a). Additionally, the URT bacterial community composition was affected by calf age (*R*^2^ = 0.363, *P* = 0.001), especially as the Day 56 and 180 bacterial communities differed from those of earlier time points (Supplementary Fig. 4b). β-diversity partitioning showed that, although bacterial communities shared a substantial proportion of taxa across samples, the observed between-sample differences were primarily driven by taxon turnover (β_sim) (Supplementary Fig. 4c). When the ecological processes driving the assembly of the URT virome and bacteriome was assessed, only the phage community exhibited deterministic assembly, while the vertebrate-associated viral communities remained predominantly stochastic (Fig. 3e and Supplementary Data 20). The bacterial community displayed a stochastic assembly during the first week of life and later on a deterministic assembly (Fig. 3e and Supplementary Data 20).

The colonization dynamics of the URT bacterial community (Supplementary Fig. 4d and Supplementary Data 21–23) revealed that major phage hosts, including *M. catarrhalis*, *K. pneumoniae*, *P. aeruginosa*, *M. haemolytica*, *P. multocida*, and *B. cepacia,* were persistent residents of the URT of beef calves. Among these, *M. haemolytica*, and *P. multocida*, two opportunistic pathogens associated with bovine RT disease dominated the URT bacterial community throughout pre-weaning period (Fig. 3c). Moreover, phages targeting these bacterial taxa closely mirrored host population dynamics, exhibiting synchronized expansion with their bacterial hosts (Fig. 3f). However, pioneer URT taxa detected only during the first week of life, such as *E. coli*, *Enterobacter hormaechei*, and *Lactobacillus iners*, originated predominantly from the gastrointestinal and vaginal tracts, as well as environmental sources (Fig. 3c, d and Supplementary Fig. 4e). Phages infecting *Enterobacter* and *Psychrobacter* displayed delayed increases in rank-based detection scores relative to their hosts (Fig. 3f and Supplementary Fig. 5), consistent with predator-prey like temporal offsets. Phages targeting *E. coli* and *K. pneumoniae* exhibited both synchronous and lagged dynamics across time, indicating flexible and potentially context-dependent interaction strategies (Fig. 3f).

When the phage lifestyles were further examined to determine whether lifestyle differences could account for these distinct temporal phage-host interaction patterns, we found that temperate (Temp) phages dominated the early-life URT virome, accounting for 55.36% of all identified phages, whereas lytic (Lyt) phages represented 35.71% (Fig. 3g). Tempe-phages detected in the URT mainly included phages infecting M. catarrhalis, *P. aeruginosa*, *M. haemolytica,* etc., whereas Lyt-phages were mainly represented by phages associated with *K. pneumoniae*, *E. coli* and *E. hormaechei*. Phages infecting *E. coli* and *K. pneumoniae* were distributed across both temperate and lytic lifestyles, consistent with their mixed, host-decoupled temporal patterns described above (Fig. 3h).

### Temperate phages dominate virulence and mobility gene pools associated with colonization persistence and host adaptation of early-life URT opportunistic pathogens

**Fig. 4:**
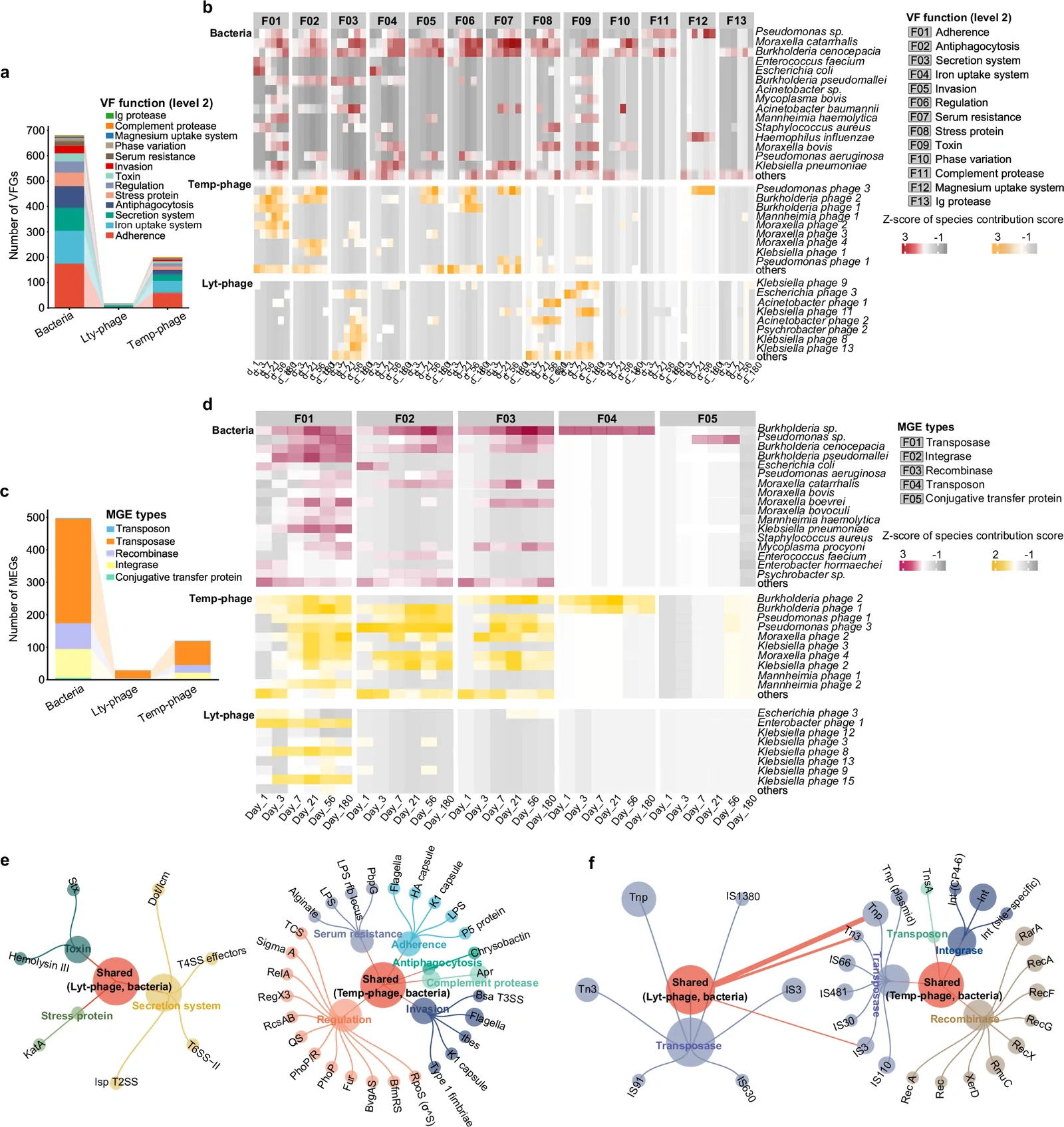
Shared virulence- and mobility-associated gene pools between respiratory bacteria and phages in the calf URT. **a** Distribution of virulence factor genes (VFGs) across bacteria, Lyt-phages, and Temp-phages identified in the calf URT. Bar plots show the total number of VFGs detected in each group. **b** Heatmap showing the temporal contribution patterns of virulence factor functions detected in representative bacterial species, Temp-phages, and Lyt-phages from Day 1–180. Rows represent bacterial species or phages, and columns correspond to VFs' function categories. Color intensity represents the Z-score of species-level contribution scores. **c** Distribution of mobile genetic elements (MGEs) across bacteria, Lyt-phages, and Temp-phages in the calf URT. Bar plots show the total number of MGEs detected in each group. **d** Heatmap showing the temporal contribution patterns of MGE types detected in representative bacterial species, Temp-phages, and Lyt-phages across sampling time points. Rows represent bacterial species or phages, and columns correspond to MGE categories. Color intensity represents the Z-score of species-level contribution scores. **e**, **f** Network analyses showing shared VFs (**e**) and MGEs (**f**) between phages and their predicted bacterial hosts in the calf URT. Nodes represent functional categories or gene families, and edges indicate shared occurrence between bacterial and phage genomes. Source data are provided as a Source Data file.

Knowledge on shared virulence- and mobility-associated genes between phages and their host is vital to understand how the opportunistic pathogens colonizing the URT acquire traits linked to persistence and adaptation. Bacterial contigs from the beef calf URT microbiome harbored a broad repertoire of virulence factor genes (VFGs), encompassing more than 700 genes across multiple functional categories, with those involved in adherence, iron acquisition, and secretion systems being most frequently detected (Fig. 4a). These VFGs mapped primarily to *Pseudomonas*, *Moraxella*, *Burkholderia*, *Mannheimia*, *Pasteurella*, and *Klebsiella*, consistent with their dominance in the URT (Fig. 4b and Supplementary Data 24). Many VFGs, particularly adherence, anti-phagocytosis, invasion, and complement protease functions, showed persistent contribution patterns across the pre-weaning period, especially in *M. catarrhalis* and *B. cenocepacia*. In contrast, toxin-related and phase variation genes occurred sporadically and showed weaker contributions, mainly during the later stages, Day 21–180, and were primarily carried by *K. pneumoniae*, *M. bovis*, *M. haemolytica*, *M. catarrhalis*, and *B. cenocepacia* (Fig. 4b and Supplementary Data 25). Temp-phages carried a subset of VFGs linked to adherence, anti-phagocytosis, invasion, and regulatory functions (Fig. 4a). These were consistently detected across sampling timepoints and were associated with phages infecting *Burkholderia*, *Mannheimia*, and *Moraxella*, suggesting long-term maintenance of virulence-associated functions within temperate phage genomes. However, Lyt-phages showed a narrower VFG repertoire dominated by secretion system, stress response, and toxin genes, primarily linked to *Klebsiella* phages and sporadically to *Escherichia* phage (Fig. 4b, Supplementary Data 26 and 27). These signatures increased after Day 21, mirroring the delayed increase in Lyt-phage contribution scores. Bacterial hosts also contained the largest diversity of mobile genetic elements (MGEs), dominated by transposases, recombinases, integrases, and conjugative transfer proteins (Fig. 4c and Supplementary Data 28). These were consistently enriched in *Burkholderia*, *Pseudomonas*, *Moraxella*, *Klebsiella*, and *Mannheimia*, reflecting their high genomic plasticity and capacity for horizontal gene exchange (Fig. 4d and Supplementary Data 29). Temp-phages encoded multiple MGEs, particularly integrases, recombinases, and transposases, which were detected mainly after Day 3 and were carried by phages infecting *Burkholderia*, *Moraxella*, *Mannheimia*, and *Klebsiella*. These patterns suggest that Temp-phages may contribute to ongoing gene flow within the developing URT, while Lyt-phages contained fewer MGEs, largely restricted to transposases associated with *Klebsiella* and *Enterobacter* phages (Fig. 4d, Supplementary Data 30 and 31).

Gene-sharing network analysis identified distinct functional exchange patterns between phages and their bacterial hosts (Fig. 4e, f). Lyt-phages shared primarily toxin-, secretion-system-, and stress-response–related genes with bacteria, including hemolysin III (HlyIII), catalase (KatA), and type II/VI secretion system (T2SS/T6SS) effectors, consistent with their rapid-turnover and aggressive infection strategies. In contrast, Temp-phages shared a broader and more functionally complex set of genes with their hosts, encompassing adherence, anti-phagocytosis, complement protease, serum resistance, motility, capsule biosynthesis, lipopolysaccharide pathways, and regulatory elements such as PhoP/PhoR, RpoS, and Fur (Fig. 4e). For MGEs, Lyt-phages shared mainly insertion sequence (IS) elements and Tn3-family transposases, whereas Temp-phages shared RecA, RecX, XerD, integrases, and multiple recombinase families, suggesting deeper integration into bacterial evolutionary processes (Fig. 4f). Together, these findings indicate that early-life URT phages, especially Temp-phage, and their bacterial hosts participate in interconnected virulence- and mobility-associated gene networks, forming a shared functional gene pool that may enhances the ecological persistence and adaptive capacity of key URT opportunistic pathogens.

### RNA viruses reveal a novel bovine-derived betacoronavirus-like genome with evolutionary proximity to bovine and human coronaviruses

**Fig. 5:**
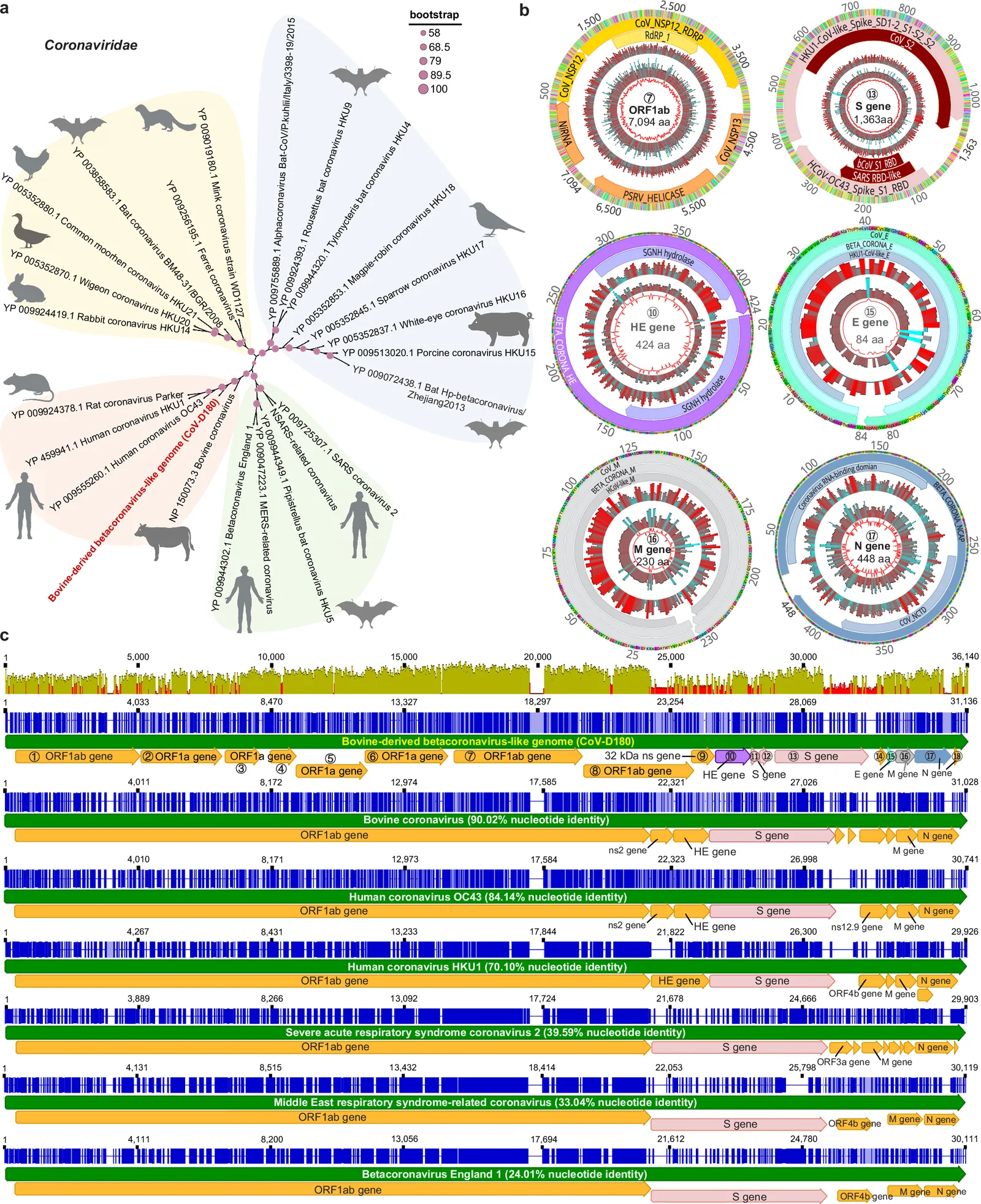
Phylogenetic and genomic characterization of a bovine-derived betacoronavirus-like genome identified in the calf URT. **a** Maximum-likelihood phylogenetic tree of the bovine-derived betacoronavirus-like genome, CoV-D180, based on the amino acid sequence of the conserved RNA-dependent RNA polymerase (RdRp). Bootstrap support values are indicated by circle size and color, and representative animal hosts are illustrated around major lineages (Animal host icons were created in BioRender. Leluo, G. (2026) https://BioRender.com/whsvu6d). **b** Circular genome annotation of predicted coding regions in CoV-D180, including open reading frame (ORF) 1ab, spike (S), hemagglutinin-esterase (HE), envelope (E), membrane (M), and nucleocapsid (N) genes. **c** Genome-wide comparison of CoV-D180 with representative betacoronavirus genomes, showing genome organization, gene synteny, and nucleotide-level similarity across homologous regions.

In the current study, we further identified an enrichment in *Coronaviridae* at Day 180, suggesting a weaning-associated increase in coronavirus. Reassembly of Day180 reads generated a 31,136-bp bovine-derived betacoronavirus-like genome (designated CoV-D180). Phylogenetic analysis based on RNA-dependent RNA polymerase (RdRp) sequences placed the reconstructed CoV-D180 genome sequence within the Embecovirus subgenus of Betacoronavirus, positioned proximal to bovine coronavirus (BCoV) and the human coronaviruses HCoV-OC43 and HCoV-HKU1 (Fig. 5a). Genome annotation revealed a typical *Embecovirus* genome organization comprising ORF1ab, HE (hemagglutinin-esterase), S (spike), E (envelope), M (membrane), and N (nucleocapsid) genes (Fig. 5b). Genome-wide nucleotide identity comparisons further revealed the CoV-D180 genome shared the highest nucleotide identity with BCoV (90.0%), followed by HCoV-OC43 (84.1%) and HCoV-HKU1 (70.1%), whereas exhibited substantially lower similarity to betacoronaviruses (SARS-CoV-2 and MERS-CoV). The overall genomic organization of CoV-D180 was conserved, with the relative positioning of the HE and S genes closely resembling that of BCoV, HCoV-OC43 and HCoV-HKU1. In contrast, sequence divergence was more concentrated in the S region relative to ORF1ab and other genomic regions (Fig. 5c). Based on genome-wide similarity, it indicates that CoV-D180 represents an *Embecovirus* lineage that is evolutionarily close to both bovine and human coronaviruses based on genome-wide similarity.

Most contigs assigned to *Picornaviridae* formed a distinct cluster that did not group with known human *Enterovirus*/*Parechovirus* clades. A subset of these contigs branched proximal to the *Aphthovirus* clade, whose members are predominantly associated with ruminants, including cattle and goats (Supplementary Fig. 6a). Similarly, contigs classified as *Astroviridae* clustered within the *Mamastrovirus* lineage and showed close phylogenetic proximity to porcine and bovine astroviruses, without direct association with known human astroviruses (Supplementary Fig. 6b). In contrast, calf-derived contigs within the *Picobirnaviridae* clustered closely with reference bovine, human, and primate picobirnaviruses, suggesting limited host segregation at the RdRp level (Supplementary Fig. 6c). Similarly, members of the *Coronaviridae* were predominantly assigned to the *Betacoronavirus* genus and exhibited close phylogenetic proximity to both bovine and human coronaviruses (Supplementary Fig. 6d).

### DNA virus families reveal a novel bovine-derived adenovirus-like genome positioned between bovine and human adenoviruses

**Fig. 6:**
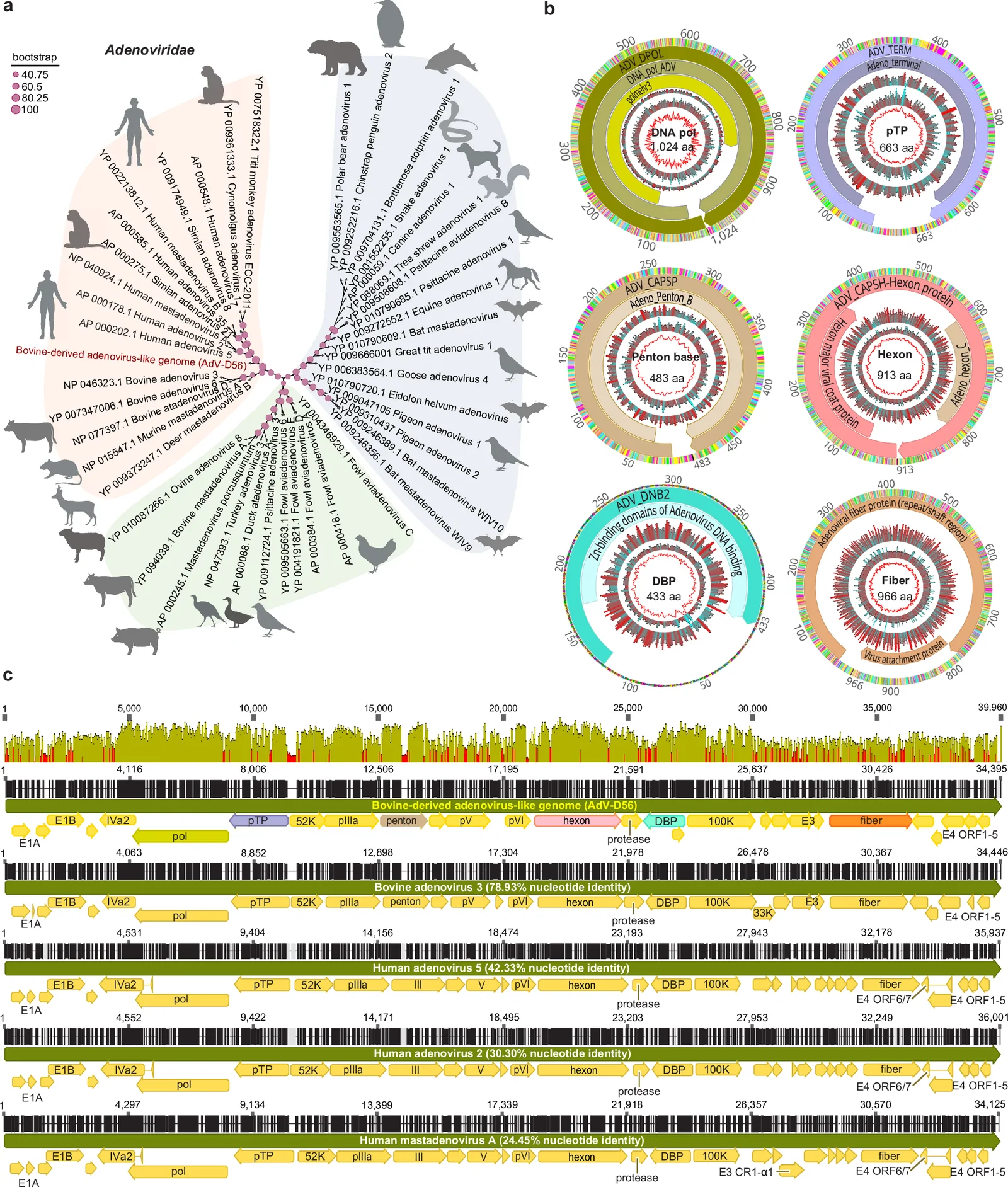
Phylogenetic and genomic characterization of a bovine-derived adenovirus-like genome identified in the calf URT. **a** Maximum-likelihood phylogenetic tree of the bovine-derived adenovirus-like genome, AdV-D56, based on the amino acid sequence of the conserved hexon protein. Bootstrap support values are indicated by circle size and color, and representative animal hosts are illustrated around major lineages (Animal host icons were created in BioRender. Leluo, G. (2026) https://BioRender.com/whsvu6d). **b** Circular genome annotation of predicted coding regions in AdV-D56, including early region 1A (E1A), early region 1B (E1B), precursor terminal protein (pTP), DNA polymerase (pol), penton base, hexon, DNA-binding protein (DBP), and fiber genes. **c** Genome-wide comparison of AdV-D56 with representative adenovirus genomes, showing genome organization, gene synteny, and nucleotide-level similarity across homologous regions.

Different from the RNA virus family *Coronaviridae*, *Adenoviridae* showed marked enrichment at the spring turnout (Day 56). De novo reassembly of reads from the Day 56 samples generated a 34,383-bp bovine-derived adenovirus-like genome, hereafter referred to as AdV-D56. Phylogenetic analysis based on the genome sequence placed AdV-D56 within the genus *Mastadenovirus*, grouping proximal to bovine adenoviruses, showing moderate sequence similarity to human adenovirus type 5 (HAdV-5), and more distant relatedness to other human adenoviruses (Fig. 6a). Genome annotation showed that AdV-D56 exhibited a canonical *Mastadenovirus* genome organization, containing key replication-associated genes (DNA polymerase (pol), precursor terminal protein (pTP), DNA-binding protein (DBP)) and major structural genes (penton base, hexon, and fiber) (Fig. 6b). Alignment-based genome-wide nucleotide identity comparisons showed the highest similarity to bovine adenovirus 3 (BAdV-3; 78.9%), followed by moderate similarity to HAdV-5 (42.3%) and lower similarity to HAdV-2 (30.3%) and HAdV-A (24.5%). Consistent with the genome-wide alignment profile, higher sequence conservation was observed across replication-associated regions (e.g., pol and pTP), whereas reduced nucleotide identity was concentrated in capsid- and attachment-associated regions, particularly hexon and fiber (Fig. 6c). These results indicate that AdV-D56 represents a bovine-associated *Mastadenovirus* lineage with conserved replication-associated genomic organization and limited genome-wide similarity to human adenoviruses.

Generally, DNA viral families of the calf URT revealed distinct patterns of host association across viral families (Supplementary Fig. 7). Among these, *Adenoviridae*, *Polyomaviridae* and *Orthoherpesviridae* exhibited close phylogenetic proximity to both ruminant- and human-associated viruses (Supplementary Fig. 7a, b, d), whereas *Parvoviridae* and *Circoviridae* showed no clear clustering with known human viruses (Supplementary Fig. 7c, e). Within the *Polyomaviridae*, calf-derived contigs were distributed across both the *Epsilonpolyomavirus* and *Betapolyomavirus* lineages, clustering closely with ruminant-associated viruses such as *Epsilonpolyomavirus bovis* and Capra aegagrus polyomavirus, as well as the human- or primate-associated *Betapolyomavirus tertihominis*. Similarly, within the *Orthoherpesviridae*, several calf-derived contigs clustered within the *Gammaherpesvirinae* subfamily, forming lineages adjacent to bovine gammaherpesvirus 4 and human gammaherpesvirus 8. In contrast, calf-derived DNA viruses within the *Parvoviridae* and *Circoviridae* families did not show close phylogenetic relationships with known human viruses. *Parvoviridae* contigs were distributed across both *Parvovirus* and *Chapparvovirus* lineages, clustering primarily with porcine, rodent, or primate-associated viruses. Likewise, *Circoviridae* contigs were positioned within the *Cyclovirus* genus and clustered with bovine-, bat-, and flying-fox-associated cycloviruses, indicating a predominantly animal-associated viral spectrum.

### CoV-D180- and AdV-D56-specific reads were identified in human respiratory metatranscriptomic datasets

**Fig. 7:**
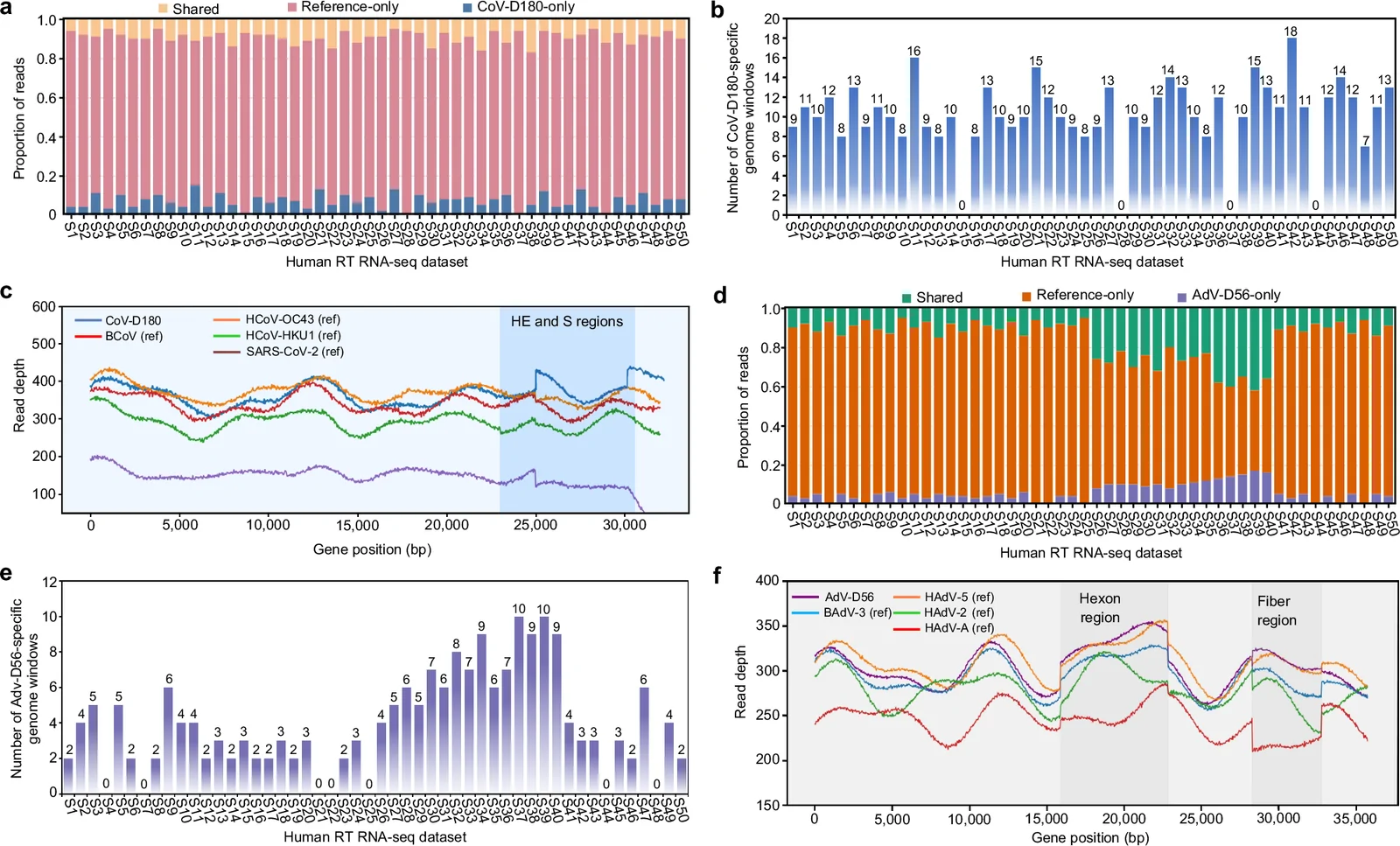
Comparative mapping patterns of bovine-derived CoV-D180 and AdV-D56 in human respiratory tract metatranscriptomic datasets. **a** Proportions of coronavirus-associated reads classified as shared between CoV-D180 and reference coronavirus genomes, including bovine coronavirus (BCoV), human coronavirus OC43 (HCoV-OC43), human coronavirus HKU1 (HCoV-HKU1), and severe acute respiratory syndrome coronavirus 2 (SARS-CoV-2), reference-only, or CoV-D180-only across individual human RT RNA-seq datasets. **b** Number of CoV-D180-specific genomic windows identified in each dataset using non-overlapping 500-bp sliding windows. A window was defined as CoV-D180-specific if it contained at least two CoV-D180-only reads with a mapping quality score (MAPQ) ≥ 20 and no reads mapping to any reference coronavirus genome under the competitive mapping framework. **c** Representative genome-wide smoothed read-depth profiles using a 50-bp rolling mean for CoV-D180 and reference coronaviruses derived from competitive mapping analysis. **d** Proportions of adenovirus-associated reads classified as shared between AdV-D56 and reference adenovirus genomes, including bovine adenovirus type 3 (BAdV-3), human adenovirus type 5 (HAdV-5), human adenovirus type 2 (HAdV-2), and human adenovirus species A (HAdV-A), reference-only, or AdV-D56-only across individual human RT RNA-seq datasets. **e** Number of AdV-D56-specific genomic windows identified in each dataset using the same non-overlapping 500-bp window criteria as in panel (**b**). **f** Representative genome-wide smoothed read-depth profiles using a 50-bp rolling mean for AdV-D56 and reference adenoviruses derived from competitive mapping analysis.

To assess the One Health relevance of the newly identified bovine-derived viral genomes (CoV-D180 and AdV-D56), a competitive mapping of publicly available human respiratory metatranscriptomic datasets was performed against these novel genomes alongside representative coronavirus and adenovirus reference genomes (Fig. 7, Supplementary Data 32 and 33). Across datasets, most coronavirus-associated reads mapped to established reference coronaviruses, while a consistent subset of reads mapped specifically to CoV-D180 under this reference set, accounting for 4.27–17.25% of coronavirus-associated reads across datasets (Fig. 7a). Sliding-window analyses further identified multiple CoV-D180-specific genomic windows in most datasets, ranging from 2–18 windows per dataset (Fig. 7b). Genome-wide depth profiles revealed structured coverage along the CoV-D180 genome, with profiles partially overlapping yet clearly distinguishable from those of BCoV and human coronavirus references (HCoV-OC43 and HCoV-HKU1), with the most apparent differences observed across the HE and S regions (Fig. 7c). Using the same analytical framework, mapping to the bovine-derived adenovirus genome AdV-D56 showed more constrained read-mapping patterns in human respiratory datasets: AdV-D56 only reads accounted for 1.24–16.35% of adenovirus-associated reads across datasets (Fig. 7d), and the number of AdV-D56-specific genome windows was consistently fewer, ranging from 2–10 per dataset (Fig. 7e). Depth profiling of AdV-D56 revealed structured genome-wide coverage, with overall profiles broadly comparable to those of adenovirus references, while displaying localized differences in coverage intensity and pattern across specific genomic regions, including the hexon- and fiber-associated loci (Fig. 7f). Taken together, these analyses reveal detectable, regionally structured cross-host sequence similarity signatures for the newly identified bovine-derived viral genomes in human respiratory datasets, characterized by coverage patterns distinct from those of reference viruses.

## Discussion

The current study is one of the first efforts to characterize the early-life RT bacteriome and virome, including both prokaryotic and eukaryotic viruses, in beef calves and to determine their ecological establishment and succession within animal and microbial hosts, including cross-host associations at the human-animal interface. Given the low-biomass nature of DNS samples, we optimized an integrated omics workflow by including viral particle enrichment prior to nucleic acid extraction, followed by shotgun sequencing of both viral DNA and RNA, metagenomic profiling of bacterial communities, together with NCs-based quality assessment. Compared with previous URT virome studies^17,23^, the incorporation of viral particle enrichment enabled the concurrent recovery of viral DNA and RNA, improving detection sensitivity in low-biomass samples. Coupled with this experimental optimization, it has allowed the separation of phages and vertebrate-associated viruses into independent analytical branches, improved ecological interpretability and minimized classification bias. Particularly given that current viral quality assessment tools such as CheckV are primarily benchmarked on complete dsDNA viral genomes, which may underrepresent fragmented or ssRNA viruses^21,22,24,25^. Notably, appropriate NC samples are essential for microbiome analyses of low-biomass samples, as background noise or contaminants may be introduced at multiple experimental steps, including nucleic acid extraction, amplification, library preparation, and sequencing. In this study, background microbial signatures were detected across NCs representing different stages of the workflow, with relatively more evident features in amplification-related controls, particularly whole-transcriptome and whole-genome amplification. This suggests that amplification may be a key source of background microbial signatures in low-biomass samples. Due to the lack of well-established and validated criteria for interpreting NC-associated microbial signatures, we applied branch-specific evaluation strategies for bacteria, phages, and vertebrate-associated viruses to distinguish potential technical background from biologically relevant signatures and to minimize both false-positive and false-negative interpretations. These observations indicate that NC-detected microbial signatures in RT samples should not be interpreted using a simple presence/absence rule. Instead, microbial signatures shared between DNS and NC samples should be interpreted cautiously by considering contig-level patterns, such as contig representation and alignment quality, together with additional supporting evidence. These findings highlight the importance of including stage-specific NCs, especially amplification-related controls, in future low-biomass microbiome and virome studies. Together, this optimized experimental and analytical framework provides a foundation for resolving distinct assembly mechanisms and evolutionary signatures within the early-life calf URT microbiome.

Evidence from human gut studies has shown that phage-bacteria interactions are established early in life and play a key role in shaping microbial community assembly^26,27^. Here, we observed that, unlike gut phagome, phages were rare in the calf URT during the first week of life but expanded rapidly thereafter, closely paralleling the establishment of bacterial communities. Assembly analyses further indicated that URT phage communities were predominantly shaped by deterministic processes and showed strong temporal synchronization with their bacterial hosts. Similar host-driven restructuring of phage communities has been reported in other microbial ecosystems, where bacterial turnover alters infection networks and phage composition^28–30^. However, bacterial communities in the calf URT occupied an intermediate position along the stochastic-deterministic continuum, consistent with rapid early-life respiratory microbiome succession influenced by environmental exposure and host immune maturation^31–34^. Differences in phage lifestyle further contributed to the observed phage-host dynamics. Unlike the rumen phageome^35^, the calf URT virome was dominated by Temp- phages, likely reflecting ecological constraints of the URT, including low and fluctuating bacterial densities, limited viral diffusion, and a spatially structured, mucosa-associated environment. Such conditions favor lysogeny as a persistence strategy under frequent host stress, consistent with the Piggyback-the-Winner model^36^. In contrast, Lyt-phages, which are typically favored in fluid or nutrient-rich environments, exhibited delayed increases in abundance relative to their bacterial hosts, consistent with predator-prey dynamics described for virulent phage–bacteria systems^37,38^. Notably, Temp-phages in the URT were predominantly associated with bacterial taxa that include well-recognized RT opportunistic or zoonotic-associated species, indicating that bacterial populations of potential One Health relevance can be established in the URT of healthy calves and may serve as a baseline for understanding microbiota shifts under altered selective pressures, such as disease or antimicrobial exposure.

Temp-phages in the calf URT exhibited pronounced functional convergence with their bacterial hosts, as reflected by shared virulence- and mobility-associated genes. Adherence- and anti-phagocytosis-related functions were persistently detected in both Temp-phages and their hosts across developmental stages. These traits have been shown to facilitate epithelial attachment, biofilm stability, and sustained colonization during the establishment of resident pathobiont communities^39–41^. In addition, Temp-phages shared a diverse repertoire of mobility-related gene families with their hosts, including integrases, recombinases, and transposases, which together constitute the molecular machinery underlying genomic integration, recombination, and mobilization of adaptive loci. These features suggest that prophages may function as long-term reservoirs and mediators of gene flow, thereby contributing to functional innovation and adaptive flexibility within the URT ecosystem^42–44^. In contrast, Lyt-phages displayed a narrower host-interaction signature dominated by stress-response and toxin-associated genes and carried fewer mobile genetic elements, consistent with rapid infection cycles and a more transient role in host population restructuring^45–48^. Collectively, these contrasting functional profiles reinforce the distinct ecological contributions of temperate and lytic phages to early-life URT microbiome organization.

The identification of vertebrate-associated viruses in the calf URT supports the concept that cattle represent an important host at the human-animal interface for RT microbial communities. Vertebrate-associated viruses in the calf URT predominantly emerged after the first week of life and accumulated during subsequent developmental stages, exhibiting largely stochastic and exposure-driven assembly patterns. Such exposure-driven processes may introduce substantial stochasticity into viral community assembly^49^. Following pasture exposure and weaning, we observed stage-specific enrichment of *Adenoviridae* and *Coronaviridae*. These stage-specific enrichments suggest that increased environmental exposure and weaning-associated stress may enhance susceptibility to certain RT viral infections^16,50^. Notably, near-complete bovine-derived coronavirus-like and adenovirus-like genomes recovered in this study displayed conserved genome organization and replication-associated modules, alongside increased divergence in host-interaction and surface-associated genes relative to known bovine- and human-associated viruses. This genomic architecture is consistent with host-specific adaptation, in which evolutionary stability of core replication functions coexists with diversification of host-interaction regions^51–53^. Competitive read-level mapping of human respiratory metatranscriptomic datasets to bovine-derived CoV-D180 and AdV-D56 revealed genome-specific and regionally structured coverage patterns that were distinct from those of reference bovine and human viruses. These patterns included discrete genomic windows uniquely recruited by the bovine-derived genomes under competitive mapping conditions, indicating that the observed cross-host mapping is not driven solely by generic sequence conservation among respiratory viruses^54^. Notably, reference coronaviruses and adenoviruses did not exhibit comparable coverage profiles when aligned to the same datasets, suggesting that these signatures are specific to the newly characterized bovine-derived genomes rather than a general feature of known bovine respiratory viruses. Importantly, these findings do not indicate active replication or infection in human hosts^55^; instead, they support fragment-level evolutionary continuity in which conserved replication-associated frameworks coexist with diversification in host-interaction and surface-associated regions^56^, consistent with host-specific adaptation under distinct ecological and selective pressures.

**Fig. 8:**
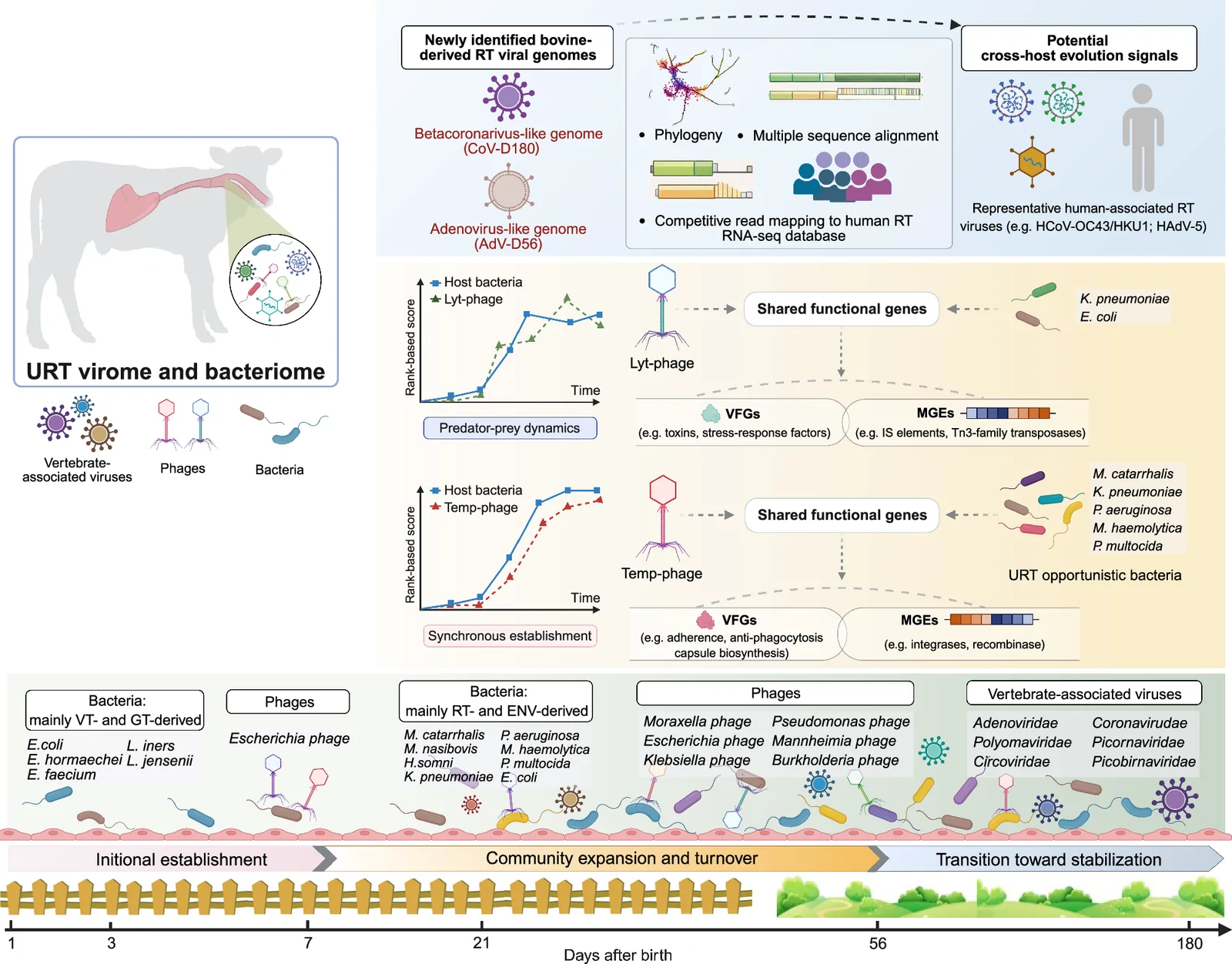
Early-life bovine URT virome dynamics featuring phage-opportunistic bacteria interactions and potential cross-host evolutionary relatedness. Early-life establishment of the calf URT virome and bacteriome is depicted from birth to Day 180. Early colonization is characterized by vaginal tract (VT)- and gastrointestinal tract (GT)-associated bacteria and Escherichia-associated phages, followed by community expansion and turnover involving respiratory tract (RT)- and environment (ENV)-associated bacteria, diversified phage populations, and vertebrate-associated viruses. Temp-phages show synchronous temporal patterns with predicted bacterial hosts and share VFGs and MGEs, whereas Lyt-phages show predator–prey-like dynamics with host bacteria. Newly identified bovine-derived betacoronavirus-like genome CoV-D180 and adenovirus-like genome AdV-D56 were characterized by phylogenetic analysis, multiple sequence alignment, and competitive read mapping against human respiratory tract RNA-seq datasets, indicating lineage-level evolutionary relatedness and genome regions distinct from currently characterized reference viruses. (Created in BioRender. Leluo, G. (2026) https://BioRender.com/dv52k93).

In conclusion, the URT phages of neonatal beef calves exhibited host-associated assembly patterns and lifestyle-dependent temporal dynamics, highlighting their potential role in shaping microbial community structure during early development. In addition, newly identified bovine-derived coronavirus-like and adenovirus-like genomes exhibit conserved replication-associated architectures alongside lineage-specific divergence in host-interaction regions and show fragment-level, region-specific sequence similarity to human RT metatranscriptomes, supporting evolutionary continuity across respiratory viruses from different host species. Although this study provides an overview of the establishment and ecological succession of the URT virome in beef calves, the host prediction for both phages and animal viruses relied primarily on in silico approaches, which, while informative, cannot fully resolve in vivo host range or infection dynamics, highlighting the need for experimental validation such as virus isolation or host-range assays. Additionally, although cross-host sequence similarity was observed between bovine- and human-associated viral lineages, future studies integrating serological evidence, tissue tropism analyses, and receptor-binding assays, ideally in larger cohorts and with multi-level functional approaches, will be required to clarify their ecological context and evolutionary significance. Regardless, the findings from this study improve our understanding of bovine URT virome establishment during early life and provide evidence supporting further evaluation of cross-host evolutionary signatures of viral pathogens between humans and cattle within a One Health framework Fig. 8.

## Methods

### Animal trial design and sampling

This study was conducted on ten newborn beef calves (Bos taurus; Angus × Hereford cross), comprising six males and four females, from a research herd maintained at the Animal Diseases Research Institute (ADRI) in Lethbridge, Alberta, Canada. Both sexes were included in the study design. The effect of sex on virome and bacteriome community composition was assessed using PCoA, permutational multivariate analysis of variance (PERMANOVA), and PERMDISP. No significant sex-associated differences in community composition or multivariate dispersion were detected in either dataset. Sex-disaggregated sample information, PCoA coordinates, and statistical results are provided in the Source Data file. Ethical approval was granted by the Animal Care Committee of the Canadian Food Inspection Agency, and all procedures were performed in accordance with the guidelines of the Canadian Council on Animal Care (CCAC, 2009). The ADRI herd is managed under a long-term biosecurity and health surveillance program and is free from major bovine respiratory and enteric pathogens, including *Brucella abortus*, *Leptospira spp*., bovine viral diarrhea virus, and bovine herpesvirus-1. All calves enrolled in the study were raised in a pasture-based research herd, remained clinically healthy throughout the study period, and had not received any vaccinations or antibiotic treatments prior to or during sampling. From birth (Day 1) to Day 50, calves were housed with their dams in individual outdoor pens under natural environmental conditions, with *ad libitum* access to water and a silage-hay diet. After Day 50, calves were moved to pasture-based grazing and remained under grazing conditions until weaning at Day 180. Deep nasopharyngeal swab samples were collected longitudinally from each calf at six time points corresponding to Days 1, 3, 7, 21, 56, and 180 after birth to characterize the establishment and temporal dynamics of the upper respiratory tract microbiome.

The sampling procedure followed a guarded nasopharyngeal collection method. Briefly, the external surface of the left nostril was gently wiped with a tissue moistened with 70% ethanol to remove visible debris. A sterile guarded rayon-tipped swab (27 cm; MW124, Medical Wire & Equipment, Corsham, UK) was then inserted through the nostril into the nasopharynx, where the tip was extended beyond the guard and rotated gently against the mucosal surface before being retracted and removed; the second swab was collected in parallel. Swab tips were immediately placed into sterile, pre-labeled tubes containing liquid Amies medium to maintain microbial viability and structural integrity. Swabs designated for bacterial and viral workflows were placed into a mixture of 20% glycerol and 80% brain-heart infusion broth, or into RNAlater solution containing 20 mM EDTA, 25 mM sodium citrate, and 4.1 M ammonium sulfate (pH ≈ 5.2), respectively. All samples were kept on ice immediately after collection, transported to the laboratory within 1 h, and stored at −80 °C until further processing.

### Viral and bacterial nucleic acid extraction, amplification and sequencing

For each DNS sample, 200 µL of nuclease-free water was added to the tube containing the swab, followed by vortexing for 30 s to elute microbial particles. The tube was then centrifuged at 1500 × *g* for 5 min at 4 °C, and the supernatant was transferred to a 0.45 µm filter column (Spin-X centrifuge tube filter, Sigma-Aldrich). This filtration step was designed to enrich viral particles by removing bacterial cells and larger debris. The filtrate and filter were then separated by centrifugation at 16,000 × *g* for 1 min. The filtrate containing virus-enriched particles was collected for viral DNA/RNA extraction, while the filter membrane retaining bacterial cells was used for bacterial DNA extraction. Total DNA was extracted using the GeneJET Genomic DNA Purification Kit (Thermo Fisher Scientific, USA),^57^ for bacterial profiling. Viral nucleic acids were extracted using the NucleoSpin Virus Kit (Macherey-Nagel, Düren, Germany) according to the manufacturer’s protocol. In brief, 200 µL of lysate was mixed with Proteinase K and carrier RNA to promote viral particle lysis and nucleic acid binding. After ethanol addition, the mixture was loaded onto a NucleoSpin Virus Column and centrifuged to allow nucleic acids to bind to the silica membrane. The column was sequentially washed with Buffers VW1 and VW2, followed by a final high-speed centrifugation to remove residual contaminants. Viral DNA and RNA were eluted with 30 µL of preheated RNase-free water and stored at −80 °C until further processing. To obtain sufficient viral templates for sequencing, both viral DNA and RNA were amplified using the REPLI-g Cell WGA & WTA Kit (QIAGEN, Hilden, Germany). RNA templates were first reverse transcribed into cDNA using the Quantiscript reverse transcription system (QIAGEN, Hilden, Germany) at 42 °C for 60 min, followed by heat inactivation at 95 °C for 3 min. The resulting cDNA and viral DNA were then subjected to ligation at 24 °C for 30 min, and isothermal amplification was carried out at 42 °C for 60 min using the REPLI-g SensiPhi polymerase system. The reaction was terminated by heating at 95 °C for 3 min, and amplified products were stored at −20 °C until library preparation and sequencing. Metagenomic libraries were constructed from amplified viral nucleic acids using the NEBNext® Ultra II DNA Library Prep Kit (New England BioLabs, Ipswich, MA, USA), following a protocol suitable for low-input DNA (≥500 pg). The resulting libraries were subjected to paired-end sequencing (2 × 150 bp) on the Illumina NovaSeq platform (Illumina, San Diego, CA, USA). Negative controls were included throughout the experimental workflow to monitor background microbial signatures introduced during sample processing, nucleic acid extraction, amplification, library preparation, and sequencing. These controls included whole-transcriptome amplification (nc-wta), whole-genome amplification (nc-wga), an environmental (bench) swab control (nc-swab), nucleic acid extraction controls processed with or without carrier RNA supplied by the extraction kit (nc-with-carrier and nc-no-carrier), and a library preparation PCR control (nc-library). All NCs samples were processed alongside the DNS samples and sequenced using the same platform.

### Quality control and assembly of metagenomic sequencing data

Raw paired-end sequencing reads from DNS samples were processed using Trimmomatic (v0.39) to remove adapter sequences and low-quality bases, with the parameters “ILLUMINACLIP: adapters. fa: 2:30:10, SLIDINGWINDOW:4:15, and MINLEN:75”^58^. The quality of the clean reads was assessed using FastQC (v0.12.1)^59^. Host-derived reads were removed by aligning the quality-filtered reads to the bovine reference genome (GCA_002263795.4, ARS-UCD2.0; https://ftp.ncbi.nlm.nih.gov/genomes/all/GCA/002/263/795/GCA_002263795.4_ARS-UCD2.0/) using BWA (v0.7.18) with the bwa mem algorithm and parameters “-t 20 -M”^60^. The aligned reads corresponding to the host genome were then removed using an in-house Perl script (remove-host.pl). Unmapped (non-host) reads were retained for downstream metagenomic analyses. Quality-filtered reads were subsequently assembled de novo using MEGAHIT (v1.2.9) with the parameter --min-contig-len 500^61^.

### Phages identification and host prediction

From the assembled contigs, sequences ≥1 kb were selected for viral identification using four complementary approaches: 1) BLASTn searches were performed against the Integrated Microbial Genomes/Viral Resource (IMG/VR, v4.0) database^62^, with viral hits defined by an E-value ≤ 1e-5. 2) VirSorter2 (v2.0) was applied with default parameters to identify viral contigs across multiple viral groups (dsDNA phages, NCLDVs, RNA, and ssDNA viruses), retaining sequences with a classifier score > 0.7^63^. 3) VIBRANT (v1.2.1) was run with default settings to identify viral sequences, infer viral lifestyle (lytic or lysogenic), and assess genome quality (complete, high-, medium-, or low-quality) based on hallmark gene content and genome structure^64^. Gene annotation was performed using HMMER3 with a bit score threshold ≥ 30. 4) DeepVirFinder (v1.1) was used to predict viral sequences from contigs ≥ 1 kb, with VirFinder scores ≥ 0.7 and *q*-values ≤ 0.05 considered significant. Contigs with *q*-values < 0.01 were retained as high-confidence viral candidates^65^. The candidate viral contigs identified by at least one viral detection tool using any reference database were further assessed using CheckV (v0.7.0)^21^. NCs-derived contigs were processed using the same viral identification and CheckV-based quality assessment workflow as DNS-derived contigs. DNS sample- and NCs sample-derived contigs classified as low-quality and not-determined were excluded from downstream bacteriophage analyses. Viral operational taxonomic units (vOTUs) were clustered using PhageAnnotator (https://github.com/nf-core/phageannotator) based on sequence similarity, with a threshold of > 95% nucleotide identity and ≥ 85% alignment coverage of the total sequence length^66^. The longest contig within each cluster was selected as the representative vOTU. Novelty of vOTUs was assessed by sequence comparison against the IMG/VR database (v4.0). Representative contigs from each vOTU cluster were searched using BLASTn with default parameters. vOTUs were searched against the IMG/VR database using BLASTn. A vOTU was classified as known when the top hit exhibited ≥ 90% nucleotide identity and ≥75% alignment (query) coverage. vOTUs that did not simultaneously meet both criteria were classified as uncharacterized^67^. Taxonomic classification of viral genomes was performed using VITAP through a standardized workflow^68^. First, the latest viral reference metadata (VMR_MSL40.v1.20250307.xlsx) was obtained from the International Committee on Taxonomy of Viruses (ICTV; https://ictv.global/vmr) and processed using the command VITAP upd --vmr./ICTV_VMR_MSL40/ICTV_VMR-MSL40.csv -o./ICTV_VMR/ICTV_VMR.csv -d VMR-MSL to generate an indexed reference database. Subsequently, viral sequences (virus.fa) were taxonomically classified via homology-based analysis using VITAP assignment -i virus.fa -d./DB_VMR-MSL/ -o test_result, which leverages ICTV-curated classifications to ensure standardized and up-to-date viral annotations^68^. Host prediction and lifestyle classification of phage vOTUs were performed using PhaBOX (v2.1.7)^69^ with default parameters.

### Bacterial identification and read-count profiling

Bacterial contigs >1 kb were subjected to taxonomic annotation using DIAMOND BLASTx (v2.9.0+) against the NCBI NR protein database downloaded in September 2025. To ensure consistent filtering between biological samples and NCs, the same annotation criteria were applied to DNS- and NC-derived contigs. Only matches with an alignment length > 50, identity > 50%, query coverage > 70%, and E-value < 1E-10 were retained for downstream bacterial classification. Taxonomic assignments were inferred from the best-scoring hits and further refined using the lowest common ancestor (LCA) algorithm implemented in MEGAN6, allowing for a more conservative and accurate classification across hierarchical levels^70^. For taxa detected in both DNS and NCs samples, potential background microbial signatures were assessed based on NC type, contig number, alignment length, identity, and query coverage. To generate bacterial read-count profiles, high-quality reads were aligned to the annotated bacterial contigs using BWA-MEM (v0.7.18) with the parameters “-t 20 -M”. The number of mapped reads per contig was extracted from the resulting BAM files using SAMtools (v1.22.1)^71^. Subsequent abundance-sensitive statistical analyses and visualizations were performed using rank-order profiles derived from raw read counts, as described in the Statistical analysis section. Ecological and host-associated metadata for identified bacterial taxa, including typical host range and environmental origin, were retrieved from the BacDive database (The Bacterial Diversity Metadatabase; https://bacdive.dsmz.de), accessed in September 2024, to support downstream ecological interpretation.

### Virulence factor and mobile genetic element profiling in phages and bacteria

To characterize the functional potential of phages and bacteria, protein-coding sequences were annotated for VFGs and MGEs. VFs were identified using DIAMOND BLASTp (v2.14.1) against the VF database (http://www.mgc.ac.cn/VFs/) with thresholds of E-value < 1E-5, sequence identity ≥ 70%, and alignment coverage ≥ 70%. MGEs were identified using DIAMOND BLASTp (v2.14.1) against the MGEs90 database (accessed on 2025-09-15) under the same parameter settings. To identify shared functional genes between phages and bacteria, annotated VF and MGE genes were compared across phage and bacterial contigs. Protein sequences were aligned using DIAMOND BLASTp (v2.14.1) with a minimum sequence identity of ≥ 70%, alignment coverage ≥ 70%, and E-value < 1E-5. Genes detected in both phage and bacterial contigs with these homology criteria were defined as shared genes. The co-occurrence relationships among shared functional genes were visualized using network analysis in Cytoscape (v3.9.1), where nodes represent gene categories and edges represent shared occurrences between phages and bacteria.

### Vetebrate-associated virus identification and reassembly

Viral contigs >1 kb were taxonomically identified using BLASTx (v2.9.0+) against the viral subset of the NCBI NR-viral (downloaded in 2025-07). The resulting hits with an E-value ≤ 1E-10 and identity (%) >50 were retained as candidate viral sequences. To further validate the taxonomic assignments, these contigs were compared to the viral subset of the NCBI nucleotide database (NT-viral) using BLASTN (v2.9.0+) with the same significance threshold. Contigs showing consistent viral hits in both NR- and NT-based alignments were classified as high-confidence viral sequences. To evaluate potential NCs-derived background viral signatures in the vertebrate-associated virus branch, NCs-derived contigs were annotated against the same NR-viral and NT-viral databases using the same filtering criteria as DNS-derived contigs. Viruses shared between DNS and NCs samples were further assessed using contig-level metrics, including contig coverage (%), database coverage (%), sequence identity (%), and contig length. Shared viruses with comparable or stronger support in NCs were annotated as NCs-associated viral signatures, while viruses with significantly stronger contig-level support in DNS samples were retained as DNS-dominant viruses. Taxonomic classification was inferred from retained high-scoring hits and further verified using the LCA algorithm implemented in MEGAN6^24,25^. Viral genome types and host information were annotated according to the ICTV taxonomy database (https://ictv.global/taxonomy) and the Viral-Host database (https://viralhostdb.Bioca ddie.org/). To obtain higher-quality viral genome sequences with improved sequence continuity, targeted reassembly was performed using sequencing reads from the Day 180 samples for coronavirus and from the Day 56 samples for adenovirus. Prior to reassembly, host-derived sequences were removed, and assembly was conducted using SPAdes (v3.15.5) with the parameters “-t 48 -m 150 --meta --disable-gzip-output”. To further extend the genome ends and improve sequence continuity, iterative assemblies were performed using the SPAdes options “--untrusted-contigs and --trusted-contigs”^72^.

### Phylogenetic analysis, genome annotation and multiple sequence alignment of vertebrate-associated viruses

To elucidate the evolutionary relationships of the vertebrate-associated viruses, phylogenetic analyses were performed separately for RNA and DNA viruses. For RNA viruses, evolutionary inference was based on the amino acid sequences of the RdRp, whereas for DNA viruses, conserved marker proteins characteristic of each viral family was used^24^. The RdRp and family-specific conserved proteins were identified through BLASTp homology searches and Pfam domain annotation (Supplementary Data 34–37). Open reading frames (ORFs) were predicted from viral contigs using Prodigal (v2.6.3) in metagenomic mode (-p meta)^73^. Functional annotation of predicted viral proteins was performed using the InterProScan web service with default parameters. Annotated viral genomes were imported into Geneious Prime (v2025.2.2) for genome organization visualization and manual inspection of gene boundaries. Multiple sequence alignments were performed using MAFFT (v7.525) with the --auto setting. Poorly aligned and gapped regions were trimmed using TrimAl (v1.5) with the -gappyout parameter. Maximum likelihood phylogenetic analyses were conducted using IQ-TREE (v2.3.5), with the optimal substitution model automatically selected (-m MFP). Resulting phylogenetic trees were visualized and annotated using iTOL (Interactive Tree of Life)^74^. Representative reference genomes used for phylogenetic comparison were retrieved from the NCBI GenBank database (accessed on 2025-9-15).

### Cross-host competitive mapping of virus-associated reads in human respiratory metatranscriptomic datasets

Publicly available human respiratory RNA-seq datasets were retrieved from the NCBI Sequence Read Archive (SRA) using the SRA Toolkit (v3.0.0; prefetch and fasterq-dump). Raw sequencing reads were initially assessed using FastQC (v0.12.1), and summary quality reports were generated with MultiQC (v1.14)^75^. Adapter sequences and low-quality bases were removed using Trimmomatic (v0.39). Reads were processed in paired-end mode with Illumina adapter trimming, removal of low-quality leading and trailing bases (LEADING:3, TRAILING:3), sliding-window quality trimming (SLIDINGWINDOW:4:15), and minimum length filtering (MINLEN:50). Quality-filtered reads were re-evaluated using FastQC prior to downstream analyses.

Newly identified bovine-derived viral genomes, including CoV-D180 and AdV-D56, were analyzed together with representative reference genomes. For coronavirus analyses, reference genomes included bovine coronavirus (BCoV; NC_003045.1), human coronavirus OC43 (HCoV-OC43; NC_006213.1), human coronavirus HKU1 (HCoV-HKU1; NC_006577.2), and SARS-CoV-2 (NC_045512.2). For adenovirus analyses, reference genomes included bovine adenovirus 3 (BAdV-3), human adenovirus 5 (HAdV-5), human adenovirus 2 (HAdV-2), and human adenovirus A (HAdV-A). For each viral group, bovine-derived and reference genomes were combined into a single multi-FASTA file, and alignment indices were constructed using Bowtie2 (v2.5)^76^. Quality-controlled RNA-seq reads were aligned to the combined genome set in end-to-end mode using identical alignment parameters across all datasets, enabling competitive mapping of reads to all genomes simultaneously. Alignments were filtered using a minimum mapping quality score (MAPQ) of 20 to retain confident matches^77^. Virus-associated reads were classified into three categories based on their competitive mapping outcomes: (1) Reference-only reads, defined as reads mapping exclusively to reference genomes but not to the bovine-derived genome; (2) Bovine-derived genome-only reads, defined as reads mapping exclusively to CoV-D180 or AdV-D56 but not to any reference genomes; and (3) Shared reads, defined as reads that did not show exclusive assignment to either category under the competitive mapping framework. For each dataset, the number of reads in each category was normalized to the total number of virus-associated reads, and read proportions were calculated. Stacked bar plots summarizing read category proportions across datasets were generated using R (v4.3.1) with the ggplot2 package.

### Window-based identification of virus-specific genomic regions and genome-wide read depth profiling

To characterize the genomic distribution of bovine-derived virus-specific signatures, sliding-window analyses were performed along the CoV-D180 and AdV-D56 genomes. Each genome was partitioned into consecutive, non-overlapping 500-bp windows using custom scripts. Virus-specific reads were identified based on the competitive mapping results described above. For each dataset, genomic coordinates of bovine-derived genome-only reads (MAPQ ≥ 20) were intersected with window coordinates to determine the presence of virus-specific signatures within each window. A window was defined as virus-specific if it contained at least two reads mapping exclusively to CoV-D180 or AdV-D56 and no reads mapping to any reference genomes under the competitive mapping framework. No additional abundance threshold was applied. The total number of virus-specific genomic windows identified in each dataset was summarized.

Genome-wide read depth profiles were generated to visualize coverage patterns along viral genomes. Following competitive mapping, alignment files (BAM format) were processed using SAMtools (v1.22.1, depth)^71,78^ to calculate per-base read depth, defined as the number of sequencing reads covering each nucleotide position of the genome. For visualization purposes only, per-base read depth values were smoothed using a 50-bp rolling mean, which was applied uniformly across all genomes and datasets to reduce local stochastic fluctuations while preserving overall coverage trends. All quantitative analyses were performed using unsmoothed read depth values. Smoothed read depth profiles were plotted along genomic coordinates for CoV-D180 together with coronavirus reference genomes, and for AdV-D56 together with adenovirus reference genomes. All coverage profiles were visualized using R (v4.3.1) with the ggplot2 package.

### Statistical analysis

All statistical analyses and data visualization were performed using R software (v4.3.1) and GraphPad Prism (v9.5). To reduce potential amplification biases associated with abundance estimation in low-biomass samples, downstream community and feature-level analyses were conducted using a zero-preserving rank-based approach. Raw read-count profiles were first aggregated at the corresponding taxonomic or functional level and then transformed using a zero-preserving rank-based approach^79^. Within each sample, only detected features were ranked based on raw read counts, while undetected features were retained as zero. The resulting rank-based score matrices were then used to support taxon-level temporal comparisons, viral family- and genome type-level summaries, heatmap visualization, and phage-host temporal trend analyses. Beta-diversity dissimilarities were calculated as 1-ρ based on Spearman’s rank correlation, followed by PCoA. Group differences in β-diversity were tested using PERMANOVA (Adonis, vegan package, 999 permutations), with permutations constrained by calf identity to account for repeated sampling. Incidence-based β-diversity partitioning was performed by converting raw read-count profiles to presence-absence data. Sørensen dissimilarity (β_sor) was decomposed into turnover (β_sim) and nestedness resultant components (β_sne) using the betapart package following the Baselga framework^80^. Ternary plots were generated to describe the relative contributions of turnover, nestedness, and Sørensen similarity (1-β_sor) among samples^81^. Alpha diversity was evaluated using effective Shannon diversity^82^ and Pielou’s evenness calculated from raw read-count profiles. Given the repeated-measures design, differences across time points were assessed using Friedman tests followed by Dunn’s multiple-comparison tests, with adjusted *P* values reported for pairwise comparisons. For feature-level temporal analyses, Friedman tests were used to test overall time-point effects, and Benjamini-Hochberg FDR correction was applied across tested features. For comparisons between DNS samples and NCs, contig-level alignment and coverage metrics, including alignment length, sequence identity, coverage, contig coverage, database coverage, and contig length, were compared using two-sided Wilcoxon rank-sum tests, with FDR correction applied for multiple comparisons. Community assembly processes were further evaluated using a Raup-Crick null model framework^83^. Presence-absence-based Raup-Crick dissimilarities were calculated to assess deviations from random community assembly, with values approaching −1 indicating deterministic assembly and values near 0 reflecting stochastic assembly. Raup-Crick analyses were performed separately for bacterial, bacteriophage, and vertebrate-associated viral communities across developmental stages. For Raup-Crick-based categorical comparisons, sample pairs were classified into deterministic or stochastic categories based on established Raup-Crick distance thresholds. Chi-square tests were used to test whether the proportions of deterministic and stochastic assembly differed within each community group or sampling time point.

### Reporting summary

Further information on research design is available in the Nature Portfolio Reporting Summary linked to this article.

## Supplementary information

Supplementary Information

Description of Additional Supplementary Files

Supplementary Code and Software

Supplementary Data 1-37

Reporting Summary

Transparent Peer Review file

## Source data

Source Data

## Data Availability

The raw sequencing data generated from deep nasopharyngeal swab metagenomic libraries of beef calves have been deposited in the NCBI Sequence Read Archive (SRA) under BioProject accession numbers PRJNA1259590 and PRJNA1259072. The viral genome sequences reconstructed through reassembly of the metagenomic sequencing reads generated in this study have been deposited in GenBank under accession numbers PZ784996 (CoV-D180) and PZ784997 (AdV-D56). Publicly available sequencing datasets and reference genomes used for comparative read-mapping and phylogenetic analyses are listed with accession numbers in the Supplementary Data. Source data underlying the figures and Supplementary Data are provided with this paper. Source data are provided with this paper.

## Code availability

The custom analysis scripts used in this study have been deposited in Zenodo under https://doi.org/10.5281/zenodo.21316345. The scripts are also provided as Supplementary Software with a README file.
