# Supplementary material for: Early-life bovine respiratory virome analysis reveals phage-driven adaptation and cross-host evolutionary signatures
Source: Nat Commun. 2026 Oct 28;17:11185. doi: 10.1038/s41467-026-77597-8 (PMC13714303; doi:10.1038/s41467-026-77597-8)
Supplement: Supplementary file 1 [file 41467_2026_77597_MOESM1_ESM.pdf]

a

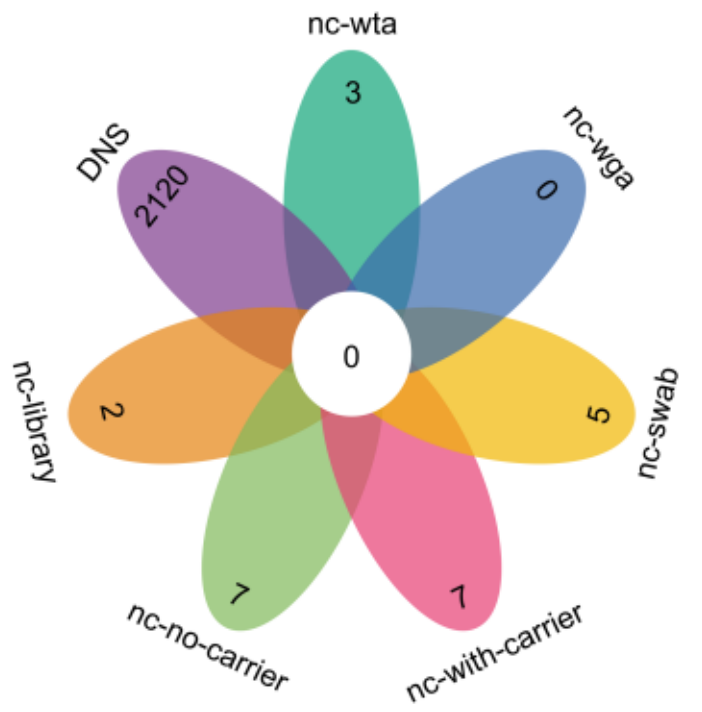

Overlap of bacteria detected in DNS and NC samples

Sample set / intersection	No. of Bacteria	Bacteria species
DNS ∩ nc-library	4	*Serratia marcescens*; *Lactobacillus crispatus*; *Lactococcus lactis*; *Gardnerella vaginalis*
DNS ∩ nc-no-carrier	1	*Variovorax paradoxus*
DNS ∩ nc-with-carrier	1	*Mesorhizobium loti*
DNS ∩ nc-swab	3	*Streptomyces albidoflavus*; *Janthinobacterium lividum*; *Arthrobacter globiformis*
DNS ∩ nc-wga	10	*Stenotrophomonas maltophilia*; *Pseudomonas fluorescens Burkholderia cepacian*; *Ralstonia pickettii; Bacillus subtilis*; *Fusobacterium sp.*; *Cupriavidus metallidurans*; *Delftia acidovorans*; *Comamonas testosterone*; *Klebsiella aerogenes*
DNS ∩ nc-wga ∩ nc-no-carrier	1	*Papillibacter sp.*
DNS ∩ nc-wga ∩ nc-with-carrier	3	*Burkholderia cenocepacia*; *Sphingomonas aerolata*; *Hymenobacter soli*
DNS ∩ nc-wga ∩ nc-swab	1	*Klebsiella pneumoniae*
DNS ∩ nc-wga ∩ nc-swab ∩ nc-with-carrier ∩ nc-library	1	*Pseudomonas aeruginosa*
DNS ∩ nc-wta	3	*Micrococcus luteus*; *Staphylococcus epidermidis*; *Corynebacterium aurimucosum*
DNS ∩ nc-wta ∩ nc-swab	1	*Cutibacterium acnes*
DNS ∩ nc-wta ∩ nc-wga	1	*Pseudomonas sp.*
DNS ∩ nc-wta ∩ nc-wga ∩ nc-library	1	*Escherichia coli*
DNS ∩ nc-wta ∩ nc-wga ∩ nc-swab	1	*Staphylococcus aureus*
DNS ∩ nc-wta ∩ nc-wga ∩ nc-swab ∩ nc-no-carrier ∩ nc-library	1	*Burkholderia sp.*
nc-wta ∩ nc-with-carrier ∩ nc-no-carrier	1	*Sphingomonas sp.*
nc-wta only	3	*Sphingomonas paucimobilis*; *Methylobacterium radiotolerans*; *Streptococcus mitis*
nc-swab only	5	*Kocuria rhizophila*; *Massilia timonae*; *Paenibacillus polymyxa*; *Brevibacterium linens*; *Acetobacter orientalis*
nc-with-carrier only	7	*Bradyrhizobium japonicum*; *Rhizobium leguminosarum*; *Paracoccus denitrificans*; *Novosphingobium aromaticivorans*; *Brevundimonas diminuta*; *Caulobacter vibrioides*; *Phenylobacterium zucineum*
nc-no-carrier only	7	*Flavobacterium johnsoniae*; *Chryseobacterium indologenes*; *Elizabethkingia anopheles*; *Pedobacter heparinus*; *Chitinophaga pinensis*; *Acidovorax temperans*; *Herbaspirillum seropedicae*
nc-library only	2	*Enterobacter cloacae*; *Citrobacter freundii*
nc-wga only	0	/
DNS only	2120	Selected representative taxa: *Pasteurella multocida; Mycoplasma bovis; Mannheimia haemolytica; Mannheimia varigena; Moraxella bovis; Histophilus somni; Mycoplasma bovirhinis; Haemophilus parainfluenzae; Haemophilus haemolyticus; Moraxella boevrei; Haemophilus influenzae; Moraxella catarrhalis; Neisseria subflava; Klebsiella quasipneumoniae; Neisseria animaloris; Actinomyces sp.; Mycoplasma bovigenitalium; Burkholderia mallei; Acinetobacter baumannii; Mycobacterium avium; Mycobacterium timonense; Moraxella nasicaprae…*

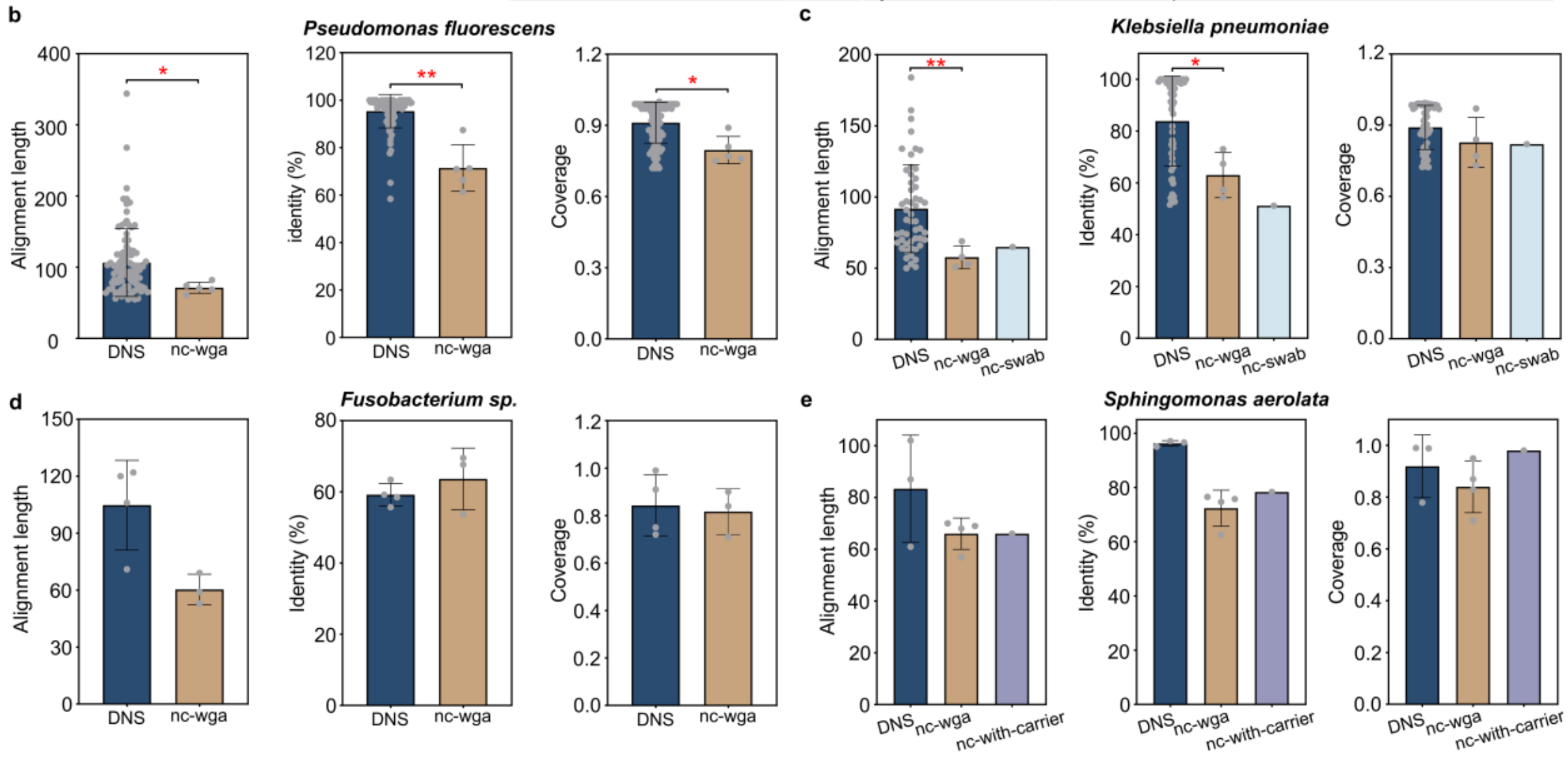

Supplementary Fig. 1 Negative controls (NCs)-based evaluation of background bacterial signatures. (a) Overlap of bacterial taxa detected in deep nasopharyngeal swab (DNS) samples and six types of NCs. (b-e) Representative DNS/NCs-overlapped bacterial taxa showing distinct interpretation patterns based on contig representation and alignment support. (b) *Pseudomonas fluorescens* and (c) *Klebsiella pneumoniae* represent DNS-dominant DNS/NCs-overlapped taxa, showing greater contig representation and alignment support in DNS samples than in NCs. (d) *Fusobacterium* sp. represents an undetermined DNS/NCs-overlapped taxon, with only minor differences in contig representation and/or alignment quality between DNS and NC samples. (e) *Sphingomonas aerolata* represents a potential NCs-associated background taxon, showing comparable or higher representation in NCs samples. Alignment length, identity, and coverage were compared between DNS and NCs samples. Bars indicate mean ± SEM, and each point represents one bacterial contig. Two-sided Wilcoxon rank-sum tests with FDR correction were used. *FDR-adjusted $P < 0.05$, **FDR-adjusted $P < 0.01$. NCs shown in panels (b-e) include whole-genome amplification control (nc-wga), environmental bench swab control (nc-swab), and nucleic acid extraction control with carrier (nc-with-carrier). Source

data are provided as a Source Data file.

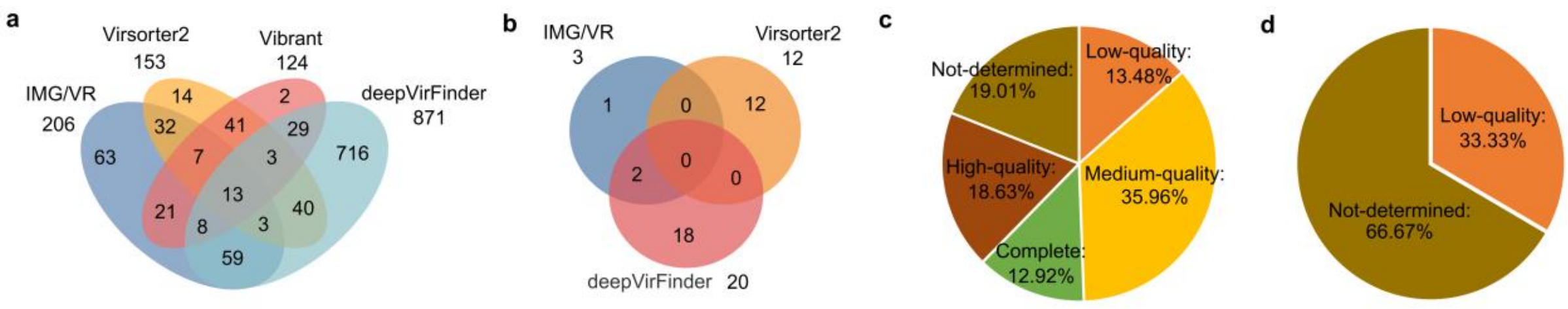

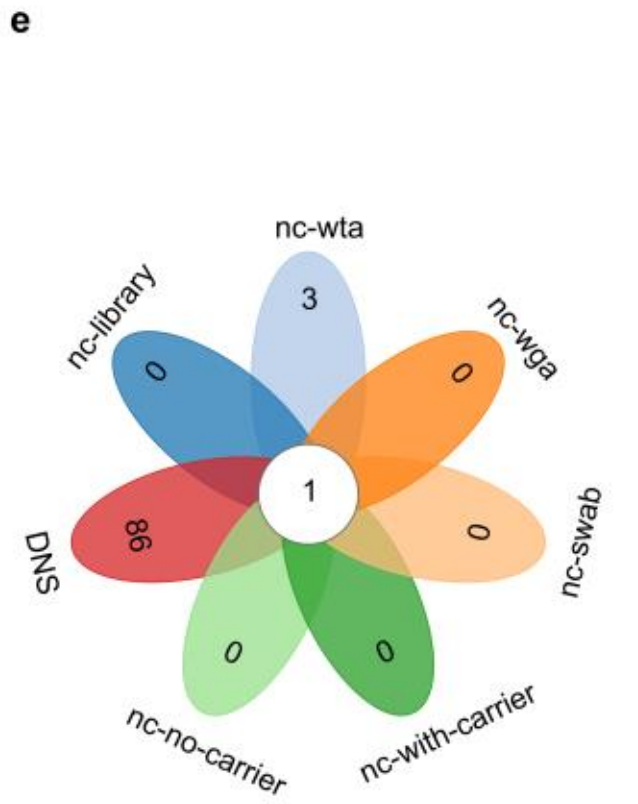

Overlap of candidate vertebrate-associated viruses detected in DNS and NC samples

Sample set / intersection	No. of virus	Virus name (Species level)
nc-wga only	0	/
nc-swab only	0	/
nc-with-carrier only	0	/
nc-no-carrier only	0	/
nc-library only	0	/
nc-library ∩ nc-wta ∩ nc-wga ∩ nc-swab ∩ nc-no-carrier	1	Sichuan mamastrovirus 8
nc-library ∩ nc-wta ∩ nc-wga ∩ nc-swab ∩ nc- with-carrier ∩ nc-no-carrier ∩ DNS	1	bovine rhinitis B virus
nc-wta ∩ DNS	1	Mamastrovirus 3
nc-wta only	3	equine infectious anemia virus; *Rhadinovirus humangamma8*; Macavirus alcelaphinegamma 1
DNS only	86	*Picobirnavirus* sp., Grus japonensis parvoviridae sp., *Faunusvirus* sp., *Mimiviridae* sp. ChoanoV1, Penaeus monodon majanivirus A, Klosneuvirus KNV1,*Edafosvirus* sp., Penicillium aurantiogriseum partitivirus 1, Catovirus CTV1, avian myeloblastosis virus, Chlorella virus XW01, feline leukemia virus, Cotonvirus japonicus, porcine picobirnavirus, scale drop disease virus, *Hyperionvirus* sp., *Terrestrivirus* sp., *Gammapartitivirus* sp., *Alphabaculovirus aucalifonicae*, Mimivirus bradfordmassiliense, Parapoynx stagnalis nucleopolyhedrovirus, Bodo saltans virus, *Partitiviridae* sp., *Aphthovirus* sp., *Betacoronavirus 1*, *Smacoviridae* sp., Cladosporium fulvum T-1 virus, Medicago sativa alphapartitivirus 1, Erysiphe necator associated betapartitivirus 2, *Betapolyomavirus pantherae*, Tupanvirus deep ocean, Paris alphapartitivirus, *Epsilonpolyomavirus bovis*, Porprismacovirus sheas2, bovine mastadenovirus B, bovine mastadenovirus C, Dragsmacovirus draga1, bovine mastadenovirus A, porcine mastadenovirus B, reindeer adenovirus 1, Phoenicopteridae parvo-like hybrid virus, tree shrew mastadenovirus A, deer mastadenovirus B, lemur mastadenovirus, simian mastadenovirus I, *Megaviridae* environmental sample, *Betapolyomavirus quartihominis*, Botryosphaeria dothidea virus 1, *Epsilonpolyomavirus caprae*, *Orthopicobirnavirus hominis*, sheep polyomavirus 1, tapir coronavirus 1044512-1, human mastadenovirus B, Erysiphe necator associated partitivirus 3, ovine mastadenovirus B, equine mastadenovirus A, simian mastadenovirus C, Medicago sativa alphapartitivirus 2, *Betapartitivirus* sp., *Betapolyomavirus myolucifugus*, Erysiphe necator associated partiti-like virus 1, rose partitivirus, Curvularia lunata partitivirus 1, Cotesia sesamiae bracovirus, cowpox virus

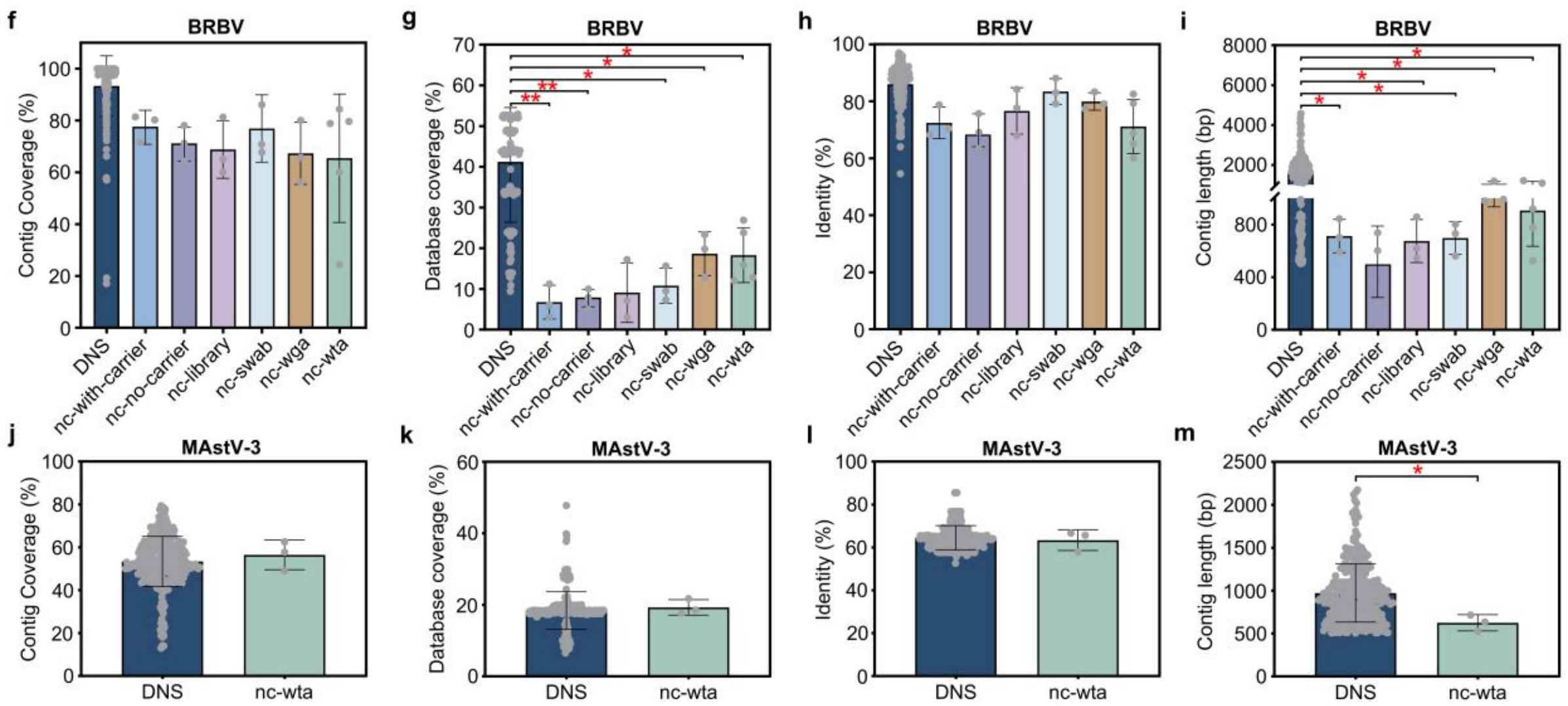

Supplementary Fig. 2 Viral contig identification, quality assessment, and NCs-based background evaluation. (a) Overlap of viral contigs identified from DNS samples using IMG/VR, VirSorter2, VIBRANT, and deepVirFinder. (b) Overlap of viral contigs identified from NCs samples using IMG/VR, VirSorter2, and deepVirFinder. (c) CheckV-based quality classification of viral contigs from DNS samples. (d) CheckV-based quality classification of viral contigs from NCs samples. (e) Overlap of candidate vertebrate-associated viruses detected in DNS samples and six types of NCs. (f-i) Comparison of bovine rhinitis B virus (BRBV)-related contigs between DNS and NCs samples based on contig coverage, database coverage, sequence identity, and contig length. (j-m) Comparison of Mamastrovirus 3 (MAstV-3)-related contigs between DNS and whole-transcriptome amplification control (nc-wta) samples based on contig coverage, database coverage, sequence identity, and contig length. Bars indicate mean ± SEM, and each point represents one viral contig. Statistical comparisons were

performed using two-sided Wilcoxon rank-sum tests with FDR correction. *FDR-adjusted $P < 0.05$, **FDR-adjusted $P < 0.01$. NCs included whole-transcriptome amplification control (nc-wta), whole-genome amplification control (nc-wga), environmental bench swab control (nc-swab), nucleic acid extraction controls with and without carrier (nc-with-carrier and nc-no-carrier), and library preparation PCR control (nc-library). Source data are provided as a Source Data file.

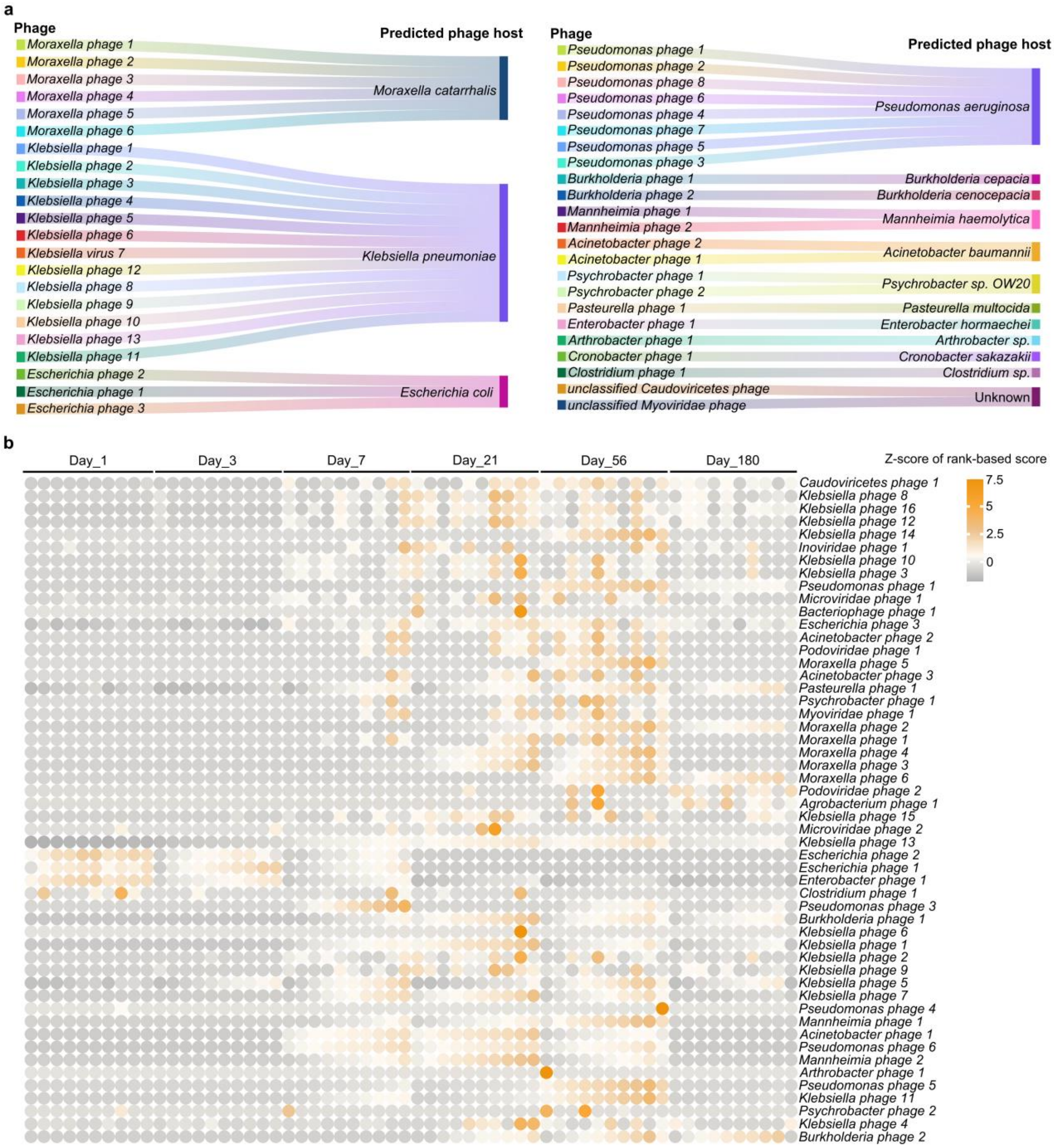

Supplementary Fig. 3 Temporal dynamics of representative calf URT phages and their predicted host associations. (a) Predicted host associations for representative phages identified in the calf URT. Sankey diagrams show links between individual phages on the left and their predicted bacterial hosts on the right. Phages without confident host assignments are labeled as "Unknown". (b) Heatmap showing temporal detection patterns of representative phages in the calf URT from birth to weaning at Days 1, 3, 7, 21, 56, and 180. Raw reads were first summarized at the phage species level within each sample and then transformed into rank-based scores, with zero values retained as zero. Mean rank-based scores were calculated for each developmental stage and standardized by row for visualization. Colors indicate the row Z-score of species-level rank-based scores. Source data are provided as a Source Data file.

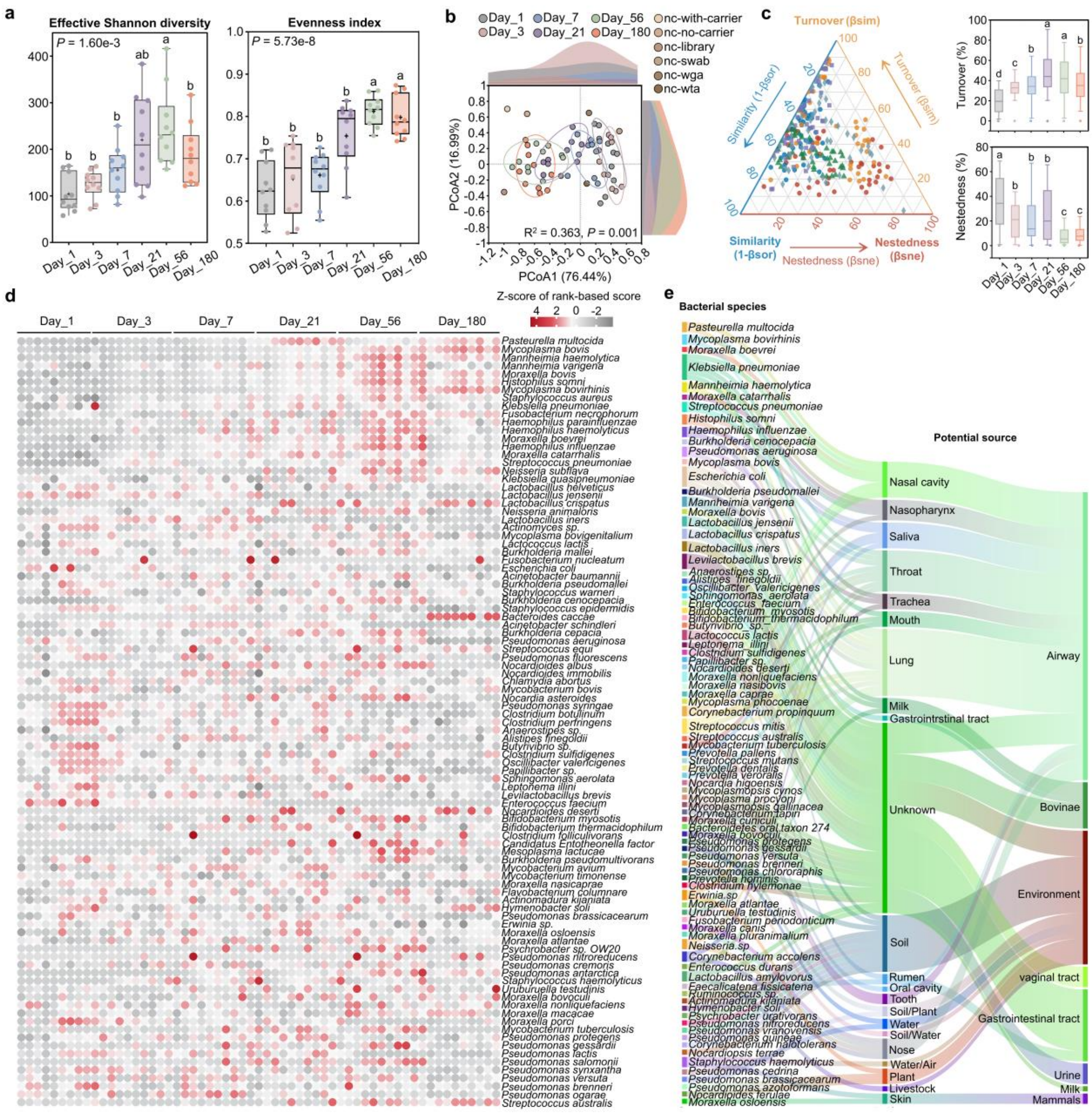

Supplementary Fig. 4 Early-life succession and potential sources of bacterial communities in the URT of beef calves. (a) Alpha diversity of URT bacterial communities across six sampling time points, including effective Shannon diversity and evenness index. Each point represents one biologically independent DNS sample; n = 10 calves per time point. Box plots show the median, 25th-75th percentiles, and whiskers extending to 1.5× interquartile range (IQR). (b) PCoA based on Spearman rank-correlation distances of within-sample rank-order-transformed raw read-count profiles, showing bacterial community dissimilarities across sampling time points and NCs. Each point represents one DNS or NC sample; n = 60 DNS samples and n = 6 NCs samples. The effect of sampling time on DNS bacterial profiles was tested using PERMANOVA with 999 permutations constrained by calf identity. (c) Partitioning of bacterial beta diversity into similarity, turnover, and nestedness components using within-time-point pairwise comparisons. The ternary plot includes n = 270 pairwise comparisons in total; box plots summarize n = 45 comparisons per time point and are defined as in a. (d) Heatmap showing temporal patterns of bacterial species across early life. Values represent Z-

score-transformed rank-based scores within each bacterial species. (e) Sankey diagram showing the potential source of the bacterial species in the calves URT. Time-point differences in (a) and (c) were tested using Friedman tests followed by Dunn's multiple-comparison tests with FDR correction. Different letters indicate significant differences between time points; exact adjusted *P* values are provided in the Source Data file. Source data are provided as a Source Data file.

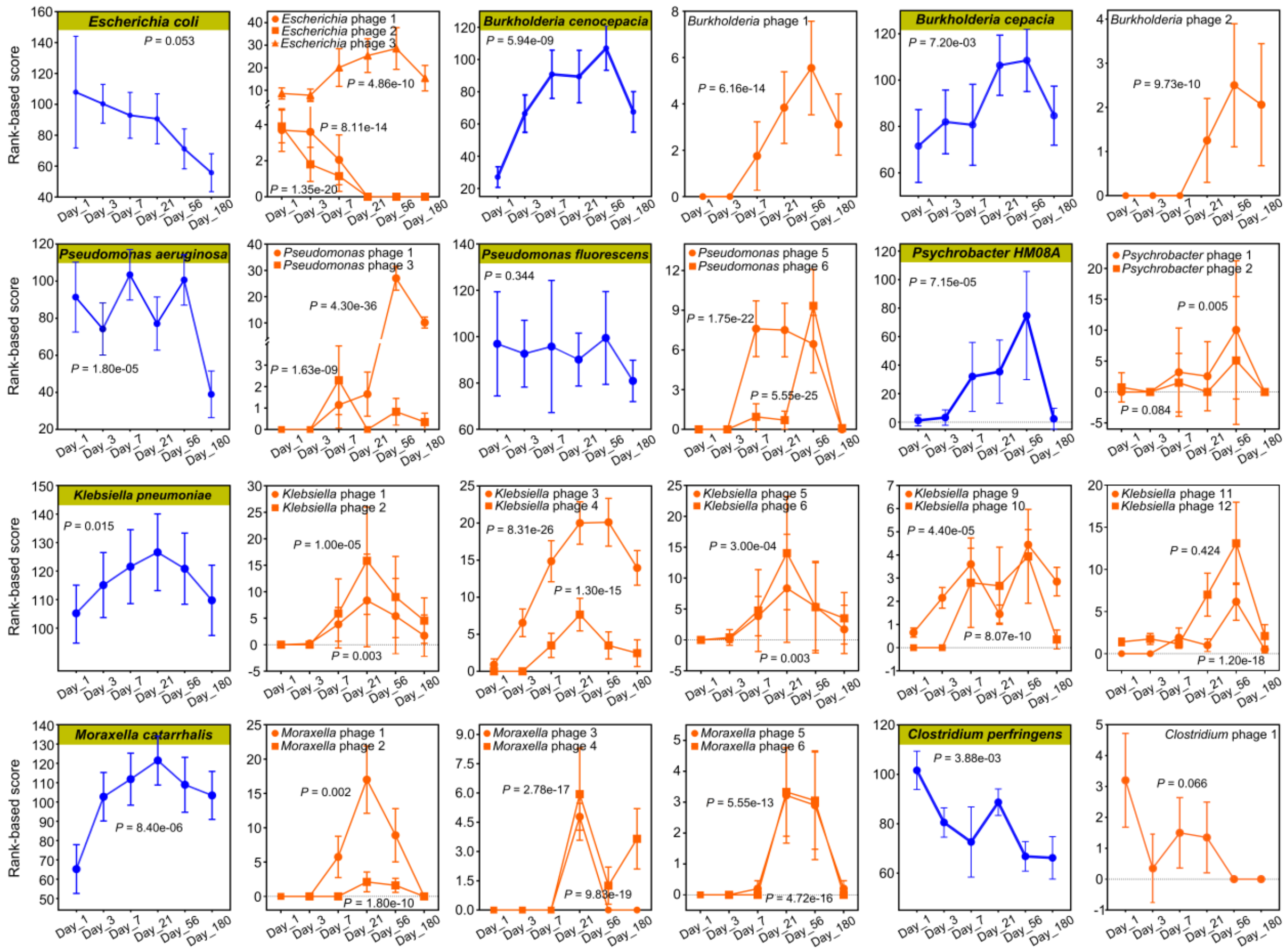

Supplementary Fig. 5 Temporal dynamics of dominant phages and their predicted bacterial hosts in the calf URT. Line plots show temporal changes in rank-based scores of representative phages and their predicted bacterial hosts from birth to weaning. Bacterial hosts are shown in blue, and associated phages are shown in orange. Rank-based scores were calculated from read-count profiles within each sample, with zero values retained as zero. Data are presented as mean ± SEM; n = 10 biologically independent DNS samples per time point. Time-point differences were tested using Friedman tests followed by Dunn's post hoc multiple comparisons, with Benjamini-Hochberg FDR correction. Exact adjusted *P* values are provided in the Supplementary Table and Source Data file. Source data are provided as a Source Data file.

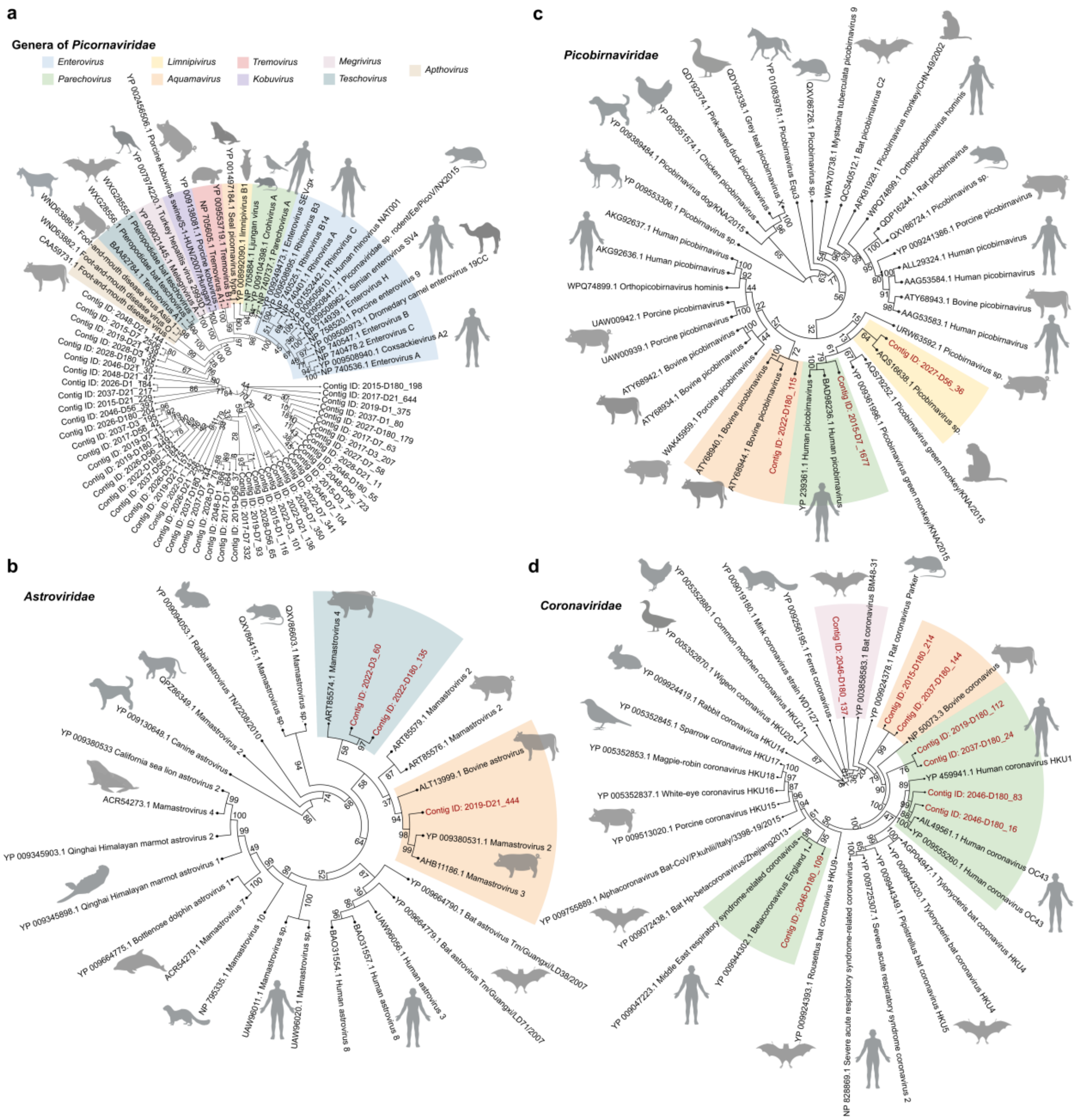

Supplementary Fig. 6 Phylogenetic relationships of representative bovine-derived RNA virus families identified in the calf URT. Maximum-likelihood phylogenetic trees were constructed for major RNA virus families detected in this study, including (a) *Picornaviridae*, (b) *Astroviridae*, (c) *Picobirnaviridae*, and (d) *Coronaviridae*. Bovine-derived viral contigs recovered in this study are highlighted in color and labeled in red, while reference sequences from publicly available databases are shown in black. Host silhouettes indicate the reported host origin of reference viruses. Bootstrap support values (>70%) are shown at key nodes (Animal host icons were created in BioRender.com, https://BioRender.com/whsvu6d).

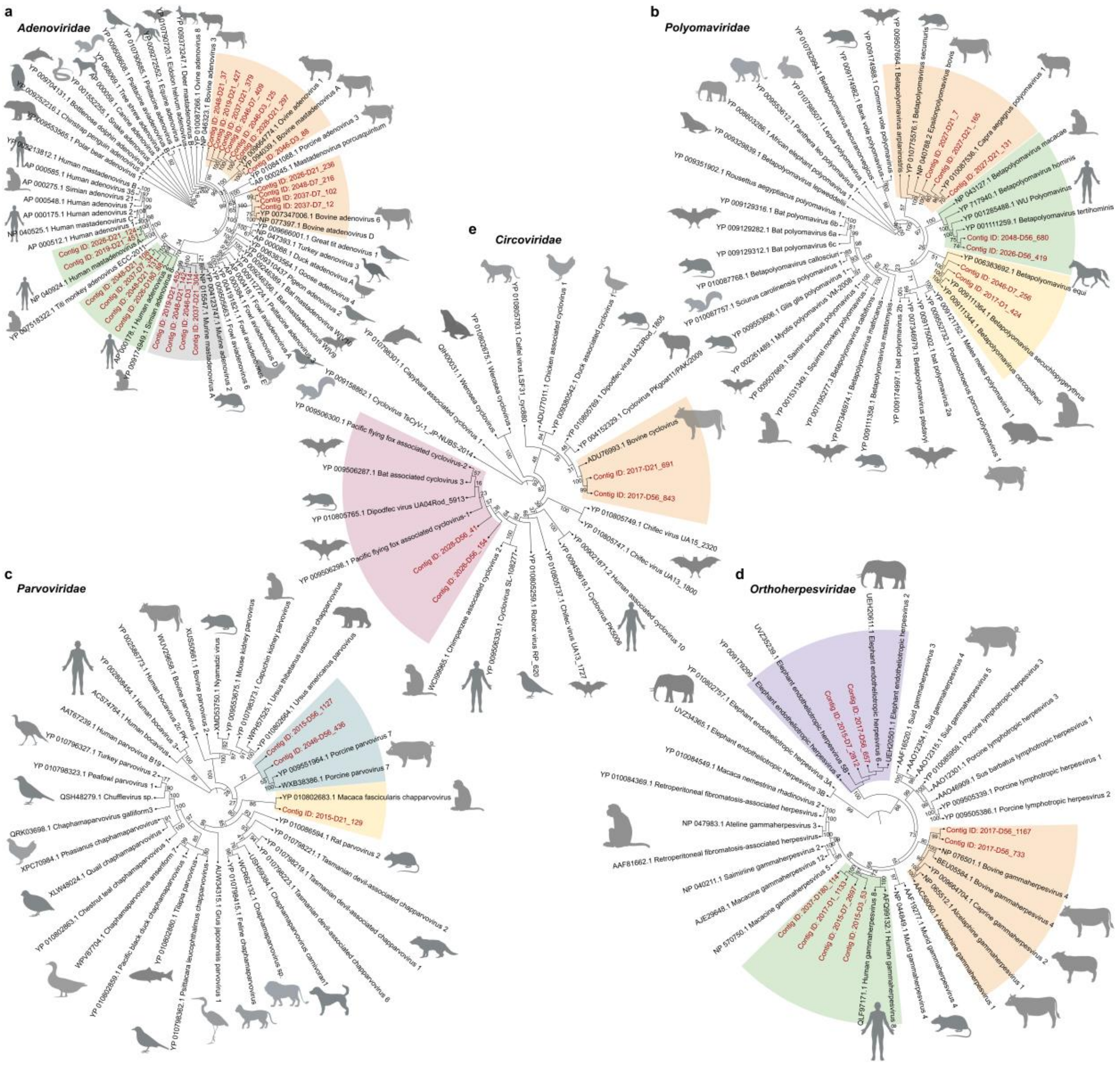

Supplementary Fig. 7 Phylogenetic relationships of representative bovine-derived DNA virus families identified in the calf URT. Maximum-likelihood phylogenetic trees were constructed for major DNA virus families detected in this study, including (a) *Adenoviridae*, (b) *Polyomaviridae*, (c) *Parvoviridae*, (d) *Orthoherpesviridae*, and (e) *Circoviridae*. Bovine-derived viral contigs recovered in this study are highlighted in color and labeled in red, while reference sequences retrieved from public databases are shown in black. Host silhouettes indicate the reported host origin of reference viruses. Bootstrap support values (>70%) are shown at key nodes (Animal host icons were created in BioRender.com, https://BioRender.com/whsvu6d).
