# Supplementary material for: Early-life bovine respiratory virome analysis reveals phage-driven adaptation and cross-host evolutionary signatures
Source: Nat Commun. 2026 Oct 28;17:11185. doi: 10.1038/s41467-026-77597-8 (PMC13714303; doi:10.1038/s41467-026-77597-8)
Supplement: Supplementary file 2 [file 41467_2026_77597_MOESM5_ESM.pdf]

nature portfolio

Corresponding author(s): Professor Le Luo Guan

Last updated by author(s): Jul 15, 2026

Reporting Summary

Nature Portfolio wishes to improve the reproducibility of the work that we publish. This form provides structure for consistency and transparency in reporting. For further information on Nature Portfolio policies, see our Editorial Policies and the Editorial Policy Checklist.

Statistics

For all statistical analyses, confirm that the following items are present in the figure legend, table legend, main text, or Methods section.

n/a	Confirmed	
☐	☒	The exact sample size (*n*) for each experimental group/condition, given as a discrete number and unit of measurement
☐	☒	A statement on whether measurements were taken from distinct samples or whether the same sample was measured repeatedly
☐	☒	The statistical test(s) used AND whether they are one- or two-sided<br>*Only common tests should be described solely by name; describe more complex techniques in the Methods section.*
☒	☐	A description of all covariates tested
☐	☒	A description of any assumptions or corrections, such as tests of normality and adjustment for multiple comparisons
☐	☒	A full description of the statistical parameters including central tendency (e.g. means) or other basic estimates (e.g. regression coefficient) AND variation (e.g. standard deviation) or associated estimates of uncertainty (e.g. confidence intervals)
☐	☒	For null hypothesis testing, the test statistic (e.g. *F*, *t*, *r*) with confidence intervals, effect sizes, degrees of freedom and *P* value noted<br>*Give P values as exact values whenever suitable.*
☒	☐	For Bayesian analysis, information on the choice of priors and Markov chain Monte Carlo settings
☐	☒	For hierarchical and complex designs, identification of the appropriate level for tests and full reporting of outcomes
☐	☒	Estimates of effect sizes (e.g. Cohen's *d*, Pearson's *r*), indicating how they were calculated

Our web collection on statistics for biologists contains articles on many of the points above.

Software and code

Policy information about availability of computer code

Data collection	No software was used for sample collection. Sample collection and metadata recording were performed manually at each longitudinal sampling time point.
Data analysis	Data analysis was performed using Trimmomatic v0.39, FastQC v0.12.1, MultiQC v1.14, BWA v0.7.18, SAMtools v1.22.1, MEGAHIT v1.2.9, VirSorter2 v2.0, DeepVirFinder v1.1, VIBRANT v1.2.1, CheckV v0.7.0, PhageAnnotator, VITAP, PhaBOX v2.1.7, DIAMOND v2.9.0+/v2.14.1, MEGAN6, SPAdes v3.15.5, Prodigal v2.6.3, InterProScan, Geneious Prime v2025.2.2, MAFFT v7.525, TrimAl v1.5, IQ-TREE v2.3.5, iTOL, SRA Toolkit v3.0.0, Bowtie2 v2.5, Cytoscape v3.9.1, R v4.3.1, GraphPad Prism v9.5, and the R packages described in the Methods. Custom scripts were used for rank-based transformation, diversity analysis, temporal trend analysis, competitive mapping summaries, sliding-window analysis, and figure generation. These scripts are available in Zenodo under DOI: https://doi.org/10.5281/zenodo.21316345 and are also provided as Supplementary Software with a README file and example input files.

For manuscripts utilizing custom algorithms or software that are central to the research but not yet described in published literature, software must be made available to editors and reviewers. We strongly encourage code deposition in a community repository (e.g. GitHub). See the Nature Portfolio guidelines for submitting code & software for further information.

Data

Policy information about availability of data

All manuscripts must include a data availability statement. This statement should provide the following information, where applicable:

- Accession codes, unique identifiers, or web links for publicly available datasets
- A description of any restrictions on data availability
- For clinical datasets or third party data, please ensure that the statement adheres to our policy

Raw sequencing data generated in this study have been deposited in the NCBI Sequence Read Archive (SRA) under BioProject accession numbers PRJNA1259590 and PRJNA1259072. The data are publicly available.

Research involving human participants, their data, or biological material

Policy information about studies with human participants or human data. See also policy information about sex, gender (identity/presentation), and sexual orientation and race, ethnicity and racism.

Reporting on sex and gender	The publicly available dataset included 115 male and 106 female children. Sex was reported in the original study but was not analyzed as a variable in the present sequence-mapping analysis.
Reporting on race, ethnicity, or other socially relevant groupings	Race, ethnicity, and other socially relevant groupings were not reported in the original publication or publicly accessible dataset metadata and were therefore not assessed in the present study.
Population characteristics	The publicly available dataset comprised nasopharyngeal metatranscriptomic samples from 221 children aged 2–11 years with clinically diagnosed acute sinusitis. Participant characteristics were reported in the original study.
Recruitment	No participants were recruited directly for the present study. The publicly available data were generated from children recruited between February 2016 and April 2022 from six outpatient centers as part of a multicenter randomized clinical trial.
Ethics oversight	The present study involved secondary analysis of publicly available human respiratory sequencing data. Ethics approval, informed consent, participant recruitment, and sample collection were governed by the original study and clinical trial (NCT02554383). No identifiable participant information was accessed in the present analysis.

Note that full information on the approval of the study protocol must also be provided in the manuscript.

Field-specific reporting

Please select the one below that is the best fit for your research. If you are not sure, read the appropriate sections before making your selection.

☒ Life sciences ☐ Behavioural & social sciences ☐ Ecological, evolutionary & environmental sciences

For a reference copy of the document with all sections, see nature.com/documents/nr-reporting-summary-flat.pdf

Life sciences study design

All studies must disclose on these points even when the disclosure is negative.

Sample size	Ten newborn beef calves were included in this longitudinal study, and deep nasopharyngeal swab samples were collected at six developmental time points: Day 1, Day 3, Day 7, Day 21, Day 56, and Day 180 after birth, with Day 180 corresponding to weaning. This generated a total of 60 biological samples. The sample size was determined by the availability of a longitudinal calf cohort with repeated sampling throughout early life. No formal statistical power calculation was performed because this was an exploratory longitudinal metagenomic study.
Data exclusions	No enrolled animals or biological samples were excluded from the study. Sequencing reads, contigs or taxonomic assignments that did not meet predefined quality-control and filtering criteria were removed as described in the Methods. Negative-control-derived viral contigs that were classified as low-quality or not-determined by CheckV were excluded from downstream phage analyses.
Replication	Biological replication was achieved through longitudinal sampling of 10 calves across six developmental time points. Multiple negative controls were processed alongside biological samples to evaluate potential background contamination. Bioinformatic analyses were performed using predefined pipelines and documented software versions.
Randomization	Randomization was not applicable because this was an observational longitudinal study without experimental treatment allocation. Calves were sampled repeatedly at predefined developmental time points.
Blinding	Blinding was not applicable because samples were collected at predefined developmental time points and analyzed using predefined bioinformatic pipelines.

Reporting for specific materials, systems and methods

We require information from authors about some types of materials, experimental systems and methods used in many studies. Here, indicate whether each material, system or method listed is relevant to your study. If you are not sure if a list item applies to your research, read the appropriate section before selecting a response.

Materials & experimental systems

n/a	Involved in the study
☒	☐ Antibodies
☒	☐ Eukaryotic cell lines
☒	☐ Palaeontology and archaeology
☐	☒ Animals and other organisms
☒	☐ Clinical data
☒	☐ Dual use research of concern
☒	☐ Plants

Methods

n/a	Involved in the study
☒	☐ ChIP-seq
☒	☐ Flow cytometry
☒	☐ MRI-based neuroimaging

Animals and other research organisms

Policy information about studies involving animals; ARRIVE guidelines recommended for reporting animal research, and Sex and Gender in Research

Laboratory animals	Not applicable. This study did not use laboratory animals. Samples were collected from beef calves in a longitudinal animal cohort.
Wild animals	Not applicable. This study did not involve wild animals.
Reporting on sex	The study included 10 healthy beef calves, comprising 6 males and 4 females, managed under the same herd conditions. Both sexes were included in the study design. Sex-associated differences in virome and bacteriome community composition were assessed using PCoA, PERMANOVA, and PERMDISP. No significant sex-associated differences in community composition or multivariate dispersion were detected in either dataset.
Field-collected samples	Deep nasopharyngeal swab samples were collected longitudinally from ten newborn beef calves (Bos taurus; Angus × Hereford cross; n = 10) maintained at the Animal Diseases Research Institute (ADRI), Lethbridge, Alberta, Canada. Samples were collected at six developmental time points from birth to weaning. Animals were housed and managed under standard research herd conditions, and no experimental housing manipulation was performed. Samples were collected using sterile procedures, transported under cold-chain conditions, and stored at −80 °C until nucleic acid extraction. Negative controls were processed alongside biological samples during nucleic acid extraction, amplification, and library preparation to assess potential background contamination.
Ethics oversight	Ethical approval was granted by the Animal Care Committee of the Canadian Food Inspection Agency, and all procedures were performed in accordance with the guidelines of the Canadian Council on Animal Care (CCAC, 2009).

Note that full information on the approval of the study protocol must also be provided in the manuscript.

Plants

Seed stocks	Not applicable
Novel plant genotypes	Not applicable
Authentication	Not applicable
