# Supplementary material for: Early-life bovine respiratory virome analysis reveals phage-driven adaptation and cross-host evolutionary signatures
Source: Nat Commun. 2026 Oct 28;17:11185. doi: 10.1038/s41467-026-77597-8 (PMC13714303; doi:10.1038/s41467-026-77597-8)
Supplement: Supplementary file 3 [file 41467_2026_77597_MOESM6_ESM.pdf]

nature portfolio

Peer Review File

Early-life bovine respiratory virome analysis reveals phage-driven adaptation and cross-host evolutionary signatures

Corresponding Author: Professor LeLuo Guan

This file contains all reviewer reports in order by version, followed by all author rebuttals in order by version.

Version 0:

Reviewer comments:

Reviewer #1

(Remarks to the Author)
The manuscript titled "Early-life bovine respiratory virome ecology reveals phage-driven adaptation and cross-host evolutionary signatures with One Health relevance" by Wang et. al. aims to understand the ecology of the upper respiratory tract virome of beef calves from birth to weaning through viral enrichment and metagenomic sequencing.

Overall, the manuscript is well written and relevant to the field. Authors do a great job of describing both the assembly of the communities and the ecological dynamics, and of linking one-health relevance through extensive comparisons with human databases.

The only major issue identified was the lack of mention of negative controls. This must be either further clarified or addressed in the discussion.

Please see specific feedback below:

Format:
Appropriate

Content:
- Introduction
Well written, no issues identified.

- Methods

o Sampling
 Ethics approval
Appropriate

- Laboratory Methods

 Controls
Negative controls are lacking.
Were there any negative extraction, amplification, and sequencing controls performed?
This is quite critical for a study on low biomass samples that relies heavily on whole genome amplification. Reagent and kit contamination and background "microbiome" is a well known phenomena that must be addressed. If authors did not perform such controls along with the samples, this must be addressed in the limitations.

- Bioinformatics
I appreciate that the authors made their code available both for bioinformatics and statistical analysis. I want to congratulate the team for providing such well-organized and comprehensive code.

- Data availability
Confirmed that PRJNA1259072 is available. Could not find PRJNA125959.

- Statistical analysis
All statistical analyses are well described and appropriate.

- Results
Results are well described and not overinterpreted.

- Discussion
While this reviewer understands the challenges associated with working with low-biomass samples, the lack of negative controls is troubling. The findings align with what we know about the upper respiratory tract microbiome of bovines, but many taxa have also been reported as reagent contaminants. This limitation needs to be addressed in the discussion.

- Conclusions
Well stated and in line with the data presented.

Figures:

Figure 1: typos – swabs not swaps, membrane not membrance

(Remarks on code availability)
I appreciate that the authors made their code available both for bioinformatics and statistical analysis. I want to congratulate the team for providing such well-organized and comprehensive code.

Reviewer #2

(Remarks to the Author)
In this manuscript, the authors describe the characterization of the upper respiratory tract virome and microbiome of calves over the course of their first 180 days of life. The authors used a viral enrichment strategy coupled with reverse transcription and metagenomic sequencing to capture DNA and RNA viruses, as well as the bacterial community. The authors noted clear transitions in viral species composition and abundance over time, which reflected shifts in animal age, feeding, and living/space conditions.

Although this work is quite interesting and represents a novel contribution to the field in terms of the comprehensive time series characterization, I have concerns about the use of abundance/relative abundance data and their subsequent statistical analysis. Random amplification techniques can be prone to bias. The kit used here has been demonstrated to have GC bias with low biomass samples, and although it generally recapitulates rank-order abundances in mixed communities, relative abundances are not necessarily preserved in a consistent manner with communities of known composition (https://pmc.ncbi.nlm.nih.gov/articles/PMC5363268/#s13). Given these potential limitations, I would encourage the authors to consider a shift to rank-order metrics and parametric statistics or ask for additional demonstration that the random amplification step has not introduced bias into these results.

Additionally, I would encourage the authors to provide full citations for all of the bioinformatics tools and reference databases presented in the methods section. Although some are provided, many are missing.

(Remarks on code availability)
The scripts used are detailed and well annotated. They could be reproduced.

Version 1:

Reviewer comments:

Reviewer #1

(Remarks to the Author)
The authors have appropriately addressed this reviewer’s comments. This was a well written and relevant manuscript.

A few typos were noticed; please carefully review the manuscript, particularly the figures and axis labels.

Supplementary Figures 1 and 2 and all other figures:

nc-with-carries
nc-with-carrie
should be nc-with-carrier

(Remarks on code availability)

Reviewer #3

(Remarks to the Author)
Thanks for the edits and the response to the reviewers comments. I have a few minor comments.

Please note that a virus name and species name are two different things. Just as for example Joe Brown is a name of a person but is a member of the species Homo sapiens. Thus Bovine rhinitis B virus, Bovine gammaherpesvirus 4 and Human gammaherpesvirus 8 are a virus names and not a species and should not be italicized, and the first letter in human, bovine should not be capital letter.

Furthermore, in the case of virus taxonomy, all taxa names (expect virus name) are italicized. Please italicize Picornavirales, Caudoviricetes, Cirlivirales, Embecovirus.

(Remarks on code availability)
na

Open Access This Peer Review File is licensed under a Creative Commons Attribution 4.0 International License, which permits use, sharing, adaptation, distribution and reproduction in any medium or format, as long as you give appropriate credit to the original author(s) and the source, provide a link to the Creative Commons license, and indicate if changes were made.
In cases where reviewers are anonymous, credit should be given to 'Anonymous Referee' and the source.
The images or other third party material in this Peer Review File are included in the article's Creative Commons license, unless indicated otherwise in a credit line to the material. If material is not included in the article's Creative Commons license and your intended use is not permitted by statutory regulation or exceeds the permitted use, you will need to obtain permission directly from the copyright holder.
To view a copy of this license, visit https://creativecommons.org/licenses/by/4.0/

Responses to Reviewers' Comments

Reviewer #1 (Remarks to the Author):
The manuscript titled "Early-life bovine respiratory virome ecology reveals phage-driven adaptation and cross-host evolutionary signatures with One Health relevance" by Wang et. al. aims to understand the ecology of the upper respiratory tract virome of beef calves from birth to weaning through viral enrichment and metagenomic sequencing. Overall, the manuscript is well written and relevant to the field. Authors do a great job of describing both the assembly of the communities and the ecological dynamics, and of linking one-health relevance through extensive comparisons with human databases. The only major issue identified was the lack of mention of negative controls. This must be either further clarified or addressed in the discussion.
Please see specific feedback below:

Format:
Appropriate

Content:
- Introduction
Well written, no issues identified.
- Methods
o Sampling
♣ Ethics approval
Appropriate

- Laboratory Methods

♣ Controls
Negative controls are lacking.
Were there any negative extraction, amplification, and sequencing controls performed?
This is quite critical for a study on low biomass samples that relies heavily on whole genome amplification. Reagent and kit contamination and background "microbiome" is a well-known phenomenon that must be addressed. If authors did not perform such controls along with the samples, this must be addressed in the limitations.
Replies: We agree with the reviewer that negative controls (NCs) are essential for low-biomass respiratory tract samples. Actually, we included NCs throughout the experimental workflow but missed the presentation of the information in the original submission. In the revised manuscript, we have included information of six types of NCs, including whole-transcriptome amplification control (nc-wta), whole-genome amplification control (nc-wga), environmental bench swab control (nc-swab), nucleic acid extraction controls with and without carrier (nc-with-carrier and nc-no-carrier), and library preparation PCR control (nc-library). The information has now included in the Methods (lines 554-561, 590-593, 616-620, and 622-624), and the results from the NCs are included in the revised sequencing summary and contig assembly statistics (**Supplementary Tables 1-6**), as well as the bacterial background assessment (**Supplementary Tables 7-8**), phage analysis and quality evaluation

(**Supplementary Tables 9-13**), and vertebrate-associated virus background assessment (**Supplementary Table 15**).

1. Assessment of NC-derived background bacterial signatures

(1) Identification of bacteria in NC samples

To evaluate potential NC-derived background bacterial signatures, NCs- and DNS-derived contigs were processed using the same bacterial annotation and filtering strategy. Bacterial contigs >1 kb were annotated using DIAMOND BLASTx against the NCBI NR protein database, and only matches with an alignment length > 50, identity > 50%, query coverage > 70%, and E-value < 1E-10 were retained for downstream bacterial classification. Using these criteria, 10,709 bacterial contigs were retained from DNS samples, whereas only 124 bacterial contigs were retained from NCs samples. Most bacterial taxa were DNS-specific (n = 2,120), while a subset of bacterial taxa overlapped between DNS and NCs samples (**Supplementary Table 7**). To avoid overinterpreting NC detection as direct contamination, we did not automatically remove all DNS/NC-overlapped taxa. Instead, overlapping bacterial taxa were evaluated based on NC type, contig number, alignment length, identity (%), and query coverage (%). Based on these metrics, the DNS/NCs-overlapped taxa were classified into three categories (**Supplementary Table 8; Supplementary Fig. 1**): (i) DNS-dominant taxa, which showed significantly higher contig representation and generally stronger alignment support in DNS samples than in NCs samples, including *Pseudomonas fluorescens*, *Burkholderia cenocepacia*, *Klebsiella pneumoniae*, *Pseudomonas aeruginosa*, *Escherichia coli*, *Staphylococcus aureus*, and *Burkholderia* sp. These taxa were retained in downstream analyses and interpreted as DNS-dominant bacterial signature; (ii) NC-dominant background taxa, which showed higher representation in NC samples than in DNS samples, including *Papillibacter* sp., *Sphingomonas aerolata*, and *Hymenobacter soli*. These taxa were excluded from subsequent analyses; and (iii) Undetermined DNS/NC-overlapped taxa, which were detected in both DNS and NC samples but showed only minor differences in contig representation and alignment metrics between DNS and NCs samples, including *Lactococcus lactis*, *Variovorax paradoxus*, *Fusobacterium* sp., *Cupriavidus metallidurans*, *Mesorhizobium loti*, and *Klebsiella aerogenes*. These taxa were not used to support the main biological conclusions, and their DNS-associated biological relevance requires further validation

(2) PCoA analysis of DNS and NCs bacterial profiles

To further assess if NCs-derived background bacterial signatures influenced the overall bacterial community structure, we performed PCoA including both DNS and NCs samples based on rank-order dissimilarities. NCs samples did not form the main DNS clusters, although some NC samples were located close to the DNS sample groups (**Supplementary Fig. 4b**).

2. Evaluation of NCs samples in the phage analysis branch

(1) Identification and cross-tool consistency of candidate viral contigs in NCs.

NCs samples were processed using the same phage identification pipeline as DNS samples, and candidate viral contigs are now provided in **Supplementary Tables 9-13**. After integration and duplicate removal, a total of 33 non-redundant contigs were identified in NCs samples, compared to 1,079 in DNS samples. To further assess the reliability of these signatures, a Venn diagram summarizing cross-tool identification in NCs samples has been added as **Supplementary Fig. 2b**. Compare to DNS samples, NCs-derived contigs showed minimal overlap among tools, indicating a lack of consistent viral signatures.

(2) Quality evaluation of NCs-derived contigs.

CheckV-based quality assessment showed that all 33 NC-derived contigs were classified as either “low-quality” (11/33, 33.33%) or “not-determined” (22/33, 66.67%), with no medium-

or high-quality viral genomes recovered. These NCs-derived contigs were not included from downstream analyses (**Supplementary Table 13; Supplementary Fig. 2d**).

3. Assessment of NCs samples in the vertebrate-associated virus analysis branch

(1) Identification of candidate vertebrate-associated viruses in NCs samples

To evaluate potential NCs-derived background viral signatures in the vertebrate-associated virus branch, NCs-derived contigs were annotated against the same NR-viral and NT-viral databases using the same filtering criteria as DNS-derived contigs. Viruses shared between DNS and NCs samples were further assessed using contig-level metrics, including contig coverage (%), database coverage (%), sequence identity (%), and contig length. Shared viruses with comparable or stronger support in NCs were annotated as NCs-associated viral signatures, whereas viruses with significantly stronger contig-level support in DNS samples were retained as DNS-dominant viruses. Using the same thresholds applied to DNS samples (E-value ≤ 1E−10 and identity ≥ 50%), 19 and 25 candidate vertebrate virus-related contigs were identified from the NR and NT annotations, respectively. After removing redundant contigs detected across the two databases, 35 non-redundant vertebrate virus-associated contigs were retained for further evaluation (**Supplementary Table 15**).

(2) Comparison between NCs and DNS samples to assess potential background viral signatures

To determine whether NCs-derived candidate vertebrate-associated viruses overlapped with DNS-derived viruses, viral identifications were performed at species level, and duplicated virus derived from multiple contigs were removed. This analysis identified 86 candidate vertebrate-associated viruses detected only in DNS samples, while only two viruses were shared between DNS and NCs samples: *Bovine rhinitis B virus* (BRBV), which was detected in DNS samples and multiple NC types, and *Mamastrovirus 3* (MAstV-3), which was shared only between DNS and nc-wta samples (**Supplementary Fig. S2e; Supplementary Table 15**). Because BRBV and MAstV-3 were detected in both DNS and NC samples, we further compared contig-level metrics, including contig coverage (%), database coverage (%), sequence identity (%), and contig length. For BRBV, DNS-derived contigs showed significantly higher database coverage (%) and longer contig lengths than NC-derived contigs (**Supplementary Fig. 2f-i**). Therefore, BRBV was classified as a DNS-associated virus and retained for downstream biological interpretation. For MAstV-3, although DNS-derived contigs were longer than those detected in nc-wta, contig coverage (%), database coverage (%), and sequence identity (%) did not differ significantly between DNS and nc-wta samples (**Supplementary Fig. 2j-m**). Therefore, MAstV-3 was considered an NCs-associated background viral signatures and was excluded from downstream analyses and main biological conclusions.

(3) PCoA analysis of DNS and NC viral profiles

To further evaluate if NC-derived viral signatures affected the overall viral community structure, we performed PCoA including both DNS and NCs samples based on rank-order dissimilarities. NCs samples were generally separated from the main DNS sample groups and did not reproduce the age-associated patterns observed in DNS samples. This result supports that NCs-derived viral signatures were compositionally distinct from the dominant DNS-associated viral patterns observed in this study (**Fig. 2b**).

Overall, the NCs analyses provided an additional quality-control assessment for both viral and bacterial datasets. In the phage branch, all NCs-derived candidate viral contigs were low-quality or not-determined and were excluded from downstream analyses. In the vertebrate-associated virus branch, MAstV-3 were considered as NCs-associated background viral signature. In the bacterial analysis, NCs-derived bacterial contigs showed a small fraction of DNS-derived bacterial contigs, and DNS/NCs-overlapped taxa were interpreted according to contig representation and alignment quality. Taxa with comparable or higher representation in NCs samples were considered potential NCs-associated background microbial signatures and were not used to support the main conclusions. Therefore, the NCs analyses clarified the evaluation of potential background microbial signatures but did not alter the major findings of virome, bacteriome, or phage-host in this study. The Methods, Results, Discussion, Supplementary Tables, and Supplementary Figures have been revised accordingly.

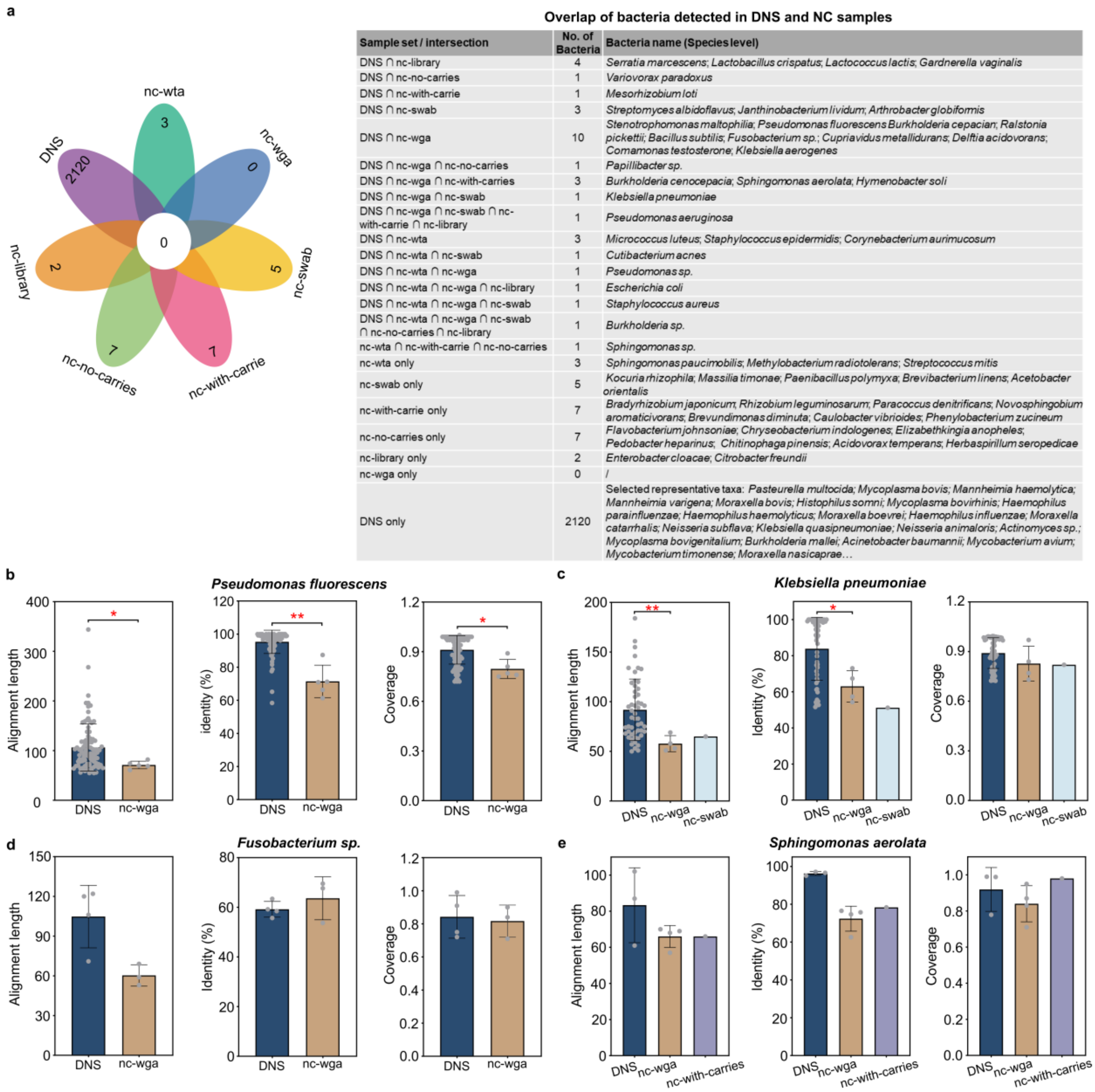

Overlap of bacteria detected in DNS and NC samples

Sample set / intersection	No. of Bacteria	Bacteria name (Species level)
DNS ∩ nc-library	4	*Serratia marcescens*; *Lactobacillus crispatus*; *Lactococcus lactis*; *Gardnerella vaginalis*
DNS ∩ nc-no-carries	1	*Variovorax paradoxus*
DNS ∩ nc-with-carrie	1	*Mesorhizobium loti*
DNS ∩ nc-swab	3	*Streptomyces albidoflavus*; *Janthinobacterium lividum*; *Arthrobacter globiformis*
DNS ∩ nc-wga	10	*Stenotrophomonas maltophilia*; *Pseudomonas fluorescens Burkholderia cepacian*; *Ralstonia pickettii*; *Bacillus subtilis*; *Fusobacterium sp.*; *Cupriavidus metallidurans*; *Delftia acidovorans*; *Comamonas testosterone*; *Klebsiella aerogenes*
DNS ∩ nc-wga ∩ nc-no-carries	1	*Papillibacter sp.*
DNS ∩ nc-wga ∩ nc-with-carries	3	*Burkholderia cenocepacia*; *Sphingomonas aerolata*; *Hymenobacter soli*
DNS ∩ nc-wga ∩ nc-swab	1	*Klebsiella pneumoniae*
DNS ∩ nc-wga ∩ nc-swab ∩ nc-with-carrie ∩ nc-library	1	*Pseudomonas aeruginosa*
DNS ∩ nc-wta	3	*Micrococcus luteus*; *Staphylococcus epidermidis*; *Corynebacterium aurimucosum*
DNS ∩ nc-wta ∩ nc-swab	1	*Cutibacterium acnes*
DNS ∩ nc-wta ∩ nc-wga	1	*Pseudomonas sp.*
DNS ∩ nc-wta ∩ nc-wga ∩ nc-library	1	*Escherichia coli*
DNS ∩ nc-wta ∩ nc-wga ∩ nc-swab	1	*Staphylococcus aureus*
DNS ∩ nc-wta ∩ nc-wga ∩ nc-swab ∩ nc-no-carries ∩ nc-library	1	*Burkholderia sp.*
nc-wta ∩ nc-with-carrie ∩ nc-no-carries	1	*Sphingomonas sp.*
nc-wta only	3	*Sphingomonas paucimobilis*; *Methylobacterium radiotolerans*; *Streptococcus mitis*
nc-swab only	5	*Kocuria rhizophila*; *Massilia timonae*; *Paenibacillus polymyxa*; *Brevibacterium linens*; *Acetobacter orientalis*
nc-with-carrie only	7	*Bradyrhizobium japonicum*; *Rhizobium leguminosarum*; *Paracoccus denitrificans*; *Novosphingobium aromaticivorans*; *Brevundimonas diminuta*; *Caulobacter vibrioides*; *Phenylobacterium zucineum*
nc-no-carries only	7	*Flavobacterium johnsoniae*; *Chryseobacterium indologenes*; *Elizabethkingia anopheles*; *Pedobacter heparinus*; *Chitinophaga pinensis*; *Acidovorax temperans*; *Herbaspirillum seropedicae*
nc-library only	2	*Enterobacter cloacae*; *Citrobacter freundii*
nc-wga only	0	/
DNS only	2120	Selected representative taxa: *Pasteurella multocida; Mycoplasma bovis; Mannheimia haemolytica; Mannheimia varigena; Moraxella bovis; Histophilus somni; Mycoplasma bovirhinis; Haemophilus parainfluenzae; Haemophilus haemolyticus; Moraxella boevrei; Haemophilus influenzae; Moraxella catarrhalis; Neisseria subflava; Klebsiella quasipneumoniae; Neisseria animaloris; Actinomyces sp.; Mycoplasma bovigenitalium; Burkholderia mallei; Acinetobacter baumannii; Mycobacterium avium; Mycobacterium timonense; Moraxella nasicaprae…*

Supplementary Fig. 1. Negative controls (NCs)-based evaluation of background bacterial signatures. (a) Overlap of bacterial taxa detected in deep nasopharyngeal swab (DNS) samples and six types of NCs. (b-e) Representative DNS/NCs-overlapped bacterial taxa showing distinct interpretation patterns based on contig representation and alignment support. (b) *Pseudomonas fluorescens* and (c) *Klebsiella pneumoniae* represent DNS-dominant DNS/NCs-overlapped taxa, showing greater contig representation and alignment support in DNS samples than in NCs. (d) *Fusobacterium sp.* represents an undetermined DNS/NC-overlapped taxon, with only

minor differences in contig representation and/or alignment quality between DNS and NCs samples. (e) *Sphingomonas aerolata* represents a potential NCs-associated background taxon, showing comparable or higher representation in NCs samples. Alignment length, identity, and coverage were compared between DNS and NCs samples. Bars indicate mean ± standard error of the mean (SEM), and each point represents one bacterial contig. NCs shown in panels b-e include whole-genome amplification control (nc-wga), environmental bench swab control (nc-swab), and nucleic acid extraction control with carrier (nc-with-carrier).

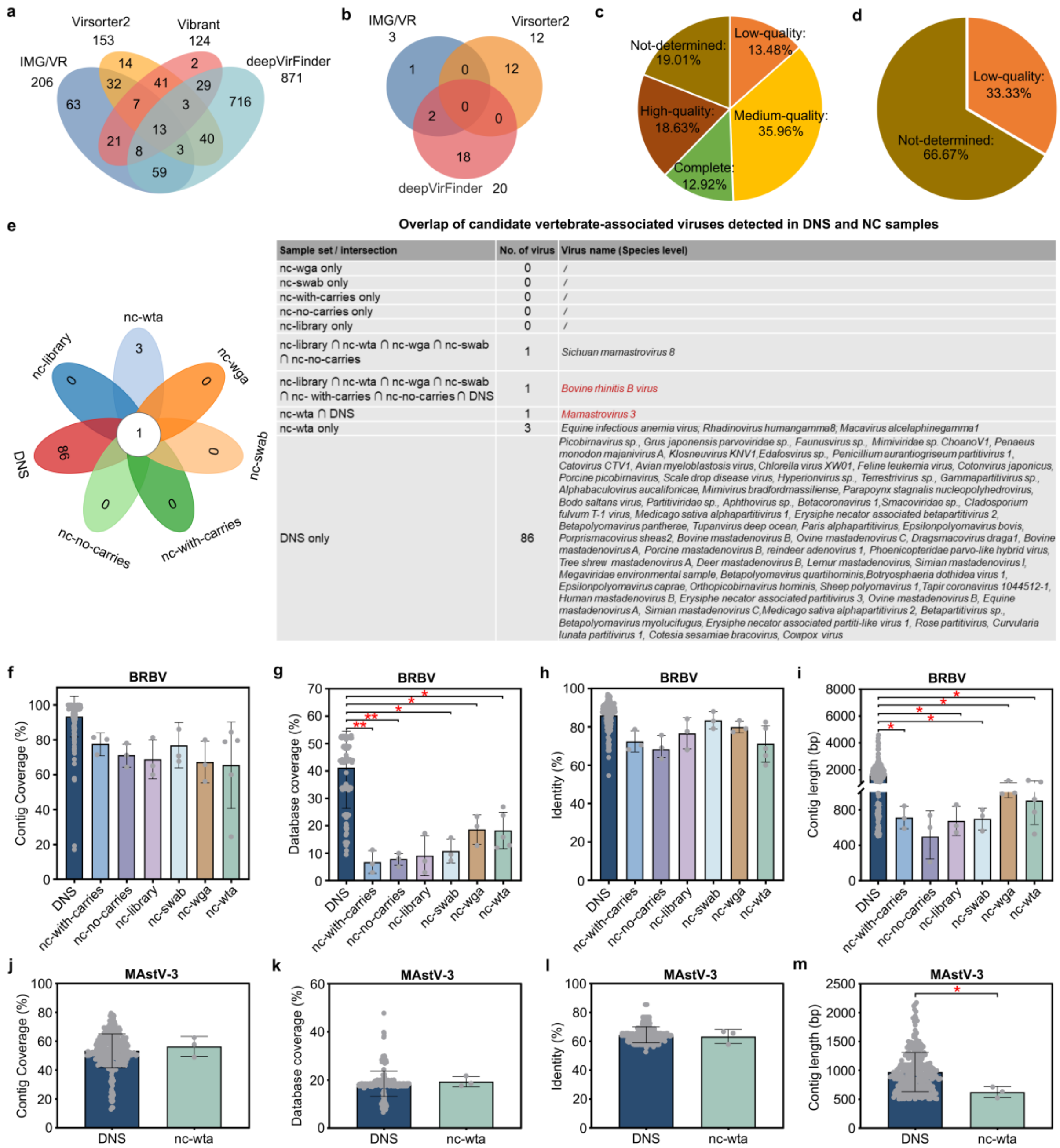

Sample set / intersection	No. of virus	Virus name (Species level)
nc-wga only	0	/
nc-swab only	0	/
nc-with-carries only	0	/
nc-no-carries only	0	/
nc-library only	0	/
nc-library ∩ nc-wta ∩ nc-wga ∩ nc-swab ∩ nc-no-carries	1	*Sichuan mamastrovirus 8*
nc-library ∩ nc-wta ∩ nc-wga ∩ nc-swab ∩ nc- with-carries ∩ nc-no-carries ∩ DNS	1	*Bovine rhinitis B virus*
nc-wta ∩ DNS	1	*Mamastrovirus 3*
nc-wta only	3	*Equine infectious anemia virus; Rhadinovirus humangamma8; Macavirus alcelaphinegamma1*
DNS only	86	*Picobirnavirus sp., Grus japonensis parvoviridae sp., Faunusvirus sp., Mimiviridae sp. ChoanoV1, Penaeus monodon majanivirus A, Klosneuvirus KNV1,Edafosvirus sp., Penicillium aurantiogriseum partitivirus 1, Catovirus CTV1, Avian myeloblastosis virus, Chlorella virus XW01, Feline leukemia virus, Cotonvirus japonicus, Porcine picobirnavirus, Scale drop disease virus, Hyperionvirus sp., Terrestrivirus sp., Gammapartitivirus sp., Alphabaculovirus aucalifonicae, Mimivirus bradfordmassiliense, Parapoynx stagnalis nucleopolyhedrovirus, Bodo saltans virus, Partitiviridae sp., Aphthovirus sp., Betacoronavirus 1,Smacoviridae sp., Cladosporium fulvum T-1 virus, Medicago sativa alphapartitivirus 1, Erysiphe necator associated betapartitivirus 2, Betapolyomavirus pantherae, Tupanvirus deep ocean, Paris alphapartitivirus, Epsilonpolyomavirus bovis, Porprismacovirus sheas2, Bovine mastadenovirus B, Ovine mastadenovirus C, Dragsmacovirus draga1, Bovine mastadenovirus A, Porcine mastadenovirus B, reindeer adenovirus 1, Phoenicopteridae parvo-like hybrid virus, Tree shrew mastadenovirus A, Deer mastadenovirus B, Lemur mastadenovirus, Simian mastadenovirus I, Megaviridae environmental sample, Betapolyomavirus quartihominis,Botryosphaeria dothidea virus 1, Epsilonpolyomavirus caprae, Orthopicobirnavirus hominis, Sheep polyomavirus 1,Tapir coronavirus 1044512-1, Human mastadenovirus B, Erysiphe necator associated partitivirus 3, Ovine mastadenovirus B, Equine mastadenovirus A, Simian mastadenovirus C,Medicago sativa alphapartitivirus 2, Betapartitivirus sp., Betapolyomavirus myolucifugus, Erysiphe necator associated partiti-like virus 1, Rose partitivirus, Curvularia lunata partitivirus 1, Cotesia sesamiae bracovirus, Cowpox virus*

Supplementary Fig. 2. Overview of viral contig identification, quality assessment, and NCs-based background evaluation. (a) Overlap of viral contigs identified from DNS samples using IMG/VR, VirSorter2, VIBRANT, and deepVirFinder. (b) Overlap of viral contigs identified from NCs samples using IMG/VR, VirSorter2, and deepVirFinder. (c) CheckV-based quality classification of viral contigs from DNS samples. (d) CheckV-based quality classification of viral contigs from NC samples. (e) Overlap of candidate vertebrate-associated viruses detected in DNS and six types of NC samples. (f-i) Comparison of *Bovine rhinitis B virus* (BRBV)-related contigs between DNS and NC samples based on contig coverage (%), database coverage (%), sequence identity, and contig length. (j-m) Comparison of *Mamastrovirus 3* (MAstV-3)-related contigs between DNS and nc-wta samples based on contig coverage (%), database coverage (%), sequence identity, and contig length. DNS, Deep nasopharyngeal swabs; NC, negative controls; Negative controls included whole-

transcriptome amplification (nc-wta), whole-genome amplification (nc-wga), an environmental (bench) swab control (nc-swab), nucleic acid extraction controls with and without carrier (nc-with-carrier and nc-no-carrier), and a library preparation PCR control (nc-library).

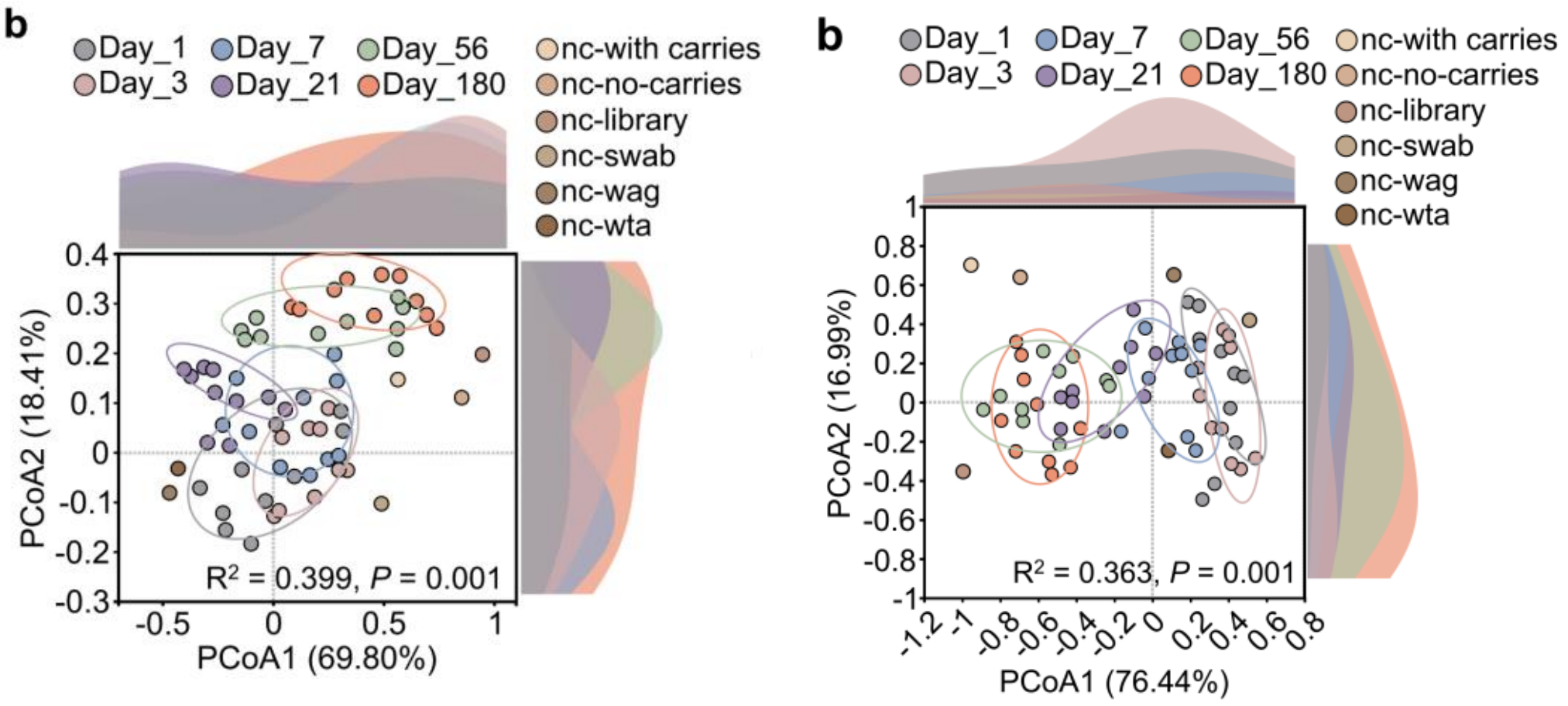

Fig. 2b and Supplementary Fig. 4b. PCoA analysis of DNS and NC samples. PCoA ordination of viral profiles (Fig. 2b, left) and bacterial profiles (Supplementary Fig. 4b, right), including DNS samples and six types of NC samples.

- Bioinformatics

I appreciate that the authors made their code available both for bioinformatics and statistical analysis. I want to congratulate the team for providing such well-organized and comprehensive code.

Replies: Thanks reviewer's positive comments.

- Data availability

Confirmed that PRJNA1259072 is available. Could not find PRJNA125959.

Replies: Thank you for pointing this out. The accession number contained a typographical error (a missing "0") and has been corrected to PRJNA1259590 in the revised manuscript (https://www.ncbi.nlm.nih.gov/bioproject/?term=PRJNA1259590).

- Statistical analysis

All statistical analyses are well described and appropriate.

- Results

Results are well described and not overinterpreted.

- Discussion

While this reviewer understands the challenges associated with working with low-biomass samples, the lack of negative controls is troubling. The findings align with what we know about the upper respiratory tract microbiome of bovines, but many taxa have also been reported as reagent contaminants. This limitation needs to be addressed in the discussion.

Replies: According to reviewer's comments, we have revised the Discussion section (lines 373-376 and 384-401) to address importance to include proper negative controls (NCs) to study low biomass samples and clarify how NCs-derived microbial signatures were evaluated. The revised text is as follows:

"Notably, appropriate NCs samples are essential for microbiome analyses of low-biomass samples, as background noise or contaminants may be introduced at multiple experimental steps, including nucleic acid extraction, amplification, library preparation, and sequencing. In

this study, background microbial signatures were detected across NCs representing different stages of the workflow, with relatively more evident features in amplification-related controls, particularly whole-transcriptome and whole-genome amplification. This suggests that amplification may be a key source of background microbial signatures in low-biomass samples. Due to the lack of well-established and validated criteria for interpreting NCs-associated microbial signatures, we applied branch-specific evaluation strategies for bacteria, phages, and vertebrate-associated viruses to distinguish potential technical background from biologically relevant signatures and to minimize both false-positive and false-negative interpretations. These observations indicate that NCs-detected microbial signatures in RT samples should not be interpreted using a simple presence/absence rule. Instead, microbial signatures shared between DNS and NC samples should be interpreted cautiously by considering contig-level patterns, such as contig representation and alignment quality, together with additional supporting evidence. These findings highlight the importance of including stage-specific NCs, especially amplification-related controls, in future low-biomass microbiome and virome studies."

- Conclusions
Well stated and in line with the data presented.

Figures:

Figure 1: typos – swabs not swaps, membrane not membrance
Response: Thank you for your comments. These have been corrected in revised Figure 1.

Reviewer #1 (Remarks on **code availability**):

I appreciate that the authors made their code available both for bioinformatics and statistical analysis. I want to congratulate the team for providing such well-organized and comprehensive code.
Response: We really appreciate reviewer's positive feedback.

Reviewer #2 (Remarks to the Author):

In this manuscript, the authors describe the characterization of the upper respiratory tract virome and microbiome of calves over the course of their first 180 days of life. The authors used a viral enrichment strategy coupled with reverse transcription and metagenomic sequencing to capture DNA and RNA viruses, as well as the bacterial community. The authors noted clear transitions in viral species composition and abundance over time, which reflected shifts in animal age, feeding, and living/space conditions.
Although this work is quite interesting and represents a novel contribution to the field in terms of the comprehensive time series characterization, I have concerns about the use of abundance/relative abundance data and their subsequent statistical analysis. Random amplification techniques can be prone to bias. The kit used here has been demonstrated to have GC bias with low biomass samples, and although it generally recapitulates rank-order abundances in mixed communities, relative abundances are not necessarily preserved in a consistent manner with communities of known composition (https://pmc.ncbi.nlm.nih.gov/articles/PMC5363268/#s13). Given these potential limitations, I would encourage the authors to consider a shift to rank-order metrics and parametric statistics or ask for additional demonstration that the random amplification step has not introduced bias

into these results. Additionally, I would encourage the authors to provide full citations for all of the bioinformatics tools and reference databases presented in the methods section. Although some are provided, many are missing.

Replies: In response to the reviewer's comments, we revised the analytical and statistical approaches by adopting a rank-based strategy to reduce reliance on relative abundance estimates. Parametric models were considered. However, the rank-based microbial profiles did not consistently meet the assumptions for parametric testing, likely due to the small longitudinal sample size and frequent zero values. We therefore used Friedman tests as a conservative repeated-measures approach for temporal comparisons. These revisions have been included into the revised Methods section (lines 628-630, 756-765 and 773-776), Results section (lines 137-138, 147-166, 179-181, and 208-209), Figures 2 and 3, Supplementary Figures 3-5, and the Supplementary Tables 16,17, 19, 21-23. The analysis scripts were also updated accordingly: the β-diversity partitioning script was revised (24_beta_diversity_partitioning_betapart. R), a new script for rank-order transformation of raw read-count profiles was added (27_read_count_rank_transformation. R), and the README file was updated to document the revised analytical workflow. In addition, missing citations for bioinformatics tools and reference databases have been added to the Methods section and reference list (References 58-75).

1. Revision of abundance-related analyses

(1) Statistical and analytical approaches

In the Methods section, we revised the description of the relevant analytical and statistical approaches as follows: "To reduce potential amplification biases associated with abundance estimation in low-biomass samples, downstream community and feature-level analyses were conducted using a zero-preserving rank-based approach. Raw read-count profiles were first aggregated at the corresponding taxonomic or functional level and then transformed using a zero-preserving rank-based approach[79]. Within each sample, only detected features were ranked based on raw read counts, while undetected features were retained as zero. The resulting rank-based score matrices were then used to support taxon-level temporal comparisons, viral family- and genome type-level summaries, heatmap visualization, and phage-host temporal trend analyses. Beta-diversity dissimilarities were calculated as 1-ρ based on Spearman's rank correlation, followed by PCoA." (Lines 756-765).

"Given the repeated-measures design, differences in α-diversity across time points were assessed using the Friedman test, followed by paired Wilcoxon signed-rank tests with Benjamini-Hochberg false discovery rate (FDR) correction." (Lines 773-776).

Reference:
79. Davis, M. L., Huang, Y. & Wang, K. Rank normalization empowers a t-test for microbiome differential abundance analysis while controlling for false discoveries. Brief. Bioinform. 22, bbab059 (2021).

(2) Analysis scripts

The analysis scripts were also updated accordingly. Specifically, the β-diversity partitioning script was revised (24_beta_diversity_partitioning_betapart. R), a new script for zero-preserving rank-order transformation of raw read-count profiles was added (27_read_count_rank_transformation. R), and the README file was updated to document the revised analytical workflow.

(3) Revised results

In the Results section, we revised Figures 2 and 3, Supplementary Figures 3-5, and

Supplementary Tables 16, 17, 19, 21-23 to align them with the rank-order-based analytical strategy. Specifically, **Figure 2** was updated to include rank-order-based β-diversity analysis and genome type-level rank-based detection scores, while detected vOTU numbers were used to describe viral colonization patterns without relying on relative abundance estimates (**Fig.2 b, d, f-i**). **Figure 3** was revised to present phage and their predicted bacterial host temporal dynamics using rank-based metrics. Heatmaps now show row Z-scores of rank-based scores, and host-phage temporal trajectories are shown as rank-based detection scores. The temperate and lytic phage panels were also updated to display mean rank-based scores across developmental stages (**Fig. 3b, c, f, h**). Supplementary heatmaps now use row Z-scores of rank-based scores (**Supplementary Figs. 3b and 4d**), and **Supplementary Fig. 5** shows temporal changes in rank-based detection scores for representative phage-host pairs. In addition, Supplementary Tables previously based on relative abundance were revised to report rank-based detection scores or summaries derived from rank-order transformed read-count profiles (**Supplementary Tables 16, 17, 19, 21-23**).

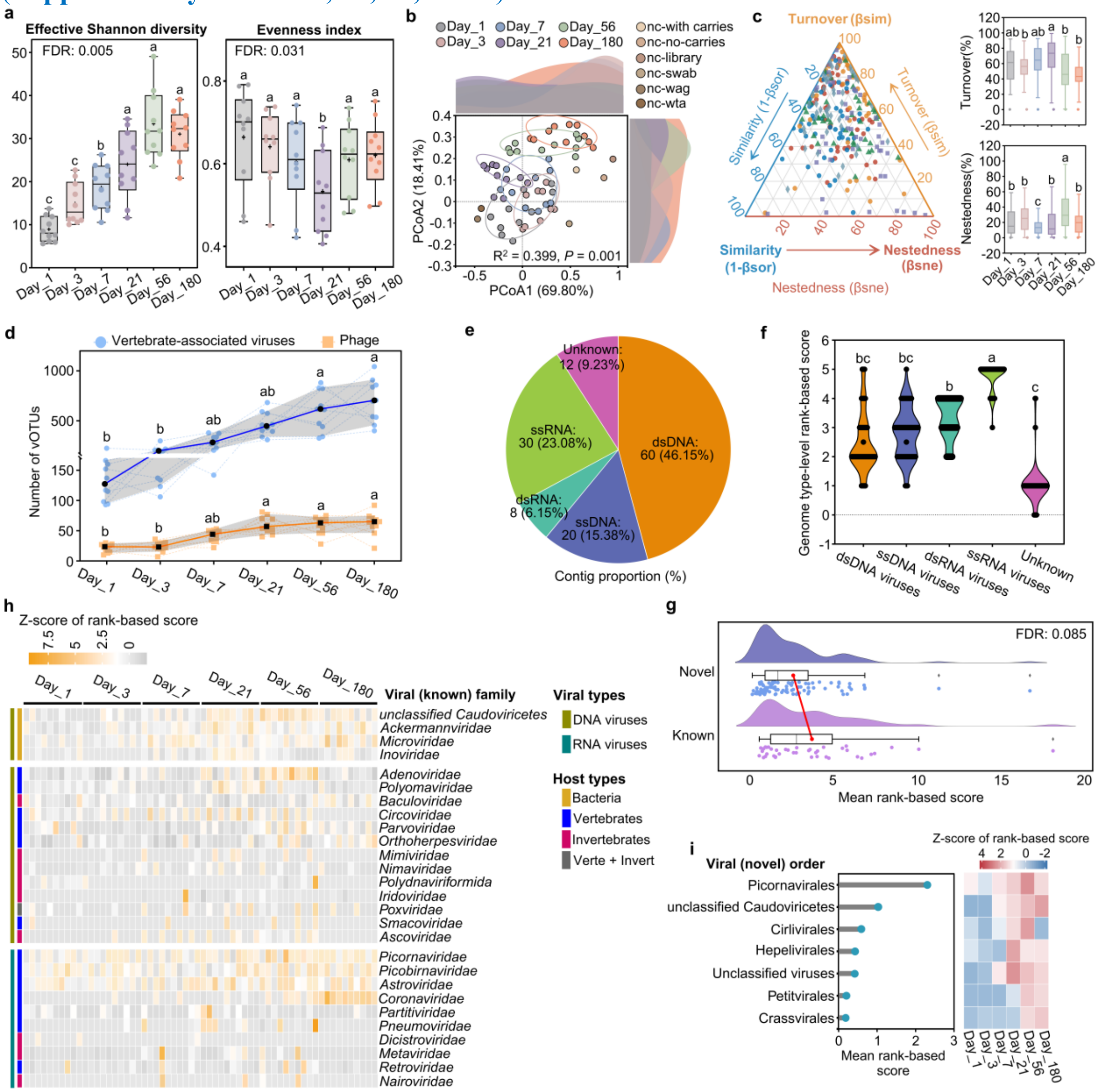

Figure 2. Overview of the URT virome in calves across early life. (a) Effective Shannon diversity and evenness index of the URT virome across six sampling time points. (b) Principal coordinate analysis (PCoA) based on rank-order dissimilarities showing temporal variation in URT viral profiles. (c) Partitioning of between-sample β-diversity in the calf URT virome. The ternary plot shows the contributions of similarity (1-β_sor), taxon turnover

(β_sim), and nestedness (β_sne) to pairwise community dissimilarity; boxplots summarize turnover and nestedness values for comparisons involving each sampling time point. (d) Number of detected phage and vertebrate-associated vOTUs across sampling time points. (e) Genome-type composition of viral contigs and (f) genome type-level rank-based scores across samples. (g) Comparison of mean rank-based scores between known and novel viral contigs. (h) Heatmap showing Z-scores of family-level rank-based scores for known viral families, annotated by viral genome type and host range. (i) Mean rank-based scores and temporal patterns of rank-based scores for putative novel viral orders. Rank-based scores were calculated from within-sample rankings of raw read counts, with undetected features retained as zero.

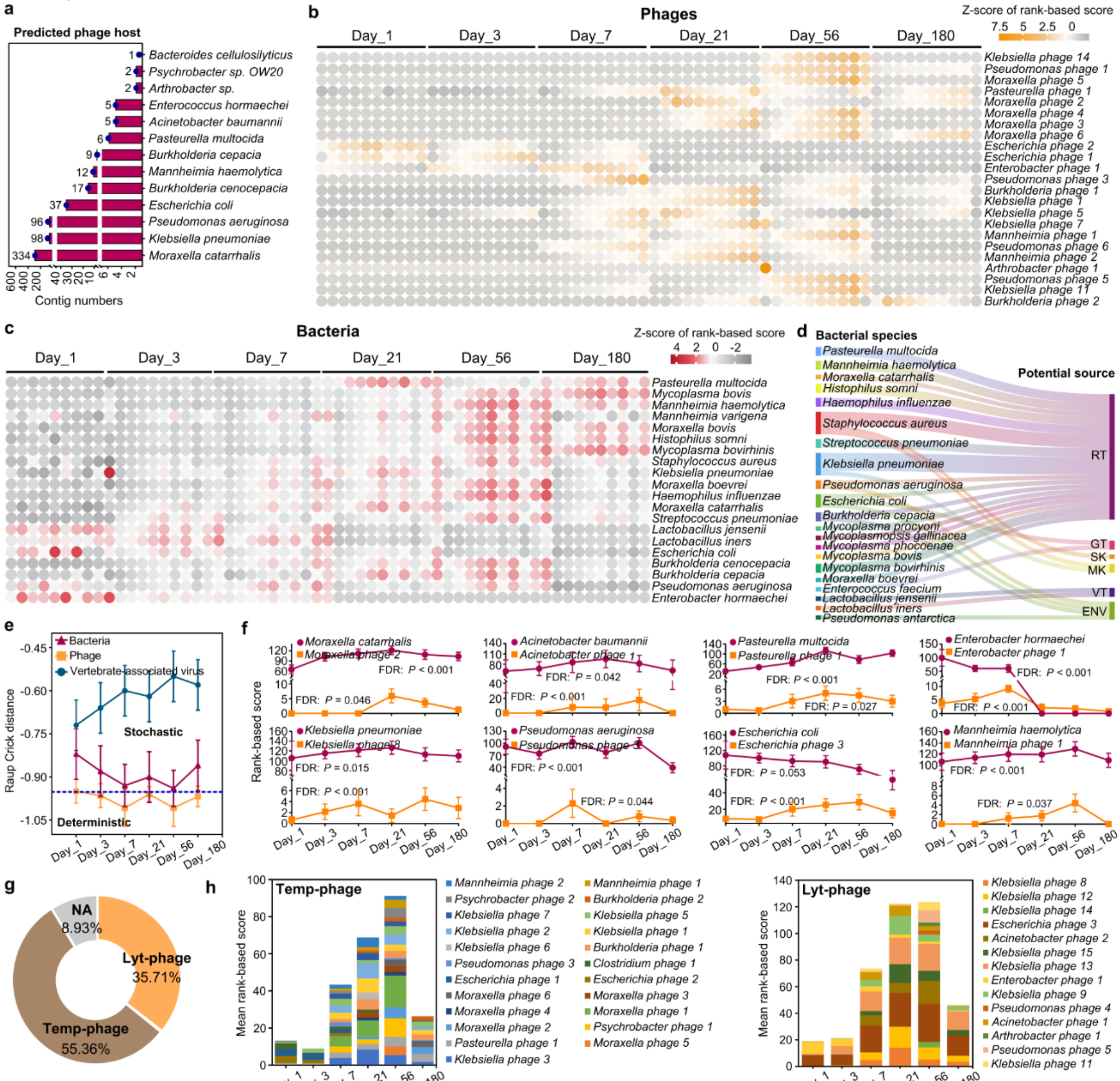

Figure 3. Host-dependent assembly and lifestyle-associated temporal dynamics of early-life URT phages in calves. (a) Predicted bacterial hosts of URT bacteriophages identified across early life. Bars indicate the number of phages contigs associated with each predicted bacterial host based on host prediction analysis. (b) Temporal dynamics of representative URT bacteriophages from birth to Day 180. Each dot represents the row Z-score of the rank-based score of an individual phage across sampling time points. (c) Temporal dynamics of representative bacterial taxa in the URT across early life, displayed as row Z-scores of rank-based scores. (d) Putative ecological associations of early-life URT bacterial taxa inferred from curated database information, illustrating taxa commonly associated with respiratory tract (RT), gastrointestinal tract (GT), skin (SK), milk (MK), vaginal tract (VT), and environmental (ENV) sources. (e) Raup-Crick dissimilarity analyses depicting community assembly processes for bacteria, bacteriophages, and vertebrate-associated viruses across early life. Values closer to the deterministic boundary indicate stronger deterministic structuring, whereas values near zero indicate stochastic assembly. (f) Representative temporal trajectories of rank-based scores for dominant bacterial taxa and their corresponding bacteriophages, highlighting coordinated host-phage dynamics across early life. (g) Proportional

composition of temperate (Temp-phage) and lytic (Lyt-phage) bacteriophages identified in the URT virome. (h) Temporal patterns of temperate and lytic bacteriophages across early life, shown as mean rank-based scores at each sampling time point.

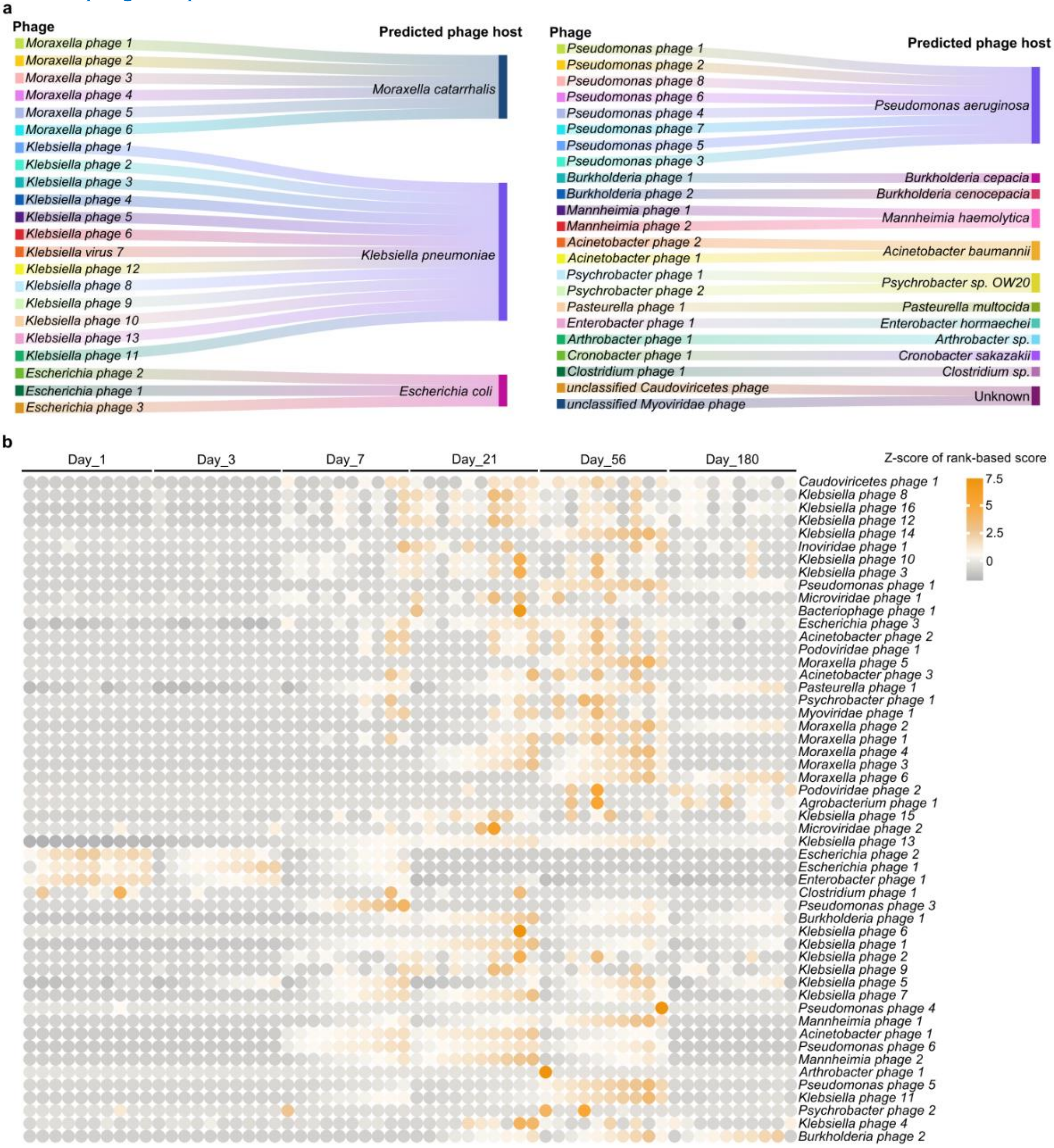

Supplementary Fig. 3. Temporal dynamics of representative calf URT phages and their predicted host associations. (a) Predicted host associations for representative phages identified in the calf URT. Sankey diagrams show links between individual phages on the left and their predicted bacterial hosts on the right. Phages without confident host assignments are labeled as "Unknown". (b) Heatmap showing temporal detection patterns of representative phages in the calf URT from birth to weaning at Days 1, 3, 7, 21, 56, and 180. Raw reads were first summarized at the phage species level within each sample and then transformed into rank-based scores, with zero values retained as zero. Mean rank-based scores were calculated for each developmental stage and standardized by row for visualization. Colors indicate the row Z-score of species-level rank-based scores.

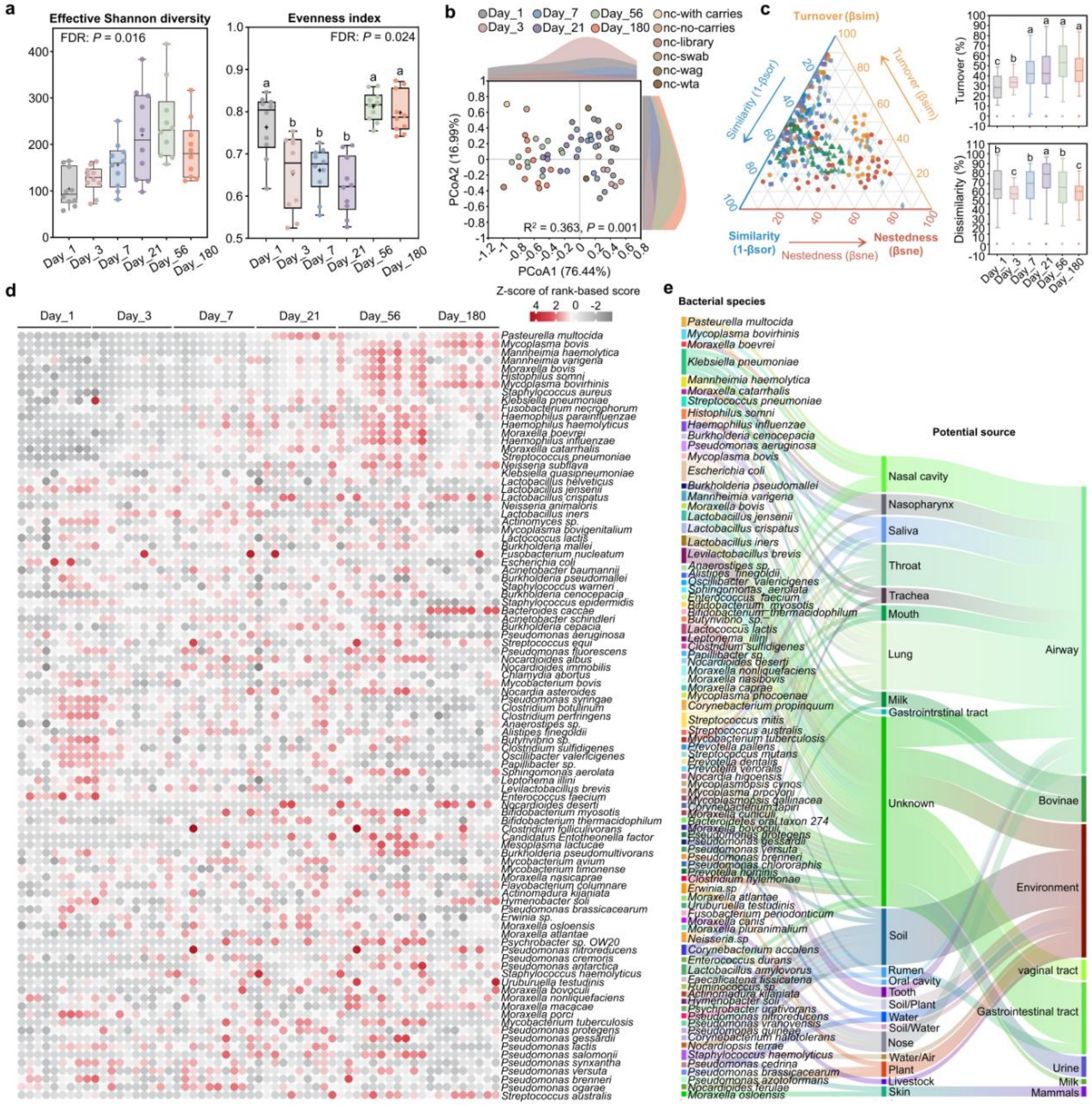

Supplementary Fig. 4. Early-life succession and potential sources of bacterial communities in the URT of beef calves. (a) Alpha diversity of URT bacterial communities across six sampling time points, including effective Shannon diversity and evenness index. Statistical significance was assessed using nonparametric tests with FDR correction. Different letters indicate significant differences between time points. (b) PCoA based on Spearman rank-correlation distances of within-sample rank-order-transformed raw read-count profiles, showing bacterial community dissimilarities across sampling time points and NCs. Each point represents one sample. (c) Ternary plot showing the relative contributions of similarity, turnover, and nestedness components to bacterial community β-diversity, with boxplots summarizing turnover and overall dissimilarity across sampling time points. (d) Heatmap showing temporal patterns of bacterial species across early life. Values represent Z-score-transformed rank-based scores within each bacterial species. (e) Sankey diagram showing the potential source of the bacterial species in the calves URT.

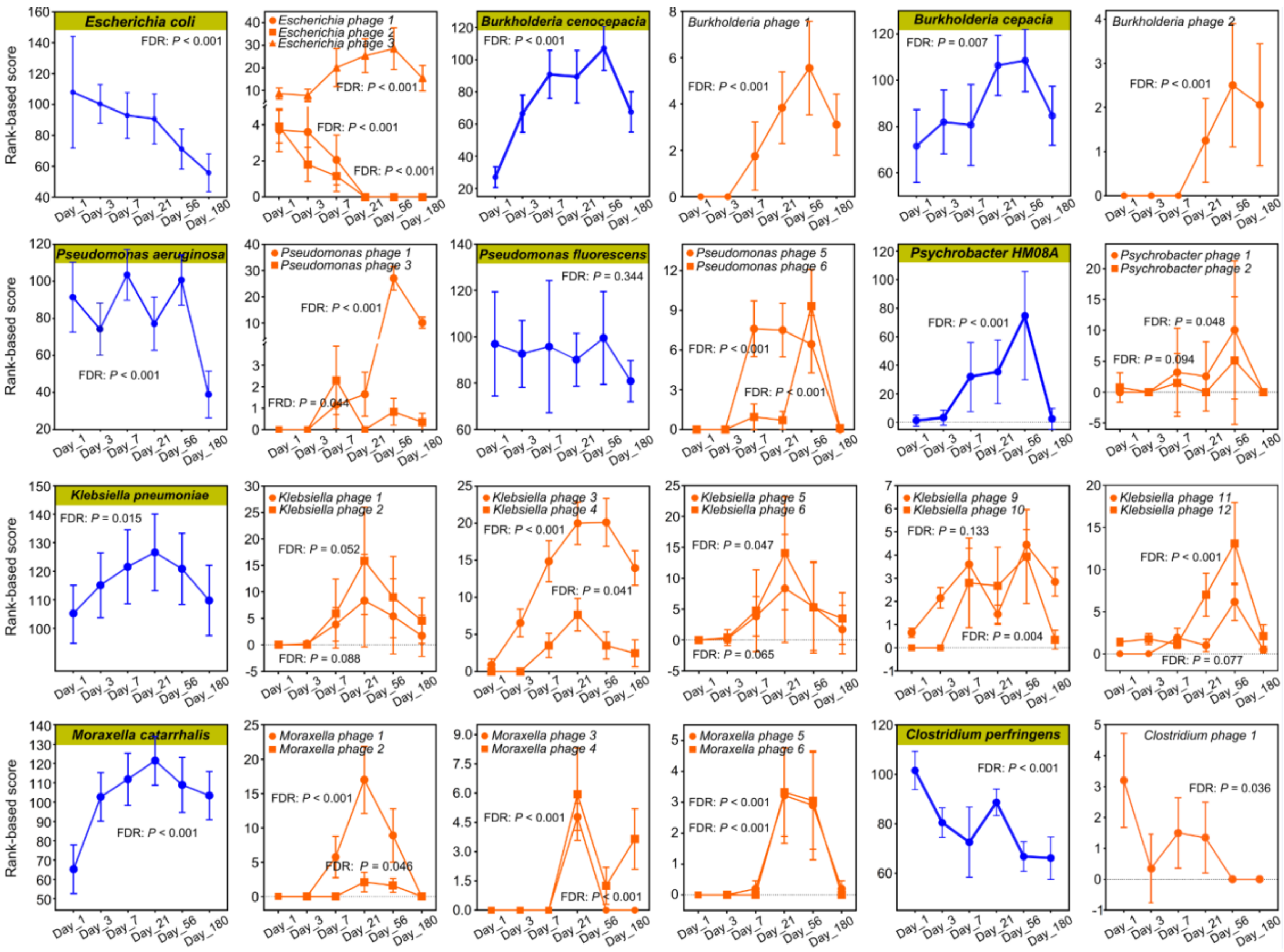

Supplementary Fig. 5. Temporal dynamics of dominant phages and their predicted bacterial hosts in the calf URT. Line plots show temporal changes in rank-based scores of representative phages and their predicted bacterial hosts from birth to weaning. Bacterial hosts are shown in blue, and associated phages are shown in orange. Rank-based scores were calculated from read-count profiles within each sample, with zero values retained as zero. Data are presented as mean ± SEM. Temporal trends were assessed using FDR-adjusted P-values, which are shown in each panel.

(3) Updated code and documentation

The analysis code and README file were updated accordingly. Specifically, the β-diversity partitioning script (24_beta_diversity_partitioning_betapart. R) was revised, a new script for raw read-count rank transformation (27_read_count_rank_transformation. R) was added, and the README file was updated to reflect the revised analytical workflow.

2. Added citations for bioinformatic tools and reference databases

The newly added references are listed below as References 58-75.

58. Brown, J., Pirrung, M. & McCue, L. A. FQC Dashboard: integrates FastQC results into a web-based, interactive and extensible FASTQ quality control tool. Bioinformatics 33, 3137-3139 (2017).
59. Li, H. & Durbin, R. Fast and accurate short read alignment with Burrows-Wheeler transform. Bioinformatics 25, 1754-1760 (2009).
60. Li, D., Liu, C. M., Luo, R., Sadakane, K. & Lam, T. W. MEGAHIT: an ultra-fast single-node solution for large and complex metagenomics assembly via succinct de Bruijn graph. Bioinformatics 31, 1674-1676 (2015).
65. Ewels, P. A. et al. The nf-core framework for community-curated bioinformatics pipelines. Nat. Biotechnol. 38, 276-278 (2020).
70. Li, H. et al. The Sequence Alignment/Map format and SAMtools. Bioinformatics 25, 2078-2079 (2009).

71. Prjibelski, A., Antipov, D., Meleshko, D., Lapidus, A. & Korobeynikov, A. Using SPAdes de novo assembler. Curr. Protoc. Bioinformatics 70, e102 (2020).
72. Hyatt, D., LoCascio, P. F., Hauser, L. J. & Uberbacher, E. C. Gene and translation initiation site prediction in metagenomic sequences. Bioinformatics 28, 2223-2230 (2012).
73. Letunic, I. & Bork, P. Interactive Tree of Life (iTOL) v6: recent updates to the phylogenetic tree display and annotation tool. Nucleic Acids Res. 52, W78-W82 (2024).
74. Ewels, P., Magnusson, M., Lundin, S. & Käller, M. MultiQC: summarize analysis results for multiple tools and samples in a single report. Bioinformatics 32, 3047-3048 (2016).
75. Langmead, B. & Salzberg, S. L. Fast gapped-read alignment with Bowtie 2. Nat. Methods 9, 357-359 (2012).

Reviewer #2 (Remarks on code availability):
The scripts used are detailed and well annotated. They could be reproduced.
Response: We really appreciate reviewer's positive feedback.

Responses to Reviewers' Comments

Reviewer #1 (Remarks to the Author):

The authors have appropriately addressed this reviewer's comments. This was a well written and relevant manuscript.

A few typos were noticed; please carefully review the manuscript, particularly the figures and axis labels.

Supplementary Figures 1 and 2 and all other figures:

nc-with-carries

nc-with-carrie

should be nc-with-carrier

Replies: Thank you for pointing this out. The typo has been corrected to "nc-with-carrier" in the manuscript (lines 566), Supplementary Figures 1 and 2, and Supplementary Tables 1-8.

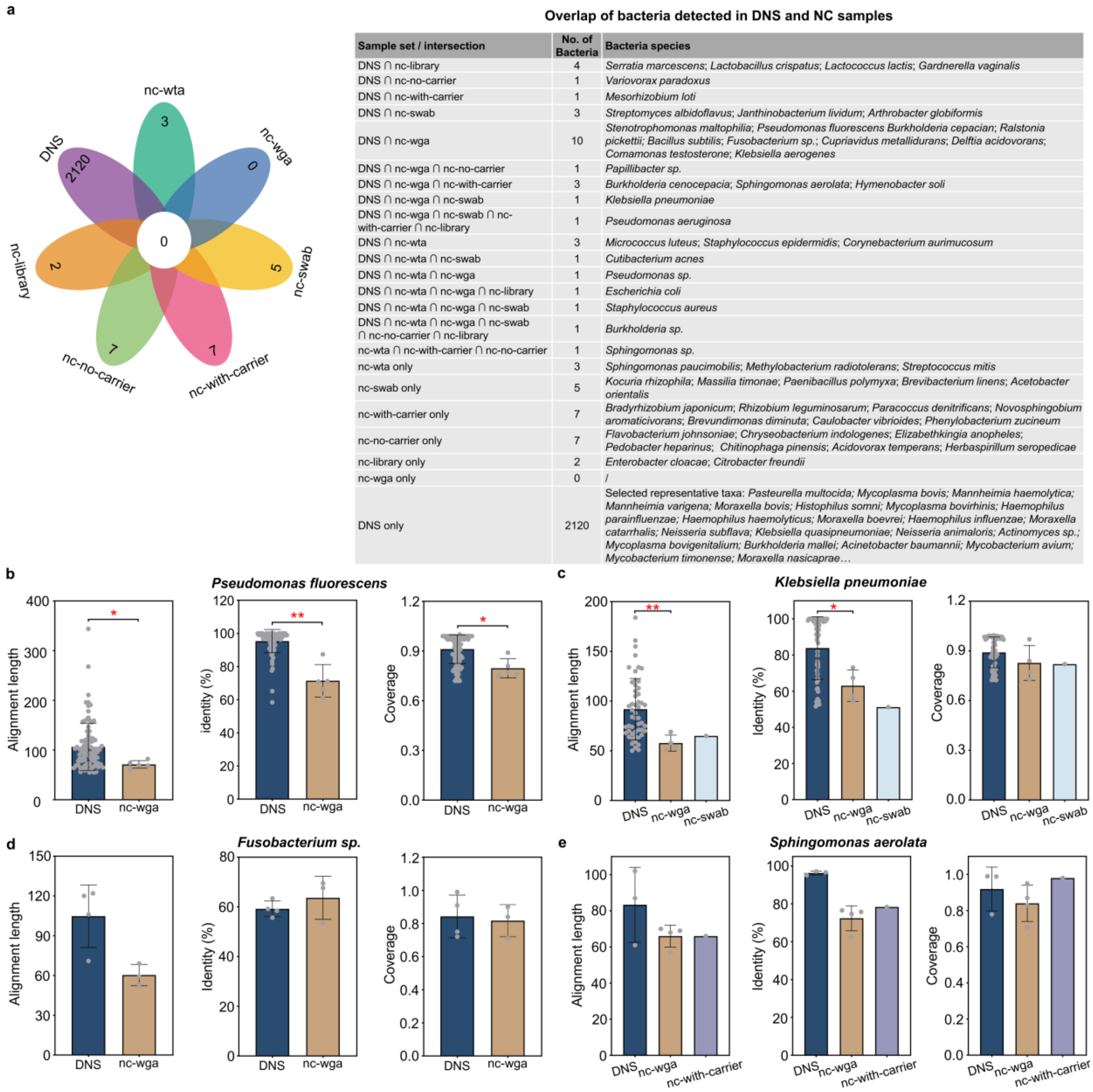

Sample set / intersection	No. of Bacteria	Bacteria species
DNS ∩ nc-library	4	*Serratia marcescens*; *Lactobacillus crispatus*; *Lactococcus lactis*; *Gardnerella vaginalis*
DNS ∩ nc-no-carrier	1	*Variovorax paradoxus*
DNS ∩ nc-with-carrier	1	*Mesorhizobium loti*
DNS ∩ nc-swab	3	*Streptomyces albidoflavus*; *Janthinobacterium lividum*; *Arthrobacter globiformis*
DNS ∩ nc-wga	10	*Stenotrophomonas maltophilia*; *Pseudomonas fluorescens Burkholderia cepacian*; *Ralstonia pickettii; Bacillus subtilis*; *Fusobacterium sp.*; *Cupriavidus metallidurans*; *Delftia acidovorans*; *Comamonas testosterone*; *Klebsiella aerogenes*
DNS ∩ nc-wga ∩ nc-no-carrier	1	*Papillibacter sp.*
DNS ∩ nc-wga ∩ nc-with-carrier	3	*Burkholderia cenocepacia*; *Sphingomonas aerolata*; *Hymenobacter soli*
DNS ∩ nc-wga ∩ nc-swab	1	*Klebsiella pneumoniae*
DNS ∩ nc-wga ∩ nc-swab ∩ nc-with-carrier ∩ nc-library	1	*Pseudomonas aeruginosa*
DNS ∩ nc-wta	3	*Micrococcus luteus*; *Staphylococcus epidermidis*; *Corynebacterium aurimucosum*
DNS ∩ nc-wta ∩ nc-swab	1	*Cutibacterium acnes*
DNS ∩ nc-wta ∩ nc-wga	1	*Pseudomonas sp.*
DNS ∩ nc-wta ∩ nc-wga ∩ nc-library	1	*Escherichia coli*
DNS ∩ nc-wta ∩ nc-wga ∩ nc-swab	1	*Staphylococcus aureus*
DNS ∩ nc-wta ∩ nc-wga ∩ nc-swab ∩ nc-no-carrier ∩ nc-library	1	*Burkholderia sp.*
nc-wta ∩ nc-with-carrier ∩ nc-no-carrier	1	*Sphingomonas sp.*
nc-wta only	3	*Sphingomonas paucimobilis*; *Methylobacterium radiotolerans*; *Streptococcus mitis*
nc-swab only	5	*Kocuria rhizophila*; *Massilia timonae*; *Paenibacillus polymyxa*; *Brevibacterium linens*; *Acetobacter orientalis*
nc-with-carrier only	7	*Bradyrhizobium japonicum*; *Rhizobium leguminosarum*; *Paracoccus denitrificans*; *Novosphingobium aromaticivorans*; *Brevundimonas diminuta*; *Caulobacter vibrioides*; *Phenylobacterium zucineum*
nc-no-carrier only	7	*Flavobacterium johnsoniae*; *Chryseobacterium indologenes*; *Elizabethkingia anopheles*; *Pedobacter heparinus*; *Chitinophaga pinensis*; *Acidovorax temperans*; *Herbaspirillum seropedicae*
nc-library only	2	*Enterobacter cloacae*; *Citrobacter freundii*
nc-wga only	0	/
DNS only	2120	Selected representative taxa: *Pasteurella multocida; Mycoplasma bovis; Mannheimia haemolytica; Mannheimia varigena; Moraxella bovis; Histophilus somni; Mycoplasma bovirhinis; Haemophilus parainfluenzae; Haemophilus haemolyticus; Moraxella boevrei; Haemophilus influenzae; Moraxella catarrhalis; Neisseria subflava; Klebsiella quasipneumoniae; Neisseria animaloris; Actinomyces sp.; Mycoplasma bovigenitalium; Burkholderia mallei; Acinetobacter baumannii; Mycobacterium avium; Mycobacterium timonense; Moraxella nasicaprae…*

Supplementary Fig. 1 Negative controls (NCs)-based evaluation of background bacterial signatures. (a) Overlap of bacterial taxa detected in deep nasopharyngeal swab (DNS) samples and six types of NCs. (b-e)

Representative DNS/NCs-overlapped bacterial taxa showing distinct interpretation patterns based on contig representation and alignment support. (b) *Pseudomonas fluorescens* and (c) *Klebsiella pneumoniae* represent DNS-dominant DNS/NCs-overlapped taxa, showing greater contig representation and alignment support in DNS samples than in NCs. (d) *Fusobacterium* sp. represents an undetermined DNS/NCs-overlapped taxon, with only minor differences in contig representation and/or alignment quality between DNS and NC samples. (e) *Sphingomonas aerolata* represents a potential NCs-associated background taxon, showing comparable or higher representation in NCs samples. Alignment length, identity, and coverage were compared between DNS and NCs samples. Bars indicate mean ± SEM, and each point represents one bacterial contig. Two-sided Wilcoxon rank-sum tests with FDR correction were used. *FDR-adjusted $P < 0.05$, **FDR-adjusted $P < 0.01$. NCs shown in panels (b-e) include whole-genome amplification control (nc-wga), environmental bench swab control (nc-swab), and nucleic acid extraction control with carrier (nc-with-carrier). Source data are provided as a Source Data file.

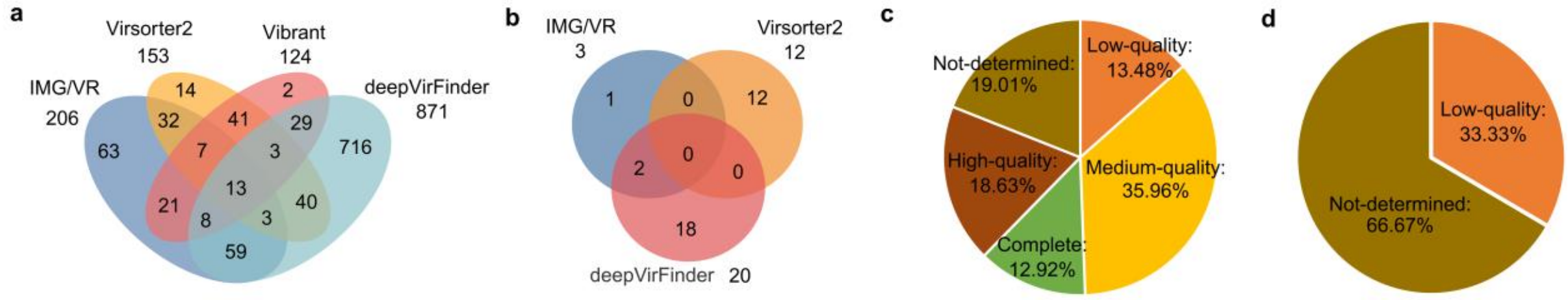

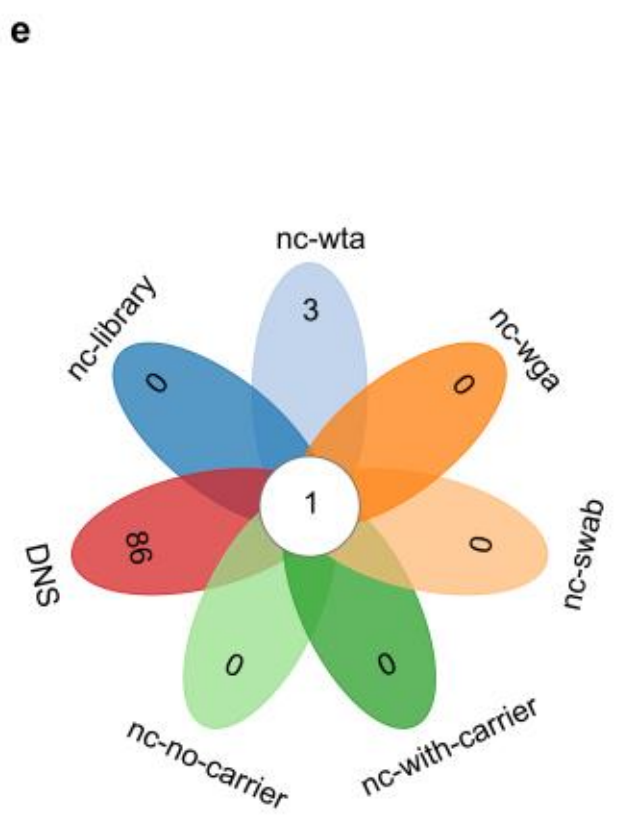

Overlap of candidate vertebrate-associated viruses detected in DNS and NC samples

Sample set / intersection	No. of virus	Virus name (Species level)
nc-wga only	0	/
nc-swab only	0	/
nc-with-carrier only	0	/
nc-no-carrier only	0	/
nc-library only	0	/
nc-library ∩ nc-wta ∩ nc-wga ∩ nc-swab ∩ nc-no-carrier	1	Sichuan mamastrovirus 8
nc-library ∩ nc-wta ∩ nc-wga ∩ nc-swab ∩ nc- with-carrier ∩ nc-no-carrier ∩ DNS	1	bovine rhinitis B virus
nc-wta ∩ DNS	1	Mamastrovirus 3
nc-wta only	3	equine infectious anemia virus; *Rhadinovirus humangamma8*; Macavirus alcelaphinegamma 1
DNS only	86	*Picobirnavirus* sp., Grus japonensis parvoviridae sp., *Faunusvirus* sp., *Mimiviridae* sp. ChoanoV1, Penaeus monodon majanivirus A, Klosneuvirus KNV1,*Edafosvirus* sp., Penicillium aurantiogriseum partitivirus 1, Catovirus CTV1, avian myeloblastosis virus, Chlorella virus XW01, feline leukemia virus, Cotonvirus japonicus, porcine picobirnavirus, scale drop disease virus, *Hyperionvirus* sp., *Terrestrivirus* sp., *Gammapartitivirus* sp., *Alphabaculovirus aucalifonicae*, Mimivirus bradfordmassiliense, Parapoynx stagnalis nucleopolyhedrovirus, Bodo saltans virus, *Partitiviridae* sp., *Aphthovirus* sp., *Betacoronavirus 1*, *Smacoviridae* sp., Cladosporium fulvum T-1 virus, Medicago sativa alphapartitivirus 1, Erysiphe necator associated betapartitivirus 2, *Betapolyomavirus pantherae*, Tupanvirus deep ocean, Paris alphapartitivirus, *Epsilonpolyomavirus bovis*, Porprismacovirus sheas2, bovine mastadenovirus B, bovine mastadenovirus C, Dragsmacovirus draga1, bovine mastadenovirus A, porcine mastadenovirus B, reindeer adenovirus 1, Phoenicopteridae parvo-like hybrid virus, tree shrew mastadenovirus A, deer mastadenovirus B, lemur mastadenovirus, simian mastadenovirus I, *Megaviridae* environmental sample, *Betapolyomavirus quartihominis*, Botryosphaeria dothidea virus 1, *Epsilonpolyomavirus caprae*, *Orthopicobirnavirus hominis*, sheep polyomavirus 1, tapir coronavirus 1044512-1, human mastadenovirus B, Erysiphe necator associated partitivirus 3, ovine mastadenovirus B, equine mastadenovirus A, simian mastadenovirus C, Medicago sativa alphapartitivirus 2, *Betapartitivirus* sp., *Betapolyomavirus myolucifugus*, Erysiphe necator associated partiti-like virus 1, rose partitivirus, Curvularia lunata partitivirus 1, Cotesia sesamiae bracovirus, cowpox virus

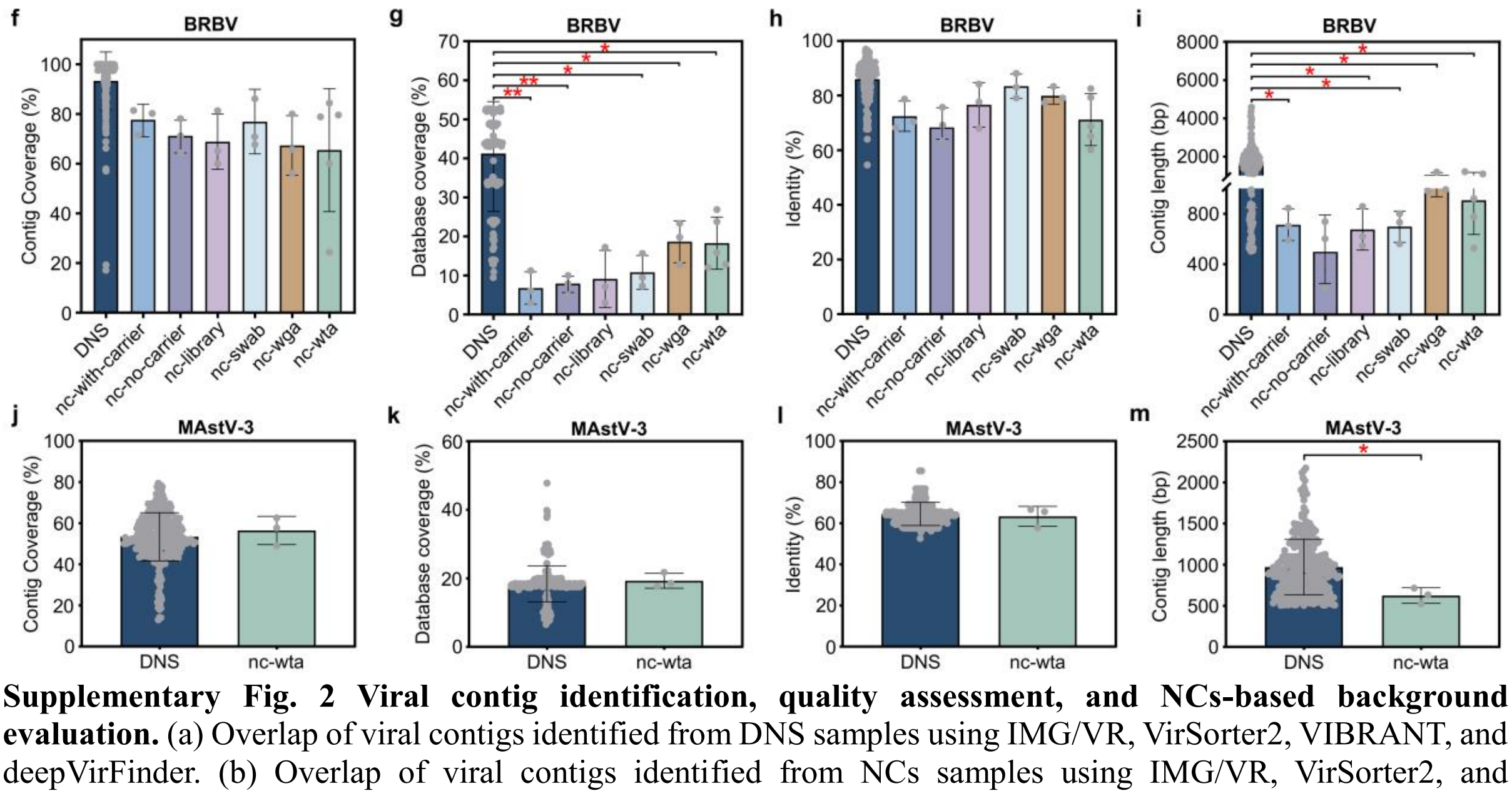

Supplementary Fig. 2 Viral contig identification, quality assessment, and NCs-based background evaluation. (a) Overlap of viral contigs identified from DNS samples using IMG/VR, VirSorter2, VIBRANT, and deepVirFinder. (b) Overlap of viral contigs identified from NCs samples using IMG/VR, VirSorter2, and deepVirFinder. (c) CheckV-based quality classification of viral contigs from DNS samples. (d) CheckV-based quality classification of viral contigs from NCs samples. (e) Overlap of candidate vertebrate-associated viruses

detected in DNS samples and six types of NCs. (f-i) Comparison of bovine rhinitis B virus (BRBV)-related contigs between DNS and NCs samples based on contig coverage, database coverage, sequence identity, and contig length. (j-m) Comparison of Mamastrovirus 3 (MAstV-3)-related contigs between DNS and whole-transcriptome amplification control (nc-wta) samples based on contig coverage, database coverage, sequence identity, and contig length. Bars indicate mean ± SEM, and each point represents one viral contig. Statistical comparisons were performed using two-sided Wilcoxon rank-sum tests with FDR correction. *FDR-adjusted $P < 0.05$, **FDR-adjusted $P < 0.01$. NCs included whole-transcriptome amplification control (nc-wta), whole-genome amplification control (nc-wga), environmental bench swab control (nc-swab), nucleic acid extraction controls with and without carrier (nc-with-carrier and nc-no-carrier), and library preparation PCR control (nc-library). Source data are provided as a Source Data file.

Reviewer #3 (Remarks to the Author):

Thanks for the edits and the response to the reviewers' comments. I have a few minor comments.

Please note that a virus name and species name are two different things. Just as for example Joe Brown is a name of a person but is a member of the species Homo sapiens. Thus, Bovine rhinitis B virus, Bovine gammaherpesvirus 4 and Human gammaherpesvirus 8 are a virus names and not a species and should not be italicized, and the first letter in human, bovine should not be capital letter.

Furthermore, in the case of virus taxonomy, all taxa names (expect virus name) are italicized. Please italicize Picornavirales, Caudoviricetes, Cirlivirales, Embecovirus.

Reviewer #3 (Remarks on code availability):
na

Replies: Thanks for this helpful clarification. We have revised the formatting of virus names and taxonomic names throughout the manuscript (lines 120), and Supplementary Figure 2e, as well as Supplementary Tables 15-18. Specifically, virus names are now presented in non-italic font, with lowercase initial letters for descriptive host terms such as "bovine," "human", "porcine" and "avian" etc. when they appear within a sentence. Formal viral taxonomic names are italicized. The taxonomic names specifically noted by the reviewer, including *Picornavirales*, *Caudoviricetes*, *Cirlivirales*, and *Embecovirus*, have been italicized in the manuscript (lines 165-166, 280, 283, and 292) and Figure 2i.

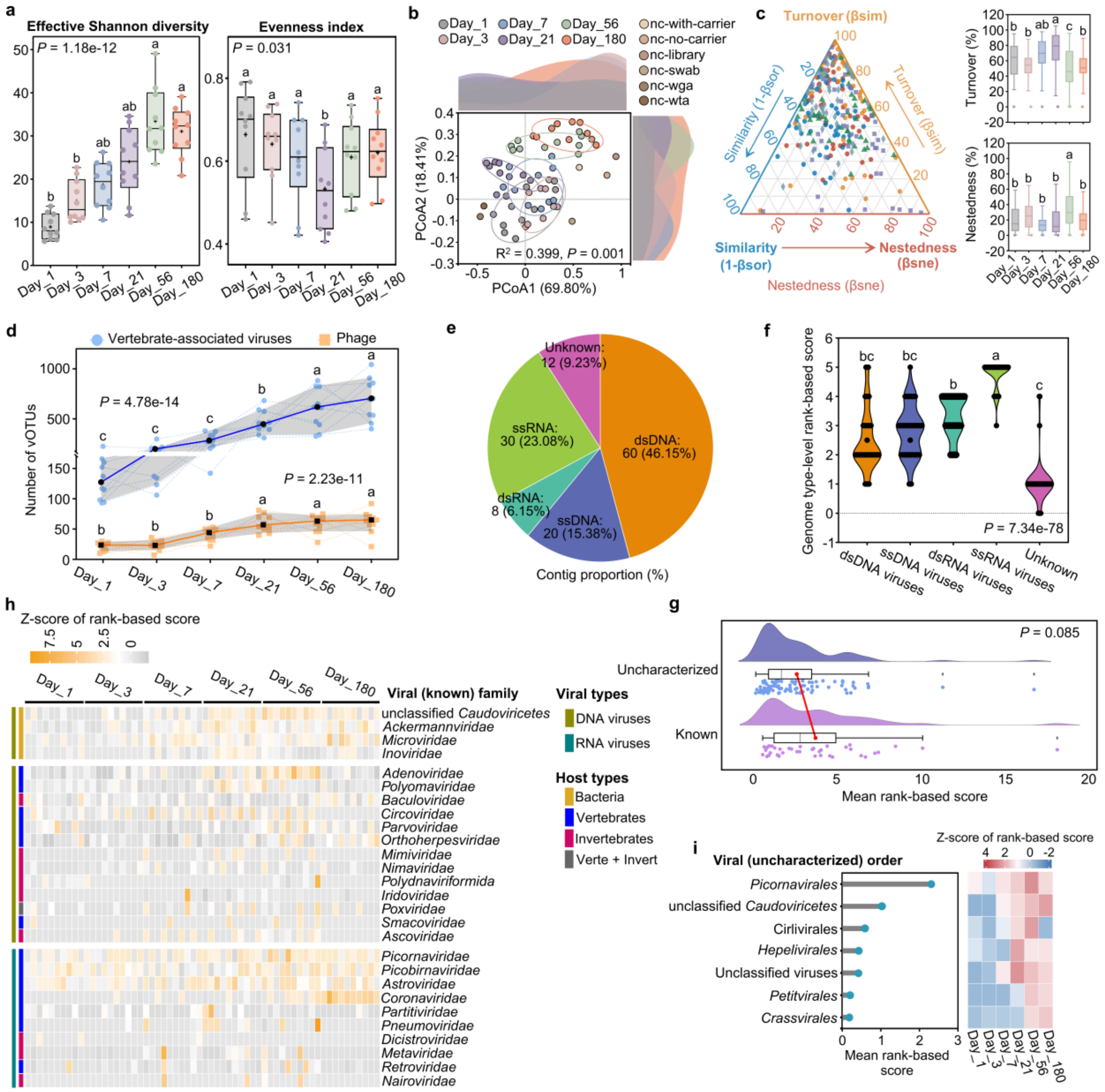

Figure 2. Overview of the upper respiratory tract (URT) virome in calves across early life.
(a) Effective Shannon diversity and evenness index across six sampling time points. Each point represents one biologically independent deep nasopharyngeal swab (DNS) sample; n = 10 calves per time point. Box plots show the median, 25th-75th percentiles, and whiskers extending to 1.5× interquartile range (IQR). (b) Principal coordinate analysis (PCoA) based on Spearman rank-correlation distances calculated from rank-order-transformed raw read-count profiles. Each point represents one sample; n = 60 DNS samples and n = 6 negative control (NC) samples. The effect of sampling time on DNS viral profiles was tested using PERMANOVA with 999 permutations constrained by calf identity. (c) Partitioning of beta diversity into similarity, turnover, and nestedness components using within-time-point pairwise comparisons. The ternary plot includes n = 270 pairwise comparisons in total; box plots summarize n = 45 comparisons per time point and are defined as in a. Beta diversity was partitioned into Sørensen dissimilarity (β_sor), turnover (β_sim), and nestedness-resultant components (β_sne) using the betapart package. (d) Number of detected phage and vertebrate-associated viral operational taxonomic units (vOTUs) across sampling time points. Points represent biologically independent DNS samples; n = 10 calves per time point. Lines show median values and shaded areas indicate 95% confidence intervals (CI). (e) Genome-type composition of viral contigs. (f) Genome type-level rank-based scores across DNS sample profiles. Violin plots show distributions, with internal box plots defined as in (a). Each point represents one DNS sample-level score for the indicated genome type; n = 60 DNS samples per genome type. Differences among genome types were tested using Friedman tests followed by Dunn's multiple-comparison tests. Different letters indicate significant differences between genome types after multiple-comparison correction. (g) Mean rank-based scores of known (n = 40) and uncharacterized (n = 89) contigs. Each point represents one contig. Differences were tested using a two-tailed Mann-Whitney test. (h) Z-scores of family-level rank-based scores for known viral families,

annotated by viral genome type and host range. (i) Mean rank-based scores and temporal patterns of rank-based scores for uncharacterized viral order-level groups. Rank-based scores were calculated from within-sample rankings of raw read counts, with undetected features retained as zero. Time-point differences in (a), (c) and (d) were tested using Friedman tests followed by Dunn's post hoc multiple comparisons, with Benjamini-Hochberg FDR correction. Different letters indicate significant differences between time points; exact *P* values of (a), (c), (d), (f) and (g) are provided in the Source Data file. Source data are provided as a Source Data file.
