# Supplementary material for: Early-life bovine respiratory virome analysis reveals phage-driven adaptation and cross-host evolutionary signatures
Source: Nat Commun. 2026 Oct 28;17:11185. doi: 10.1038/s41467-026-77597-8 (PMC13714303; doi:10.1038/s41467-026-77597-8)
Supplement: Supplementary file 4 [file 41467_2026_77597_MOESM2_ESM.docx]

**Description of Additional Supplementary Files**

**Title:** Supplementary Data 1

**Description:** Raw metagenomic sequencing read counts and quality metrics for deep nasopharyngeal swab (DNS) samples from beef calves and negative controls, including total reads, total bases, Q20, Q30, and GC content.

**Title:** Supplementary Data 2

**Description:** Quality-filtered metagenomic sequencing read counts and quality metrics for DNS samples from beef calves and negative controls, including clean reads, total bases, Q20, Q30, and GC content.

**Title:** Supplementary Data 3

**Description:** Contig-level metagenomic assembly statistics for DNS samples from beef calves and negative controls, including contig number and length, N50, N90, and maximum and minimum contig lengths.

**Title:** Supplementary Data 4

**Description:** Raw virome sequencing read counts and quality metrics for DNS samples from beef calves and negative controls, including total reads, total bases, Q20, Q30, and GC content.

**Title:** Supplementary Data 5

**Description:** Quality-filtered virome sequencing read counts and quality metrics for DNS samples from beef calves and negative controls, including clean reads, total bases, Q20, Q30, and GC content.

**Title:** Supplementary Data 6

**Description:** Assembly statistics for virome contigs from DNS samples of beef calves and negative controls, including contig number and length, N50, N90, and maximum and minimum contig lengths.

**Title:** Supplementary Data 7

**Description:** NCBI non-redundant (NR) protein database-based taxonomic annotations of filtered bacterial contigs from DNS samples of beef calves and negative controls, with alignment length, sequence identity, coverage, and E-value information.

**Title:** Supplementary Data 8

**Description:** Classification of bacterial taxa detected in both DNS samples of beef calves and negative controls, with comparisons of contig counts, alignment lengths, sequence identities, and coverage values.

**Title:** Supplementary Data 9

**Description:** Candidate viral contigs identified by comparison with IMG/VR, including contig length, uncultivated virus genome and viral operational taxonomic unit identifiers, ecosystem information, and taxonomic annotations.

**Title:** Supplementary Data 10

**Description:** Candidate viral contigs identified using VirSorter2, with the predicted viral sequence type for each contig.

**Title:** Supplementary Data 11

**Description:** Candidate viral contigs identified using VIBRANT, with predicted lifestyle and genome quality classifications.

**Title:** Supplementary Data 12

**Description:** Candidate viral contigs identified using DeepVirFinder, including contig length, prediction score, P value, and q value.

**Title:** Supplementary Data 13

**Description:** CheckV-based quality and completeness assessment of viral contigs from DNS samples of calves and negative controls, including provirus status, host-gene content, completeness, and contamination estimates.

**Title:** Supplementary Data 14

**Description:** Summary statistics for viral operational taxonomic unit (vOTU) representative sequences retained in the bacteriophage analysis branch, including total sequence length, sequence number, length distribution, and GC content.

**Title:** Supplementary Data 15

**Description:** Taxonomic annotations of animal virus contigs from DNS samples of beef calves and negative controls based on searches against the NCBI nucleotide (NT) and non-redundant (NR) virus databases, with alignment statistics and taxonomic assignments.

**Title:** Supplementary Data 16

**Description:** Temporal changes in family-level rank-based scores of bacteriophages in DNS samples from beef calves identified using VITAP, with lineage scores, confidence levels, standard errors, P values, and false discovery rate (FDR)-adjusted *P* values.

**Title:** Supplementary Data 17

**Description:** Temporal changes in rank-based scores of animal virus families in DNS samples from beef calves, with standard errors, *P* values, and FDR-adjusted *P* values.

**Title:** Supplementary Data 18

**Description:** PhaBOX-based taxonomic classification, host prediction, and lifestyle annotation of bacteriophage contigs.

**Title:** Supplementary Data 19

**Description:** Temporal changes in genus-level rank-based scores of bacteriophages in DNS samples from beef calves identified using VITAP, with lineage scores, confidence levels, standard errors, *P* values, and EDR-adjusted *P* values.

**Title:** Supplementary Data 20

**Description:** Chi-square test results comparing deterministic and stochastic community assembly proportions inferred from Raup-Crick dissimilarity across the analyzed groups.

**Title:** Supplementary Data 21

**Description:** Temporal changes in rank-based detection scores of the most frequently detected bacterial phyla in DNS samples from beef calves, with standard errors, *P* values, and FDR-adjusted *P* values.

**Title:** Supplementary Data 22

**Description:** Temporal changes in rank-based detection scores of bacterial genera in DNS samples from beef calves, with standard errors, *P* values, and FDR-adjusted *P* values.

**Title:** Supplementary Data 23

**Description:** Temporal changes in rank-based detection scores of the most frequently detected bacterial species in DNS samples from beef calves, with standard errors, *P* values, and FDR-adjusted *P* values.

**Title:** Supplementary Data 24

**Description:** Virulence Factor Database (VFDB)-based annotation of virulence factors in bacterial communities from DNS samples of beef calves, including bacterial taxonomy, virulence factor identity and function, and functional category.

**Title:** Supplementary Data 25

**Description:** Relative contributions of bacterial taxa to virulence factor functions over time, with standard errors, *P* values, and FDR-adjusted *P* values.

**Title:** Supplementary Data 26

**Description:** VFDB-based annotation of bacteriophage-associated virulence factors in DNS samples from beef calves, including viral taxonomy, virulence factor identity and function, and functional category.

**Title:** Supplementary Data 27

**Description:** Relative contributions of bacteriophages to virulence factor functions in DNS samples from beef calves over time, with standard errors, *P* values, and FDR-adjusted *P* values.

**Title:** Supplementary Data 28

**Description:** Mobile genetic element (MGE) database-based functional annotations of bacterial communities in DNS samples from beef calves, including bacterial taxonomy and mobile genetic element identity and type.

**Title:** Supplementary Data 29

**Description:** Relative contributions of bacterial taxa to MGE functions over time, with standard errors, *P* values, and FDR-adjusted *P* values.

**Title:** Supplementary Data 30

**Description:** MGE database-based functional annotations of bacteriophages in DNS samples from beef calves, including viral taxonomy and MGE identity and type.

**Title:** Supplementary Data 31

**Description:** Relative contributions of bacteriophages to MGE functions over time, with standard errors, *P* values, and FDR-adjusted *P* values.

**Title:** Supplementary Data 32

**Description:** Read-mapping summary statistics for the bovine-derived CoV-D180 genome sequence and representative human and bovine betacoronaviruses based on competitive alignment of human respiratory metatranscriptomic datasets, including mapped-read counts and sequencing-depth metrics.

**Title:** Supplementary Data 33

**Description:** Read-mapping summary statistics for the bovine-derived adenovirus-like genome sequence AdV-D56 and representative human and bovine adenoviruses based on competitive alignment of human respiratory metatranscriptomic datasets, including mapped-read counts and sequencing-depth metrics.

**Title:** Supplementary Data 34

**Description:** BLASTx-based identification of RNA-dependent RNA polymerase (RdRp) proteins encoded by RNA virus contigs using the NCBI RdRp protein database, with query and subject coverage, sequence identity, alignment coordinates, E values, and scores.

**Title:** Supplementary Data 35

**Description:** InterProScan-based identification and annotation of RdRp domains encoded by RNA virus contigs, including database accession, domain name, description, and source database.

**Title:** Supplementary Data 36

**Description:** BLASTx-based identification of conserved proteins encoded by animal DNA virus contigs, with sequence identity, alignment coordinates, E values, and scores.

**Title:** Supplementary Data 37

**Description:** InterProScan-based identification and annotation of conserved domains encoded by vertebrate-associated DNA virus contigs, including database accession, domain name, description, and source database.
